# Benzophenone Oxime Tosylate as the Photoacid Generator for the Friedel–Crafts Arylation of Aldehydes With Indoles

**DOI:** 10.1002/chem.202503021

**Published:** 2025-12-10

**Authors:** Michael Gkosios, Paraskevi Papatasou, Anastasia Maria Antonaki, Petros L. Gkizis

**Affiliations:** ^1^ Laboratory of Organic Chemistry, Department of Chemistry Aristotle University of Thessaloniki University Campus Thessaloniki Greece

**Keywords:** BIMs, Friedel–Crafts arylation, inimosulfonates, photoacid generators, photochemistry

## Abstract

Photochemistry enables transformations inaccessible through ground‐state pathways. We report a sustainable and operationally simple Friedel–Crafts arylation using benzophenone oxime tosylate, in low catalyst loading, as the photoacid generator under inert conditions. The method accommodates a broad substrate scope, affording diarylmethane derivatives in good to excellent yields. Mechanistic studies elucidate key intermediates driving the transformation.

## Graphical abstract

Friedel–Crafts Arylation of Indoles: Use of an iminosulfonate derivative as the photoacid generator for the Friedel–Crafts arylation reaction between aldehydes and indoles under inert atmosphere.

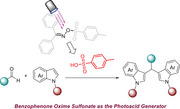

## 1 Introduction

**SCHEME 1:**
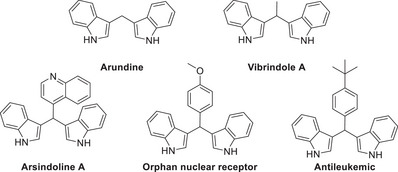
BIMs with biological activity.

Indole scaffolds are prevalent in numerous pharmaceutically active compounds and natural products of significant biological importance [1, 2]. Benzylindole motifs (BIMs) exhibit antileukemic activity and can also function as orphan nuclear receptors [3]. Arundine, a dimeric alkaloid isolated from the roots of *Arundo donax*, has shown therapeutic potential against breast cancer [4], while Vibrindole A has been investigated for the treatment of chronic fatigue [5]. The indole moiety is also present in natural products such as Arsindolide A, derived from marine bacteria (Scheme 1) [6]. Owing to these diverse bioactivities, indole‐containing molecules have attracted considerable attention [1, 2].

**SCHEME 2:**
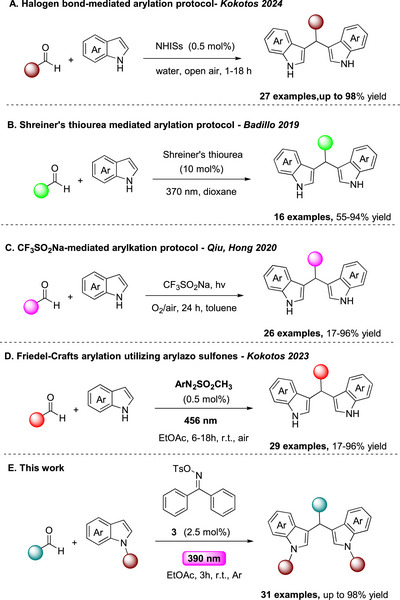
Friedel–Crafts arylation protocols and this work.

The Friedel–Crafts coupling reaction is a powerful strategy for aromatic functionalization [7, 8, 9], and has proven particularly effective for the construction of diarylmethane derivatives [10]. Traditional protocols, however, often rely on Lewis or Brønsted acids, requiring high temperatures or toxic reagents—conditions poorly implemented to pharmaceutical applications [11, 12, 13, 14, 15, 16]. To address these challenges, sustainable methodologies have been introduced, including the use of green solvents, solvent‐free conditions, microwave chemistry, sonochemistry [17, 18, 19, 20, 21, 22, 23, 24], organocatalysis [25], and electrochemistry [26]. In addition, halogen‐bond‐based approaches have been explored to replace toxic reagents and avoid strongly acidic environments (Scheme 2A) [27, 28, 29].

Over the past decade, the pioneering works of MacMillan [30], Yoon [31], and Stephenson [32] have reignited interest in the long‐overlooked field of Photochemistry. Since then, light‐driven methodologies have expanded dramatically [33, 34, 35, 36, 37, 38, 39], as the unique reactivities unlocked by photon interactions have reshaped strategies in modern Organic Synthesis. In 2019, Badillo and co‐workers reported an elegant photochemical protocol exploiting Schreiner's thiourea to promote a photoacidic process under blue LED irradiation (Scheme 2B) [40]. The main limitations of this methodology are the prolonged reaction time (18 h) and the catalyst synthesis, which increases the overall cost of the process. Subsequently, Qiu, Hong, and coworkers developed a mild photocatalyst‐free protocol for BIMs synthesis via in situ generation of CF_3_ radicals from CF_3_SO_2_Na, where oxygen played a crucial role in facilitating CF_3_SO_2_Na degradation (Scheme 2C) [41]. More recently, in 2023, Kokotos and coworkers reported a rapid and versatile visible‐light‐driven Friedel–Crafts coupling of aldehydes with (hetero)arenes using arylazo sulfones as photoacid generators (Scheme 2D) [42]. Beyond this transformation, arylazo sulfones have found broad application in synthesis and materials chemistry, particularly as nonionic PAGs capable of releasing methanesulfonic acid under oxygen‐rich or air‐equilibrated conditions [43, 44, 45, 46, 47]. All the above‐mentioned protocols are evident of the essential role of oxygen in generating active intermediates. However, this requirement restricts the substrate scope by excluding oxygen‐sensitive molecules. Moreover, gas evolution, such as nitrogen release from arylazo sulfones, poses additional drawbacks from an industrial perspective.

In 2021, Glorius and co‐workers reported a metal‐free oxyimination of inactivated alkenes using a bifunctional oxime carbonate derivative [48]. Light‐induced N–O cleavage produced both *N*‐ and *O*‐centered radicals, enabling radical addition. Inspired by this approach, we presume that a tosylated benzophenone oxime derivative (**3**), upon irradiation, could generate a tosyloxy radical readily converted into *p*‐toluenesulfonic acid, thereby catalyzing Friedel–Crafts arylation in the absence of oxygen. Iminosulfonates are well‐established nonionic PAGs [49] that undergo homolytic N─O cleavage upon irradiation, affording sulfonyloxy radicals that typically yield sulfonic acids after hydrogen abstraction from the solvent. Such species are widely applied in cationic photopolymerization [50] but their use in Organic Synthesis is merely studied.

Our group has long been engaged in photochemistry and photoredox catalysis [51, 52], focusing on the development of green, sustainable protocols amenable to industrial application [53]. Building on these advances, we sought to establish a mild photochemical protocol for the synthesis of diarylmethanes, introducing oxime sulfonates as photoacid generators (Scheme 2E). Herein, we report a mild, green, and catalyst‐free Friedel–Crafts‐type coupling of aldehydes with indole derivatives, providing efficient access to valuable intermediates for pharmaceutical applications.

## 2 Results and Discussion

We initiated our efforts to probe the optimal reaction conditions, choosing as the model reaction; the reaction between benzaldehyde (**1a**) and indole (**2a**) (Table 1) [54]. Our effort to develop a catalyst‐free protocol generating an acid from the tosyl oxime (**3**), led us to examine the impact of the employed irradiation source toward the homolytic cleavage of the N─O bond [54].

**TABLE 1:** Optimization of the reaction conditions for the photochemical reaction between benzaldehyde (1a) or hexanal (1p) and indole (2a).

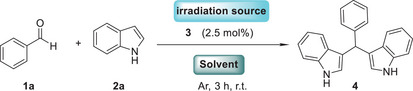			
Entry	Lamps [nm]	Solvent [mL]	Yield [%]^[a]^
1	370	AcOEt	95 (90)
2b^[b]^	370	AcOEt	70 (63)
**3**	**390**	**AcOEt**	**100 (98)**
**4** ^[b]^	**390**	**AcOEt**	**100 (85)**
5	427	AcOEt	92 (88)
6^[b]^	427	AcOEt	85 (80)
7	390	MeOH	81
8	390	CH_2_Cl_2_	80
9	390	Petroleum Ether	72
10	390	Toluene	60
11	390	THF	63
12	390	Et_2_O	66
13	390	H_2_O	70

^[a]^ Yield was determined by ^1^H‐NMR using internal standard. Yield of isolated product after column chromatography is presented in parenthesis.

^[b]^ The reaction was performed using hexanal (**1p**) instead of benzaldehyde (**1a**).

Entries 3 & 4 refer to the optimal reaction conditions.

Gratifyingly, efficient N─O bond cleavage occurred under purple LED irradiation (370–390 nm) (Table 1, entries 1–4). In particular, UVA irradiation at 370 nm afforded the desired product in 90% yield with complete consumption of benzaldehyde (**1a**) (Table 1, entry 1). The best outcome (98% yield) was obtained at 390 nm (Table 1, entry 3), while comparable results were achieved under 427 nm irradiation (88%, Table 1, entry 5). In contrast, longer‐wavelength sources led to a significant decrease in yield [54]. To further assess the optimal wavelength, the same reaction conditions were applied to an aliphatic aldehyde; hexanal (**1p**). In this case, 390 nm irradiation again proved superior, validating it as the optimal light source (Table 1, entries 1–6).

Next, we continued our studies in identifying the optimum reaction medium for the photochemical Friedel–Crafts protocol (Table 1) [54]. Use of polar aprotic solvents, such as acetonitrile afforded the desired product in mediocre yield [54]. Switching to other polar aprotic solvents, such as ethyl acetate, proceeded in excellent yields (Table 1, entry 3). Contrariwise, when DMSO or *N,N*’‐dimethylformamide (DMF) were exploited, the reaction did not take place [54]. Slightly lower reaction yields were obtained when chlorinated solvents, such as dichloromethane or chloroform, afforded the desired product in good yield (Table 1, entry 8) [54]. In the presence of a nonpolar solvent, like petroleum ether, the reaction took place in good yield (Table 1, entry 9). The reaction yield was slightly decreased, when the reaction was conducted in toluene (Table 1, entry 10). Ethereal solvents promote the reaction leading to mediocre reaction yields (Table 1, entries 11 and 12). Interestingly, the use of water afforded promising results, highlighting its potential as a green solvent; however, isolation of the product proved challenging, lowering the overall yield (70%, Table 1, entry 13). Next, we conducted experiments to prompt the optimum reaction concentration, and we concluded using 0.5 mL of ethyl acetate per 0.2 mmol of **1a** [54]. Control experiments confirmed that both UVA irradiation and an inert atmosphere are essential for the reaction to proceed [54].

**SCHEME 3:**
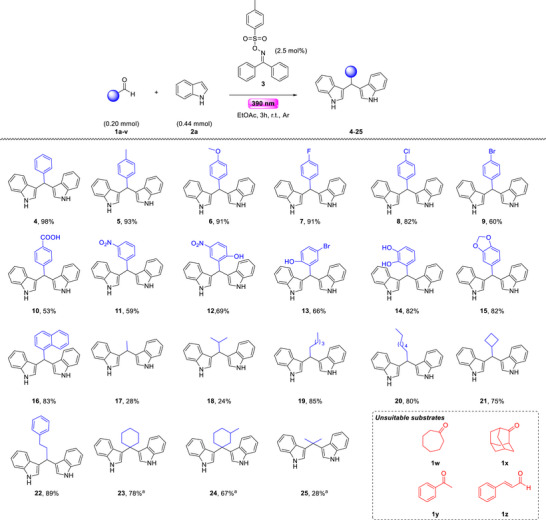
Substrate scope. Reaction time was prolonged to 18 h.

Having in hand the optimum reaction conditions, we shifted our interest to studying the reaction scope, employing the batch reaction technology introduced by Noël and coworkers (Scheme 3) [54, 55]. Initially, we explored the reaction of indole (**2a**) with a variety of aromatic aldehydes (**1a**–**m**). Electron‐donating substituents, such as methyl or methoxy group, led to the formation of the desired products in excellent yields (Scheme 3, **5** and **6**). Halogen incorporation on the benzaldehyde moiety was well tolerated, leading to the corresponding fluoro, chloro, and bromo derivatives in excellent yields (Scheme 3, **7**–**9**). Electron‐withdrawing groups, such as carboxy‐ and nitro‐ delivered the desired products **10** and **11** in mediocre yields, 53% and 59% respectivetly. When the aromatic ring was decorated with more than one substituents, good to excellent yields were attained (Scheme 3, **12**–**14**). Furthemore, when bicyclic or fused aromatic aldehydes were used, the reaction procedeed smoothly and the diaryl methane derivatives were formed in excellent yields (Scheme 3, **15** and **16**). Next, we applied our photochemical protocol to a variety of aliphatic aldehydes (Scheme 3, **17**–**22**). Acetaldehyde (**1n**) and isobutyraldehyde (**1ο**) led to poor reaction yields (Scheme 3, **17** and **18**). This can be attributed to volatile substrates **1n** and **1o**, which were not detected in the reaction mixture upon reaction completion. Additionally, no byproduct formation was observed. Heavier aliphatic aldehydes, such as hexanal (**1p**), octanal (**1q**), cyclobutyl carboxaldehyde (**1r**), and 3‐phenylpropanal (**1s**) reacted excellently, providing access to diaryl derivatives **19–22** in excellent yields. Especially, when cyclobutyl carboxaldehyde (**1r**) was tested, neither decarbonylation reaction nor ring cleavage was observed, forming the desired product (**21**) in excellent yield (75%). In the case where 3‐phenyl‐propanal (**1s**) was tested, the benzylic position remained intact (Scheme 3, **22**).

Seeking to broaden the substrate scope, a variety of ketones was also put to the test. In this case, the reaction time was prolonged to 18 h, for the full consumption of the ketone derivative. Cyclohexanone proved to be an excellent choice, forming the desired product (**23**) in good yield (78%). Comparable yield (67%) was obtained when 3‐methylcyclohexanone was tested (Scheme 3, **24**). On the other hand, acetone proved to be a weak choice due to its volatile character, leading to poor reaction yields. In accordance, use of other ketones, such as cycloheptanone, adamantanone, or even acetophenone did not lead to the desired products (Scheme 3, **1w**–**1y**). It should, also, be noted that the application of our proposed photocemical reaction conditions to a naturally occuring molecule, like cinnamaldehyde (**1z**), was not met with efficiency. Instead, formation of byproducts derived either from the [2+2] cycloaddition or the E/Z isomerization process were observed. Such side reaction triggering could be attributed to the benzophenone molecule generated upon catalyst‘s (**3**) degradation.

**SCHEME 4:**
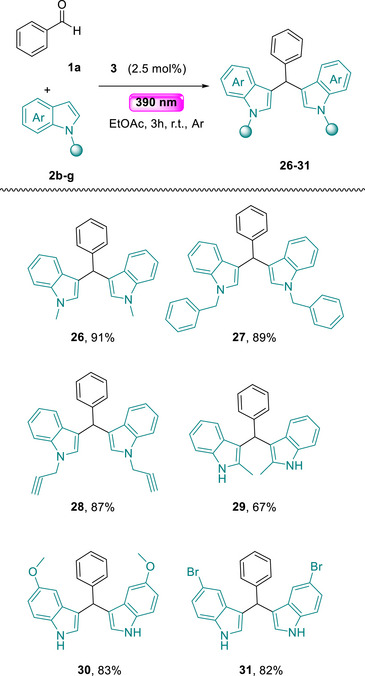
Substrate scope—substituted indoles.

Since the substrate scope of the carbonyl agent was investigated, we moved to testing the scope of indoles in our photochemical conditions (Scheme 4) [54]. Thus, variety of *N*‐substituted indoles were functionalized making use of benzaldehyde (**1a**) as the reaction partner. Initially, the substitution pattern on the nitrogen of the indole was probed. Simple *N*‐alkyl substituents, like methyl, benzyl, or propargyl were well tolerated, leading to the desired products **26–28** in good to excellent yields (Scheme 4). Sterically hindered 2‐methyl indole was proved to act as a competent nucleophile, providing access to **29** in 67% yield (Scheme 4). Indoles decorated with methoxy group or a bromine atom on the benzene ring also lead to the desired products in good yields (Scheme 4, **30–31**).

**SCHEME 5:**
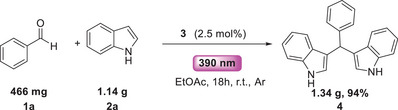
Gram scale reaction between benzaldehyde (**1a**) and indole (**2a**).

To further demonstrate the industrial feasibility and scalability of our protocol, we conducted the Friedel–Crafts arylation using natural sunlight as the irradiation source, replacing the 390 nm Kessil lamp. The reaction was performed under sunlight (November 14, 2025 09:00–15:00, Thessaloniki, Greece, 40.37° N. 22.57° E, temperature 12–18°C) and delivered the desired product in excellent yield (90%) [54]. To compare the impact of natural sunlight with Kessil lamps, we measured the reaction conversion by ^1^H‐NMR after 3 h, finding an 80% reaction conversion. In addition, we performed reaction between benzaldehyde (**1a**) and indole (**2a**) in gram scale, leading to an excellent yield of 94% after 18 h (Scheme 5) [54].

### 2.1 Mechanistic Studies

To elucidate the reaction pathway and the degradation of oxime sulfonate (**3**), we conducted a series of mechanistic experiments. UV–Vis spectroscopy was first employed to probe the possible formation of an electron donor–acceptor (EDA) complex. Consistent with Melchiorre's seminal studies [56, 57, 58], such complexes can initiate photochemical transformations.

**SCHEME 6:**
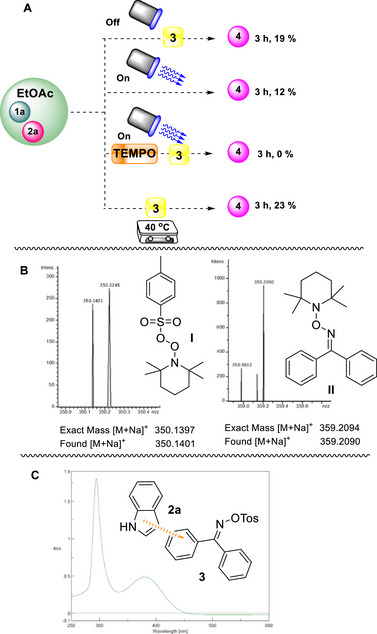
Mechanistic and control experiments.

**SCHEME 7:**
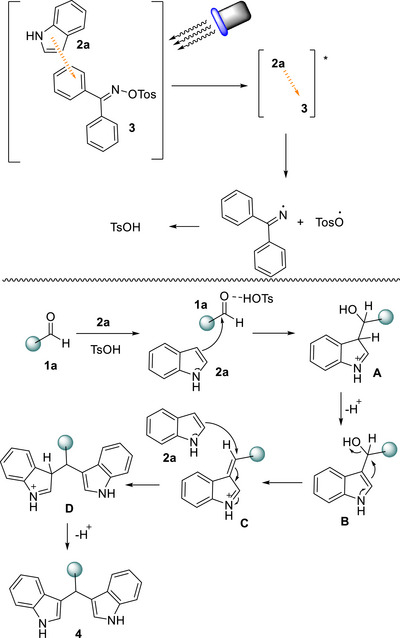
Proposed reaction mechanism.

Mixing indole (**2a**) with oxime sulfonate (**3**) in our system, produced a new absorption band at 390 nm, indicative of EDA complex formation (Scheme 6C) [54]. Similar interactions between indoles and aryls are well documented [56, 57, 58]. Additionally, use of radical scavengers, such as TEMPO, verified the radical character of the generated radicals that form the acidic environment in the reaction mixture, since the reaction was totally suppressed (Scheme 6A) [54]. To further confirm the reaction mechanism, Direct‐infusion high‐resolution mass spectrometry (DI‐HRMS) of a reaction mixture containing benzaldehyde (**1a**), indole (**2a**), and TEMPO revealed signals corresponding to adducts **I** and **II**, consistent with the generation of a tosyloxy radical and imine radical via N─O bond cleavage of diphenylmethanone O‐tosyl oxime (**3**), (Scheme 6B) [54]. Based on our findings, a plausible mechanism for the amino‐chlorination protocol is depicted in Scheme 7. When mixing benzaldehyde (**1a**), indole (**2a**), and catalyst (**3**) an EDA complex is formed between indole (**2a**) and catalyst (**3**). Upon LED irradiation (390 nm), excitation of the EDA complex [**2a**‐**3**] leads to the homolysis of the nitrogen‐oxygen bond of the oxime moiety, generating *N*‐centered radical and *O*‐centered radical [TsO**^.^**]. The latter forms *p*‐toluenesulfonic acid which protonates the carbonyl group of the carbonyl derivative, enhancing its electrophilic character and facilitating the nucleophilic attack of indole (**1a**), forming intermediate **A**. Aromatization of **A** leads to **B,** which upon protonation forms iminium intermediate **C**. **C** reacts with a second molecule of indole (**2a**), which upon aromatization delivers the desired product (**4**) (Scheme 7).

## 3 Conclusions

In conclusion, we developed a simple, easy‐to‐execute and mild photochemical protocol for the activation of the carbonyl group in both aldehydes and ketones toward the reaction with substituted indoles forming diarylmethanes. This method relies on the ability of benzophenone oxime tosylate to slowly release acid, under LED irradiation, activating the carbonyl group. The formation of an EDA complex between indoles and benzophenone oxime tosylate facilitates the N─O cleavage, generating *p*‐toluene sulfonic acid. A broad scope of substrates was successfully tested, under the optimum conditions, showcasing the excellent functional group tolerance of our photochemical protocol. A series of NMR, UV‐Vis, and DI‐HRMS studies were performed to probe the reaction mechanism.

## 4 Experimental Section

### 4.1 General Procedure for the Photochemical Reaction Between Aldehydes and Substituted Indoles

In a screw‐capped tube containing the corresponding carbonyl derivative (1.0 equiv., 0.20 mmol), indole derivative (2.2 equiv., 0.44 mmol) and benzophenone oxime *p*‐toluene sulfonate (3) (2.5 mol%) were added. After the addition of EtOAc (0.5 mL), the reaction mixture was degassed by bubbling with argon for 5 min. The reaction mixture was left stirring under Kessil lamp irradiation (390 nm) until reaction completion was determined by TLC (3‐5 h). After reaction completion, the reaction mixture was concentrated in vacuo. The desired product was purified by column chromatography.

Additional references cited within the Supporting Information. 1, 2, 3, 4, 5, 6, 7, 8, 9, 10, 11, 12, 13, 14, 15, 16.

## Conflicts of Interest

The authors declare no conflict of interest.

## Supporting information




**Supporting file 1**: chem70514‐sup‐0001‐SuppMat.pdf
