# Supplementary material for: Benzophenone Oxime Tosylate as the Photoacid Generator for the Friedel–Crafts Arylation of Aldehydes With Indoles
Source: Chemistry. 2025 Dec 10;32(2):e03021. doi: 10.1002/chem.202503021 (PMC12790310; doi:10.1002/chem.202503021)
Supplement: Supplementary file 1 — Supporting file 1: chem70514‐sup‐0001‐SuppMat.pdf [file CHEM-32-e03021-s001.pdf]

Benzophenone Oxime Tosylate as the Photoacid Generator for the Friedel-Crafts Arylation of Aldehydes with Indoles

Michael Gκosios,[†] Paraskevi Papatasou,[†] Anastasia Maria Antonaki[†] and Petros L. Gkizis*

Laboratory of Organic Chemistry, Department of Chemistry, Aristotle University of Thessaloniki,
University Campus 54124, Thessaloniki, Greece

SUPPORTING INFORMATION

	**Page**
**General Remarks**	**S3**
**Optimization of the Reaction Conditions for the Friedel-Crafts Arylation of Aldehydes with Indoles**	**S4**
**Control Experiments**	**S8**
**Synthesis of Starting Materials**	**S9**
**General Procedure for the Friedel-Crafts Arylation of Aldehydes with Indoles**	**S12**
**Reaction performed under sunlight**	**S13**
**Gram Scale Reaction**	**S14**
**Mechanistic Studies with NMR**	**S29**
**Mechanistic Studies with UV-Vis**	**S30**
**Mechanistic Studies with DI-HRMS**	**S32**
**References**	**S34**
**NMR Spectra**	**S36**

General Remarks

Chromatographic purification of products was accomplished using forced-flow chromatography on Merck® Kieselgel 60 70-230 mesh. Thin-layer chromatography (TLC) was performed on aluminum backed silica plates (0.2 mm, 60 F_{254}). Visualization of the developed chromatogram was performed by fluorescence quenching using phosphomolybdic acid, anisaldehyde or potassium permanganate stains. Melting points were determined on a Buchi® 530 hot stage apparatus and are uncorrected. Mass spectra (ESI) were recorded on a Finningan® Surveyor MSQ LC-MS spectrometer. HRMS spectra were recorded on a Bruker® Maxis Impact QTOF spectrometer. ^{1}H-NMR and ^{13}C-NMR spectra were recorded on an Ascend™ Bruker 300 MHz (300 MHz and 75 MHz, respectively) or on an Agilent Technologies DD2 500 MHz (500 MHz and 125 MHz, respectively) and are internally referenced to residual solvent signals. Data for ^{1}H-NMR are reported as follows: chemical shift (δ ppm), integration, multiplicity (s = singlet, d = doublet, t = triplet, q = quartet, m = multiplet, br s = broad signal), coupling constant and assignment. Data for ^{13}C-NMR are reported in terms of chemical shift (δ ppm). Mass spectra and conversions of the reactions were recorded on a Shimadzu® GCMS-QP2010 Plus Gas Chromatograph Mass Spectrometer utilizing a MEGA® column (MEGA-5, F.T: 0.25 μm, I.D.: 0.25 mm, L': 30 m, T_{max}: 350 °C, Column ID# 11475). A Varian® Cary 50 UV-Vis spectrophotometer was used for the quantum yield measurements and the UV-Vis data. Kessil lamps PR160L were used as the irradiation source. For all experiments, the intensity of the Kessil lamps was controlled in the maximum level with power consumption: 370 nm (max 43W), 390 nm (max 52W), 427 nm (max 45W), 440 nm (max 45W), 456 nm (max 50W), 467 nm (max 44W) and 525 nm (max 44W).

Optimization of the Reaction Conditions

Irradiation source

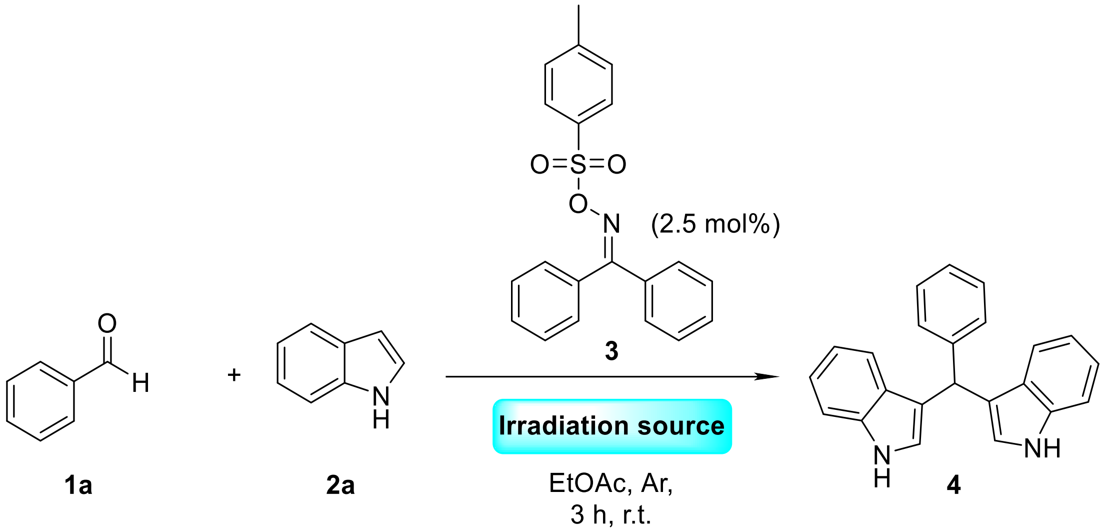

Entry	Irradiation Wavelength (nm)	Yield (%)[a]
1	370	95 (90)
**2**	**390**	**100 (98)**
3	427	92 (88)
4	440	86 (85)
5	456	85 (83)
6	467	70 (63)
7	525	35 (28)

[a] Yield was determined by ^{1}H-NMR, using internal standard. Yield of **4** after purification by column chromatography is presented in parenthesis. The reaction was performed in inert atmosphere with benzaldehyde (**1a**) (21 mg, 0.20 mmol, 1.0 equiv.), indole (**2a**) (51 mg, 0.44 mmol, 2.2 equiv.) and diphenylmethanone *O*-tosyl oxime (**3**) (2 mg, 0.006 mmol, 2.5 mol%) in EtOAc (0.5 mL), under irradiation for 3 h.

Optimization of the Reaction Conditions

Irradiation source

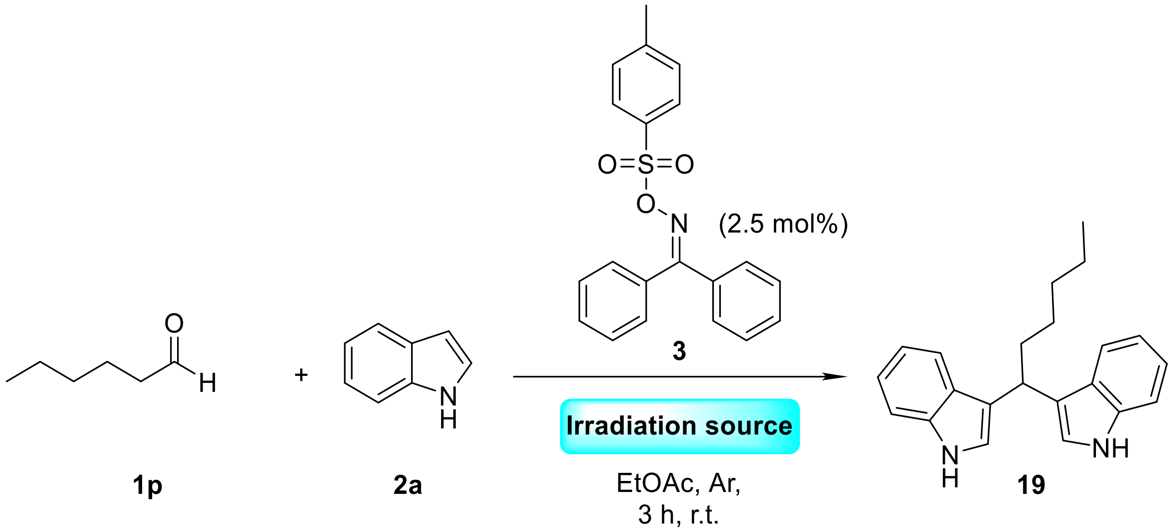

Entry	Irradiation Wavelength (nm)	Yield (%)[a]
1	370	70 (63)
**2**	**390**	**100 (85)**
3	427	85 (80)

[a] Yield was determined by ^{1}H-NMR, using internal standard. Yield of **19** after purification by column chromatography is presented in parenthesis. The reaction was performed in inert atmosphere with hexanal (**1p**) (20 mg, 0.20 mmol, 1.0 equiv.), indole (**2a**) (51 mg, 0.44 mmol, 2.2 equiv.) and diphenylmethanone *O*-tosyl oxime (**3**) (2 mg, 0.006 mmol, 2.5 mol%) in EtOAc (0.5 mL), under irradiation for 3 h.

Solvent Optimization of the Reaction Conditions

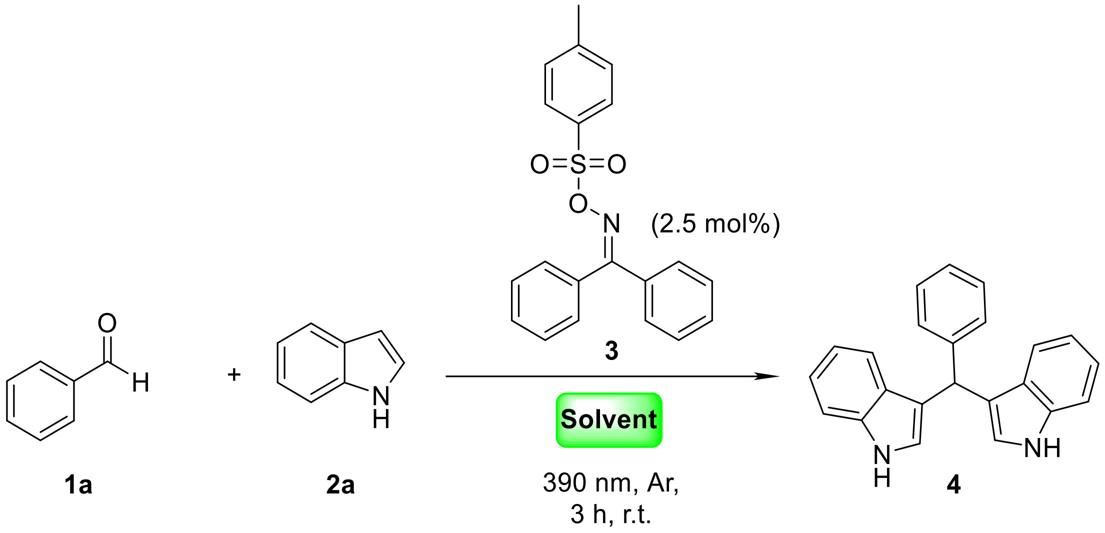

Entry	Solvent	Yield (%)[a]
1	MeCN	57
2	MeOH	81
3	CH_2Cl_2	80
4	$CHCl_3$	73
**5**	**EtOAc**	**98**
6	Pet. Ether	72
7	Toluene	60
8	DMSO-d_6	0
9	DMF	0
10	THF	63
11	Et_2O	66
12	H_2O	70

[a] Yield of **4** after purification by column chromatography. The reaction was performed in inert atmosphere with benzaldehyde (**1a**) (21 mg, 0.20 mmol, 1.0 equiv.), indole (**2a**) (51 mg, 0.44 mmol, 2.2 equiv.) and diphenylmethanone *O*-tosyl oxime (**3**) (2 mg, 0.006 mmol, 2.5 mol%) in solvent (0.5 mL), under UVA LED (Kessil PR160L, 390 nm) irradiation for 3 h.

Optimization of Reaction Concentration

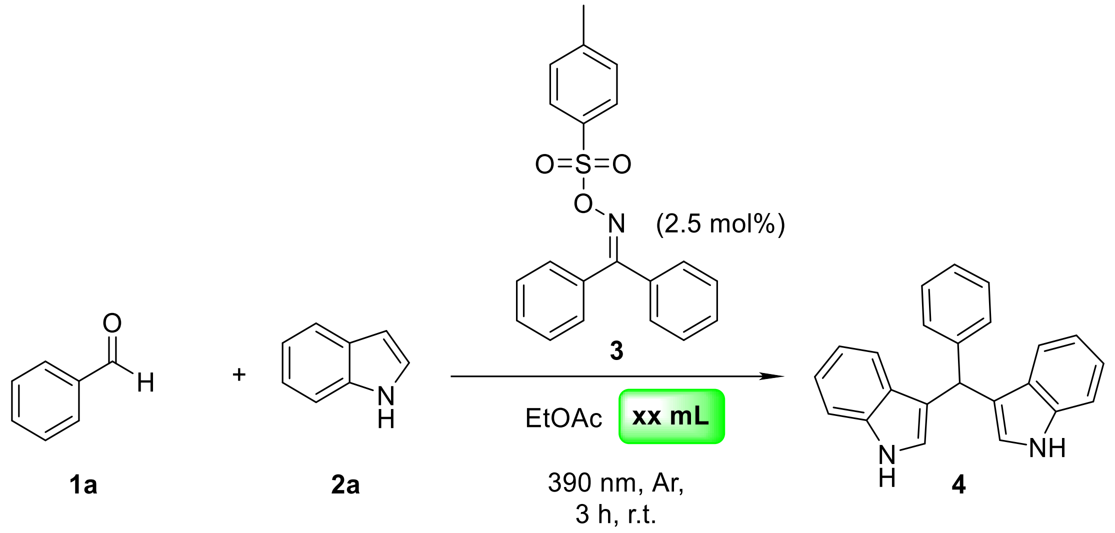

Entry	Solvent Loading (mL)	Yield (%)[a]
1	0.5	98
2	1.0	90
3	2.0	73

[a] Yield of **4** after purification by column chromatography. The reaction was performed in inert atmosphere with benzaldehyde (**1a**) (21 mg, 0.20 mmol, 1.0 equiv.), indole (**2a**) (51 mg, 0.44 mmol, 2.2 equiv.) and diphenylmethanone *O*-tosyl oxime (**3**) (2 mg, 0.006 mmol, 2.5 mol%) in EtOAc (xx mL), under UVA LED (Kessil PR160L, 390 nm) irradiation for 3 h.

Control Experiments

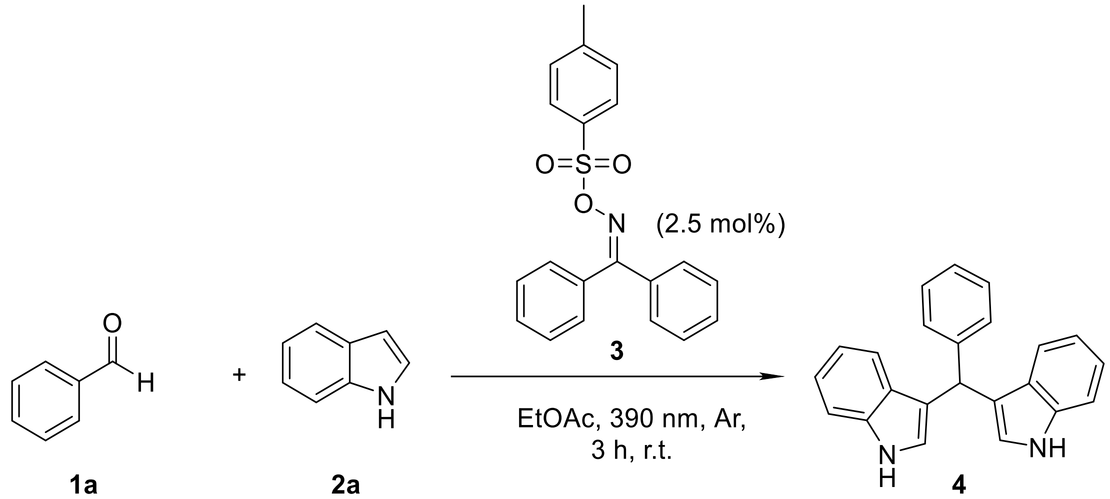

Entry	Variations	Yield (%)[a]
1	No catalyst	12
2	No irradiation (r.t.)	19
3	Under dark	10
4	No irradiation (Heating at 40 °C)	23
5	TEMPO (1.0 equiv.)	0

[a] Yield was determined by ^{1}H-NMR, using internal standard. The reaction was performed in inert atmosphere with benzaldehyde (**1a**) (21 mg, 0.20 mmol, 1.0 equiv.), indole (**2a**) (51 mg, 0.44 mmol, 2.2 equiv.) and diphenylmethanone *O*-tosyl oxime (**3**) (2 mg, 0.006 mmol, 2.5 mol%) in EtOAc (0.5 mL), under UVA LED (Kessil PR160L, 390 nm) irradiation for 3 h.

Synthesis of Starting Materials

Synthesis of Diphenylmethanone *O*-tosyl oxime (3)[1,2]

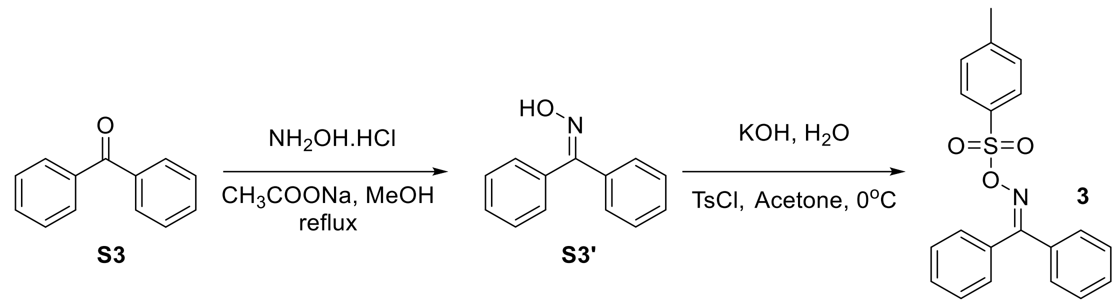

A 50 mL flask was charged with benzophenone (1.0 g, 5.50 mmol), hydroxylamine hydrochloride (570 mg, 8.80 mmol) and sodium acetate (900 mg, 11 mmol) in methanol (10 mL). The reaction mixture was stirred at 65 °C, for 18 h. Then, the reaction mixture was cooled down to room temperature and poured into 30 mL ice/water mixture. White solid was precipitated, filtered and washed with 50 ml precooled water. The precipitated solid was dried under vacuum to afford 1.08 g (**yield: 99 %**) of white solid, which was used in the next step without any purification.[1]

A 25 mL flask was charged with **S3’** (500 mg, 2.53 mmol) in acetone (2 mL). The reaction mixture was cooled down at 0 °C. A solution of KOH (120 mg, 2.14 mmol) in water (2 mL) was added dropwise over a period of 1h, so as the temperature is kept below 10 °C. After the addition completion, a solution of TsCl (361 mg, 1.76 mmol) in acetone (3 mL) is added dropwise, keeping the temperature below 10 °C. After the addition completion, the reaction mixture is stirred at 10-15 °C for 1h. The white precipitate is filtered, washed with a precooled mixture acetone/water (1:1) and dried in a desiccator for 1h to afford 550 mg of compound (**3**).

Yield**: 90%**; white solid; m.p. 90-92 °C; NMR data in accordance with reported literature.[2]

^{1}H NMR ($CDCl_3$, 300 MHz) δ: 7.91 (2H, d, J = 8.3 Hz, ArH), 7.28-7.45 (12H, m, ArH), 2.46 (3H, s, CH_3); **^{13}C NMR** ($CDCl_3$, 75 MHz) δ: 146.6, 137.9, 133.3, 131.9, 130.0, 129.1, 128.8, 128.8, 127.1, 21.8; **MS (ESI)** m/z 352 $[M+H]^+$.

General Procedure for the Synthesis of substituted indole derivatives

1-Methyl-1*H*-indole (2b)[3]

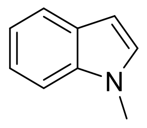

2b

To a stirring solution of indole (352 mg, 3.00 mmol) in dry THF (6 mL) at 0 °C, NaH (180 mg, 60% dispersion in mineral oil, 4.50 mmol) was added under an argon atmosphere. The heterogenous reaction mixture was stirred at 0 °C for 15 min and at room temperature for 1 h. The reaction mixture was then cooled at 0 °C, iodomethane (0.2 ml, 4.00 mmol) was added and allowed to warm at room temperature. After 30 min, the reaction mixture was cooled at 0 °C, quenched with saturated aq. NH_4Cl (5 mL) and extracted with diethyl ether (3 x 50 mL). The combined organic layers were washed with brine (1 x 50 ml), dried over anhydrous Na_2SO_4 and concentrated in vacuo. The resulting oil was purified by flash chromatography (Pet. Ether/AcOEt 10:1); Green oil; Yield: **83%**; NMR data in accordance with reported literature.[3] **^{1}H NMR** ($CDCl_3$, 500 MHz) δ: 7.68 (1H, d, J = 7.9 Hz, ArH), 7.38 (1H, d, J = 7.9 Hz, ArH), 7.28 (1H, t, J = 7.9 Hz, ArH), 7.16 (1H, t, J = 7.9 Hz, ArH), 7.10 (1H, d, J = 2.5 Hz, ArH), 6.54 (1H, d, J = 2.5 Hz, ArH), 3.84 (3H, s, NCH_3); **^{13}C NMR** ($CDCl_3$, 125 MHz) δ: 136.7, 128.7, 128.4, 121.4, 120.8, 119.2, 109.1, 100.9, 32.8; **MS (ESI)** m/z 154 $[M+Na]^+$.

1-Benzyl-1*H*-indole (2c)[3]

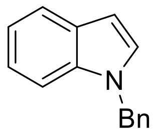

2c

Same procedure as above using benzyl bromide; Yellow solid; m.p.: 38-41°C; Yield: **82%**; NMR data in accordance with reported literature.[3]

^{1}H NMR ($CDCl_3$, 500 MHz) δ: 7.69 (1H, d, J = 7.8 Hz, ArH), 7.32 – 7.28 (4H, m, ArH), 7.17-7.11 (5H, m, ArH), 6.59 (1H, d, J = 2.3 Hz, ArH), 5.36 (2H, s, NCH_2); **^{13}C NMR** ($CDCl_3$, 125 MHz) δ: 137.5, 136.3, 128.7, 128.6, 128.2, 127.6, 126.8, 121.7, 121.0, 119.5, 109.7, 101.7, 50.1; **MS (ESI)** m/z 230 $[M+Na]^+$.

1-(Prop-2-yn-1-yl)-1*H*-indole (2d)[4]

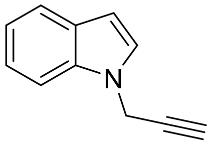

2d

Same procedure as above using propargyl bromide; Colorless oil; Yield: **76%**; NMR data in accordance with reported literature.[4]

^{1}H NMR ($CDCl_3$, 300 MHz) δ: 7.72 (1H, d, J = 7.8 Hz, ArH), 7.46 (1H, d, J = 8.3 Hz, ArH), 7.33 (1H t, J = 8.2 Hz, ArH), 7.28 – 7.19 (2H, m, ArH), 6.61 (1H, d, J = 4.0 Hz, ArH) 4.89 (2H, d, J = 2.6 Hz, NCH_2), 2.45 (1H, t, J = 2.5 Hz, CH); **^{13}C NMR** ($CDCl_3$, 75 MHz) δ: 135.85, 128.96, 127.32, 121.96, 121.20, 119.96, 109.41, 102.16, 77.84, 73.60, 35.82; **MS (ESI)** m/z 156 $[M+Na]^+$.

General Procedure for the Reaction of Aldehydes with Indoles

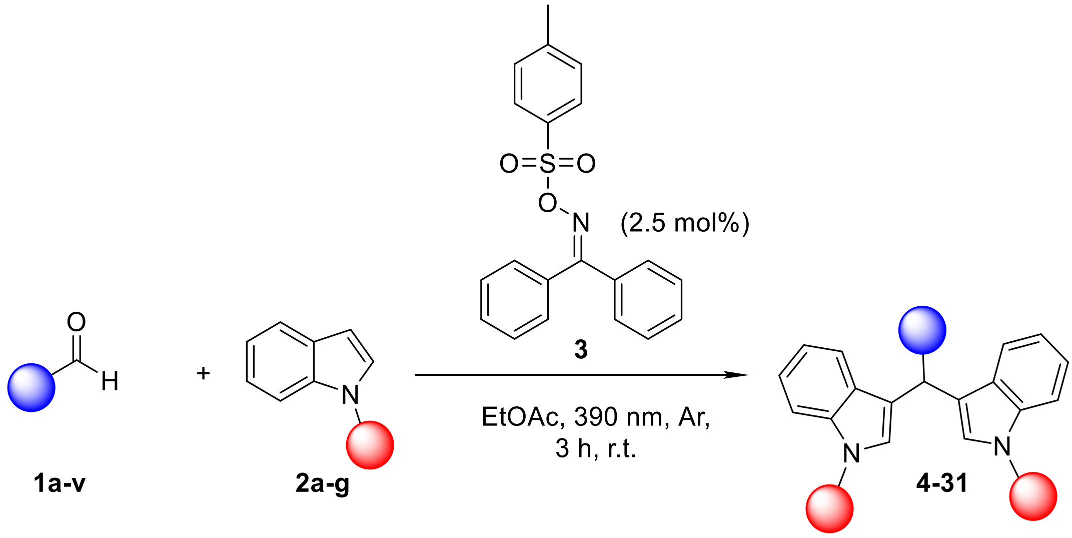

In a screw capped tube containing the corresponding carbonyl derivative (**1a-v**) (1.0 equiv., 0.20 mmol), indole (**2a-g**) (2.2 equiv., 0.44 mmol) and diphenylmethanone *O*-tosyl oxime (**3**) (2 mg, 2.5 mol%) were added. After the addition of EtOAc (0.5 mL) the reaction mixture was degassed by bubbling with argon for 5 minutes. The reaction mixture was left stirring under Kessil lamp irradiation (390 nm) until reaction completion was determined by TLC (3-5 h). After reaction completion, the reaction mixture was concentrated *in vacuo*. The desired product was purified by column chromatography.

A **B** **C**

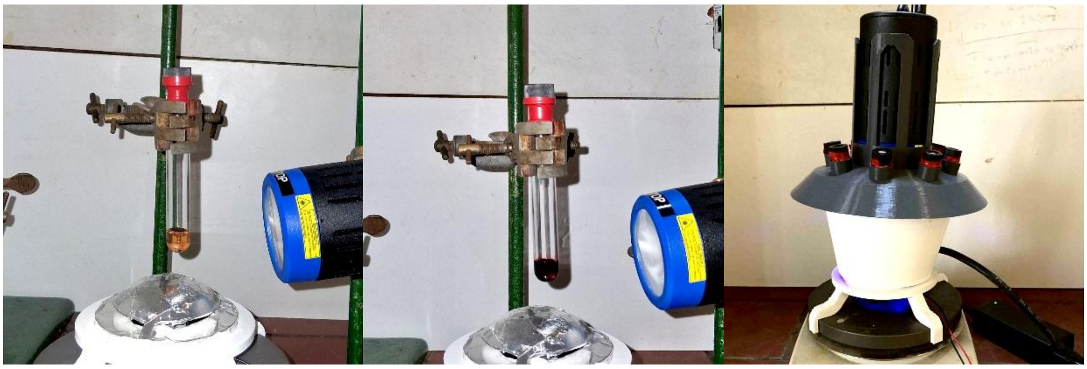

A. Reaction mixture before the reaction setup; **B.** Reaction mixture after reaction completion. **C.** Reaction employing the batch reaction technology introduced by Noel group.[5]

Reaction of Benzaldehyde (1a) with Indole (2a) under sunlight

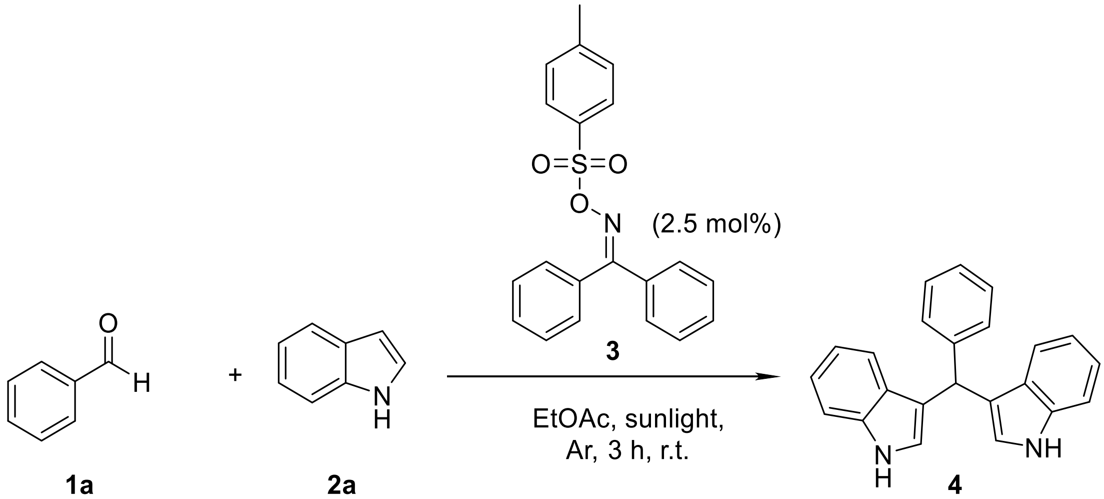

1a **2a** **4**

In a screw capped tube containing the corresponding benzaldehyde (**1a**) (42 mg. 1.0 equiv., 0.40 mmol), indole (**2a**) (102 mg, 2.2 equiv., 0.88 mmol) and diphenylmethanone O-tosyl oxime (**3**) (4 mg, 2.5 mol%) were added. After the addition of EtOAc (1.0 mL) the reaction mixture was degassed by bubbling with argon for 5 minutes. The reaction mixture was left stirring sunlight until reaction completion was determined by TLC (6 h). After reaction completion, the reaction mixture was concentrated *in vacuo*. The desired product was purified by column chromatography, using as eluent a mixture of petroleum ether/ethyl acetate (9:1), to afford 116 mg of compound **4**. Yield: **90%**.

A **B**

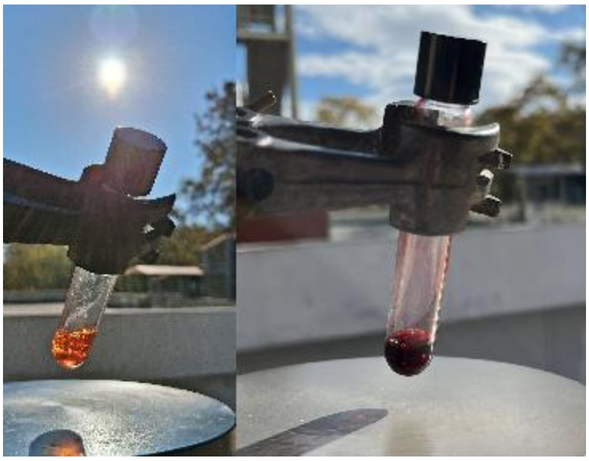

A. Reaction mixture under sunlight irradiation; **B.** Reaction mixture after reaction completion.

Gram Scale Reaction of Benzaldehyde (1a) with Indole (2a)

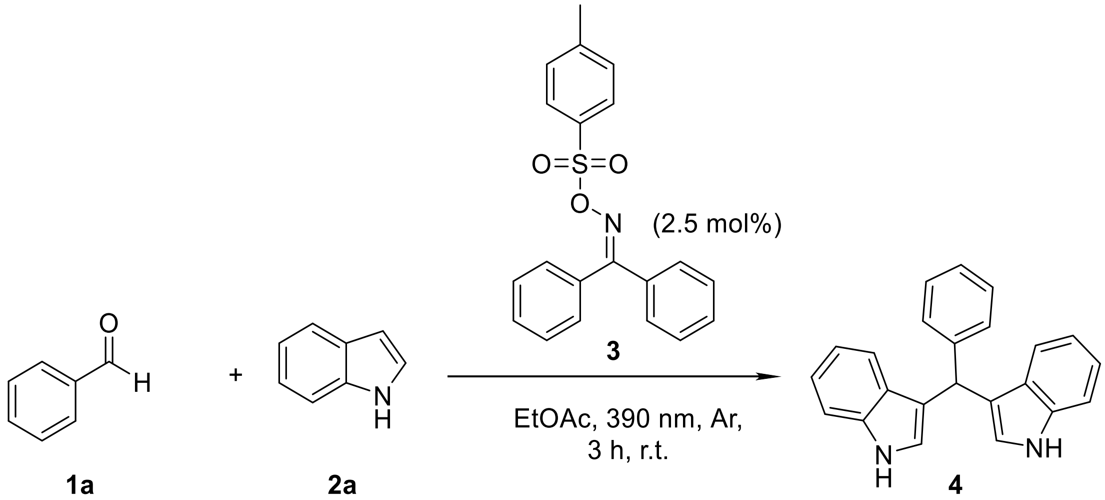

In a screw capped tube containing the corresponding benzaldehyde (**1a**) (466 mg. 1.0 equiv., 4.40 mmol), indole (**2a**) (1.14 g, 2.2 equiv., 9.70 mmol) and diphenylmethanone O-tosyl oxime (**3**) (40 mg, 2.5 mol%) were added. After the addition of EtOAc (20 mL) the reaction mixture was degassed by bubbling with argon for 5 minutes. The reaction mixture was left stirring under Kessil lamp irradiation (390 nm) until reaction completion was determined by TLC (18 h). After reaction completion, the reaction mixture was concentrated *in vacuo*. The desired product was purified by column chromatography, using as eluent a mixture of petroleum ether/ethyl acetate (9:1), to afford 1.34 g of compound **4**. Yield: **94%**.

A **B** **C**

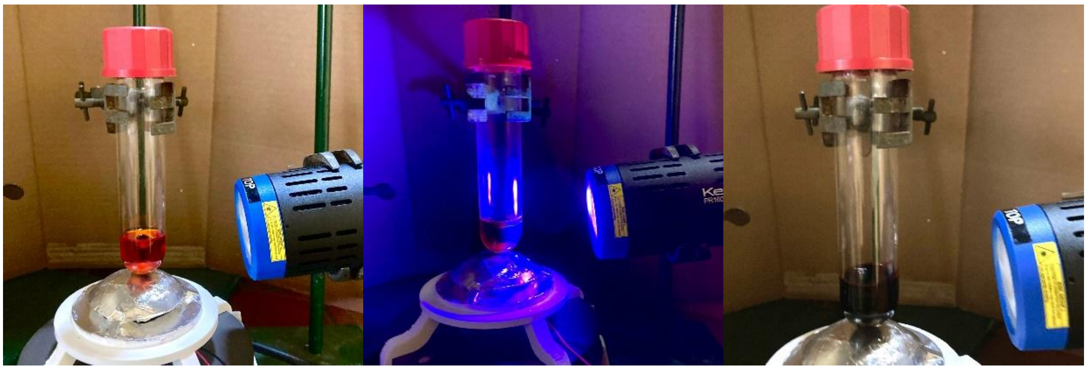

A. Reaction mixture before the reaction setup; **B.** Reaction mixture under UVA LED (Kessil PR160L, 390 nm) irradiation; **C.** Reaction mixture after reaction completion.

3,3′-(Phenylmethylene)bis(1*H*-Indole) (4)[3]

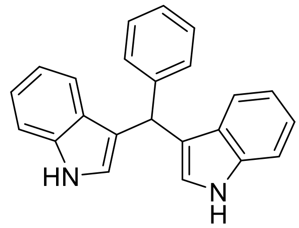

Reaction time: **3 h**; Yield**: 98%**; Red foam; Eluent: Petroleum ether / Ethyl acetate 9:1; NMR data in accordance with reported literature.[3]

^{1}H NMR ($CDCl_3$, 300 MHz) δ: 3 7.74 (2H, br s, 2 x NH), 7.43 (2H, d, J = 7.5 Hz, ArH), 7.38 (2H, d, J = 7.5 Hz, ArH), 7.34-7.31 (4H, m, ArH), 7.28-7.24 (1H, m, ArH), 7.20 (2H, t, J = 7.5 Hz, ArH), 7.04 (2H, t, J = 7.5 Hz, ArH), 6.58 (2H, d, J = 1.6 Hz, ArH), 5.91 (1H, s, CH); **^{13}C NMR** ($CDCl_3$, 75 MHz) δ: 144.2, 136.8, 128.8, 128.3, 127.2, 126.2, 123.8, 122.0, 120.0, 119.7, 119.3, 111.2, 40.3; **MS (ESI)** m/z 345 $[M+H]^+$.

3,3′-(*p*-Tolylmethylene)bis(1*H*-Indole) (5)[6]

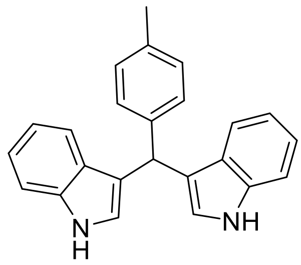

5

Reaction time: **3 h**; Yield**: 93%**; Brown solid; m.p.: 95-97 °C; Eluent: Petroleum ether / Ethyl acetate 9:1; NMR data in accordance with reported literature.[6]

^{1}H NMR ($CDCl_3$, 300 MHz) δ: 7.79 (2H, br s, 2 x NH), 7.43 (2H, d, J = 8.2 Hz, ArH), 7.33 (2H, d, J = 8.2 Hz, ArH), 7.26 (2H, d, J = 8.2 Hz, ArH), 7.22–7.15 (2H, m, ArH), 7.11 (2H, d, J = 7.0 Hz, ArH), 7.03 (2H, ddd, J = 8.2, 7.0, 1.3 Hz, ArH), 6.61 (2H, d, J = 1.3 Hz, ArH), 5.87 (1H, s, CH), 2.36 (3H, s, CH_3); **^{13}C NMR** ($CDCl_3$, 75 MHz) δ: 141.1, 136.8, 135.6, 129.0, 128.7, 127.2, 123.7, 122.0, 120.1, 119.9, 119.3, 111.2, 39.9, 21.2; **MS (ESI)** m/z 337 $[M+H]^+$.

3,3′-((4-Methoxyphenyl)methylene)bis(1*H*-Indole) (6)[3]

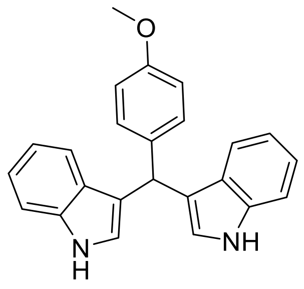

6

Reaction time: **3 h**; Yield**: 91%**; Orange solid; m.p.: 76-78 ºC; Eluent: Petroleum ether / Ethyl acetate 9:1; NMR data in accordance with reported literature.[3]

^{1}H NMR ($CDCl_3$, 300 MHz) δ: 7.89 (2H, br s, 2 x NH), 7.39 (2H, d, J = 7.9 Hz, ArH), 7.33 (2H, d, J = 7.9 Hz, ArH), 7.30-7.21 (2H, m, ArH), 7.16 (2H, t, J = 8.2 Hz, ArH), 7.04 (2H, t, J = 8.2 Hz, ArH), 6.85 (2H, d, J = 8.8 Hz, ArH), 6.62 (2H, d, J = 1.2 Hz, ArH), 5.83 (1H, s, CH), 3.78 (3H, s, OCH_3); **^{13}C NMR** ($CDCl_3$, 75 MHz) δ: 158.0, 136.9, 136.4, 129.7, 127.2, 123.7, 122.0, 120.2, 120.1, 119.3, 113.7, 111.2, 55.3, 39.5; **MS (ESI)** m/z 375 $[M+H]^+$.

3,3′-((4-Fluorophenyl)methylene)bis(1*H*-Indole) (7)[6]

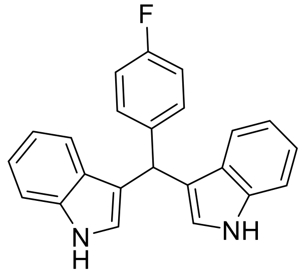

7

Reaction time: **3 h**; Yield**: 91%**; Brown solid; m.p.: 95-98 ºC; Eluent: Petroleum ether / Ethyl acetate 9:1; NMR data in accordance with reported literature.[6]

^{1}H NMR ($CDCl_3$, 500 MHz) δ: 7.87 (2H, br s, 2 x NH), 7.39 (2H, d, J = 7.6 Hz, ArH), 7.35 (2H, d, J = 8.5 Hz, ArH), 7.31 – 7.25 (2H, m, ArH), 7.19 (2H, t, J = 7.6 Hz, ArH), 7.03 (2H, t, J = 7.6 Hz, ArH), 6.97 (2H, t, J = 8.5 Hz, ArH), 6.61 (2H, s, ArH), 5.88 (1H, s, CH); **^{13}C NMR** ($CDCl_3$, 125 MHz) δ: 161.5 (d, J = 243.7 Hz), 139.8 (d, J = 2.9 Hz), 136.8, 130.2 (d, J = 7.7 Hz), 127.0, 123.7, 122.1, 120.0, 119.6, 119.4, 115.01 (d, J = 21.3 Hz), 111.2, 39.6; **MS (ESI)** m/z 341 $[M+H]^+$.

3,3′-((4-Chlorophenyl)methylene)bis(1*H*-Indole) (8)[3]

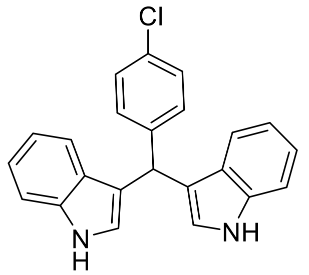

8

Reaction time: **3 h**; Yield**: 82%**; Orange foam; Eluent: Petroleum ether / Ethyl acetate 9:1; NMR data in accordance with reported literature.[3]

^{1}H NMR ($CDCl_3$, 300 MHz) *δ*: ^{1}H NMR (500 MHz, $CDCl_3$) *δ:* 7.68 (2H, br s, 2 x NH), 7.44 (2H, d, *J* = 8.0 Hz, ArH), 7.33 (2H, d, *J* = 8.0 Hz, ArH), 7.28 (4H, s, ArH), 7.27-7.23 (2H, m, ArH), 7.12-7.08 (2H, m, ArH), 6.54 (2H, dd, *J* = 2.4 and 0.8 Hz, ArH), 5.90 (1H, s, CH); **^{13}C NMR** ($CDCl_3$, 125 MHz) *δ*: 142.5, 136.6, 131.7, 130.0, 128.3, 126.8, 123.6, 122.0, 119.8, 119.3, 119.0, 111.1, 39.5; **MS (ESI)** m/z 379 $[M+Na]^+$.

3,3′-((4-Bromophenyl)methylene)bis(1*H*-Indole) (9)[3]

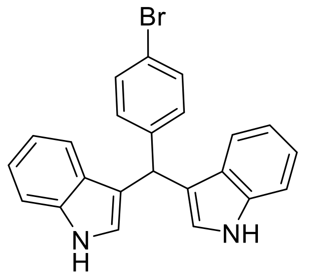

9

Reaction time: **3 h**; Yield**: 60%**; Red foam; Eluent: Petroleum ether / Ethyl acetate 9:1; NMR data in accordance with reported literature.[3]

^{1}H NMR ($CDCl_3$, 300 MHz) *δ*: ^{1}H NMR (300 MHz, $CDCl_3$) *δ:* 7.86 (2H, br s, 2 x NH), 7.41 – 7.34 (6H, m, ArH), 7.21 – 7.17 (4H, m, ArH), 7.04 (2H, t, *J* = 8.0 Hz, ArH), 6.58 (2H, s, ArH), 5.85 (1H, s, CH); **^{13}C NMR** ($CDCl_3$, 75 MHz) *δ*: 143.2, 136.8, 131.4, 130.6, 127.0, 123.7, 122.2, 120.0, 119.9, 119.5, 119.2, 111.3, 39.8; **MS (ESI)** m/z 425 $[M+Na]^+$.

4-(di(1*H*-indol-3-yl)methyl)benzoic acid (10)[7]

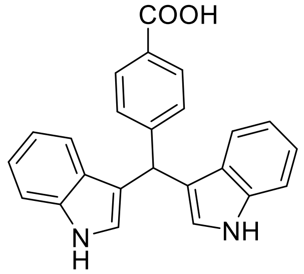

10

Reaction time: **3 h**; Yield: **53%**; Amorphous solid; m.p.: 208-210 °C; Eluent: Petroleum ether / Ethyl acetate 4:1; NMR data in accordance with reported literature.[7]

^{1}H NMR (CD_3OD, 500 MHz) δ: 7.92 (2H, d, J = 8.4 Hz, ArH), 7.40 (2H, d, J = 8.4 Hz, ArH), 7.33 (2H, d, J = 8.4 Hz, ArH), 7.25 (2H, d, J = 8.0 Hz, ArH), 7.05 (2H, t, J = 8.0 Hz, ArH), 6.87 (2H, t, J = 8.0 Hz, ArH), 6.66 (2H, s, ArH), 5.90 (1H, s, CH); **^{13}C NMR** (CD_3OD, 125 MHz) δ: 170.1, 152.0, 138.4, 130.6, 129.9, 129.4, 128.2, 124.8, 122.3, 120.3, 119.5, 119.2, 112.3, 41.7; **MS (ESI)** m/z 367 $[M+H]^+$.

3,3′-((3-Nitrophenyl)methylene)bis(1*H*-Indole) (11)[8]

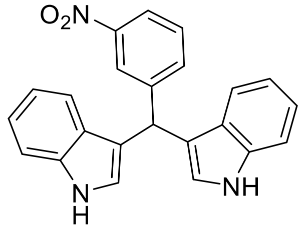

11

Reaction time: **3 h**; Yield: **59%**; Red solid; m.p.: 259-261 °C; Eluent: Petroleum ether / Ethyl acetate 8:1; NMR data in accordance with reported literature.[8]

^{1}H NMR ($CDCl_3$, 300 MHz) δ: 8.21 (1H, s, ArH), 8.08 (1H, d, J = 7.7 Hz, ArH), 8.00 (2H, br s, 2 x NH), 7.69 (1H, d, J = 7.7 Hz, ArH), 7.44 (1H, t, J = 7.9 Hz, ArH), 7.37 (4H, m, ArH), 7.20 (2H, t, J = 7.6 Hz, ArH), 7.03 (2H, t, J = 7.6 Hz, ArH), 6.66 (2H, s, ArH), 6.00 (1H, s, CH); **^{13}C NMR** ($CDCl_3$, 75 MHz) δ: 148.6, 146.5, 136.9, 135.0, 129.3, 126.8, 123.8, 123.7, 122.4, 121.6, 119.7, 119.7, 118.4, 111.4, 40.1; **MS (ESI)** 368 $[M+H]^+$.

2-(Di(1*H*-Indol-3-yl)methyl)-4-nitrophenol (12)[9]

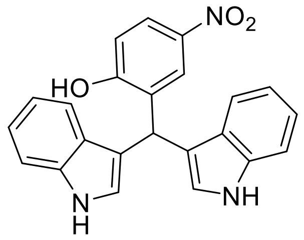

12

Reaction time: **3 h**; Yield**: 69%**; Orange solid; m.p.: 249-251 °C; Eluent: Petroleum ether / Ethyl acetate 5:1; NMR data in accordance with reported literature.[9]

^{1}H NMR ($CDCl_3$, 300 MHz) δ: 8.11 – 8.01 (4H, m, 2 x NH and 2x ArH), 7.39 – 7.35 (4H, m, ArH), 7.22 (2H, t, J = 7.5 Hz, ArH), 7.06 (2H, t, J = 7.5 Hz, ArH), 6.88 (1H, d, J = 8.9 Hz, ArH), 6.69 (2H, s, ArH), 6.47 (1H, s, OH), 6.08 (1H, s, CH); **^{13}C NMR** ($CDCl_3$, 75 MHz) δ: 160.4, 141.8, 137.0, 130.4, 126.5, 126.2, 124.5, 123.8, 122.8, 120.0, 119.5, 117.0, 116.0, 111.6, 35.5; **MS (ESI)** m/z 410 $[M-H]^-$.

5-Bromo-2-(di(1*H*-Indol-3-yl)methyl)phenol (13)[10]

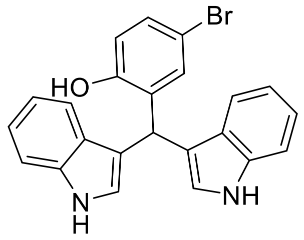

13

Reaction time: **3 h**; Yield: **66%**; Pink solid; m.p.: 138-140 °C; Eluent: Petroleum ether / Ethyl acetate 9:1; NMR data in accordance with reported literature.[10]

^{1}H NMR ($CDCl_3$, 500 MHz) δ: 7.97 (2H, br s, 2 x NH), 7.38–7.36 (4H, m, ArH), 7.28–7.22 (2H, m, ArH), 7.19 (2H, t, J = 7.6 Hz, ArH), 7.03 (2H, t, J = 7.5 Hz, ArH), 6.72–6.71 (3H, m, ArH), 5.94 (1H, s, CH) 5.44 (1H, s, OH); **^{13}C NMR** ($CDCl_3$, 125 MHz) δ: 153.9, 137.0, 132.6, 131.6, 131.0, 126.8, 123.8, 122.7, 119.9, 119.8, 118.7, 116.6, 113.1, 111.4, 35.9; **MS (ESI)** m/z 417 $[M+H]^+$.

3-(Di(1*H*-Indol-3-yl)methyl)benzene-1,2-diol (14)[11]

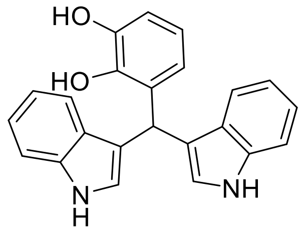

14

Reaction time: **3 h**; Yield**: 82%**; Red foam; Eluent: Petroleum ether / Ethyl acetate 5:1; NMR data in accordance with reported literature.[11]

^{1}H NMR ($CDCl_3$, 300 MHz) *δ* 7.97 (2H, br s, 2 x NH), 7.43–7.36 (4H, m, ArH), 7.24-7.18 (3H, m, ArH), 7.04 (2H, t, *J* = 7.5 Hz, ArH), 6.87–6.81 (2H, m, ArH),6.79-6.76 (3H, m, 2 x ArH and OH), 5.98 (1H, s, CH), 5.44 (1H, s, OH); **^{13}C NMR** ($CDCl_3$, 75 MHz) *δ*: 145.1, 141.8, 137.0, 129.8, 126.9, 123.7, 122.6, 121.5, 121.2, 120.1, 119.8, 117.2, 113.8, 111.4, 36.3; **MS (ESI)** m/z 355 $[M+H]^+$.

3,3′-Benzo[*d*][1,3]dioxol-5-ylmethylene)bis(1*H*-Indole) (15)[12]

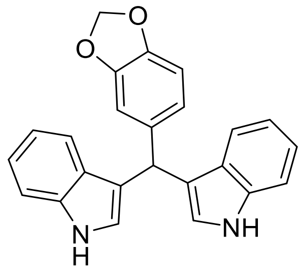

15

Reaction time: **3 h**; Yield**: 82%**; Brown solid; m.p.: 162-164 °C; Eluent: Petroleum ether / Ethyl acetate 9:1; NMR data in accordance with reported literature.[12]

^{1}H NMR ($CDCl_3$, 300 MHz) *δ*: 7.89 (2H, br s, 2 x NH), 7.42 (2H, d, *J* = 7.9 Hz, ArH), 7.33 (2H, d, *J* = 7.9 Hz, ArH), 7.18 (2H, t, *J* = 8.0 Hz, ArH), 7.03 (2H, t, *J* = 8.0 Hz, ArH), 6.92 – 6.80 (2H, m, ArH), 6.73 (1H, d, *J* = 8.0 Hz, ArH), 6.62 (2H, s, ArH), 5.90 (2H, s, CH_2), 5.81 (s, 1H, CH); **^{13}C NMR** ($CDCl_3$, 75 MHz) *δ*: 147.6, 145.9, 138.3, 136.8, 127.1, 123.6, 122.0, 121.7, 120.0, 119.8, 119.3, 111.2, 109.4, 108.1, 100.9, 40.0; **MS (ESI)** m/z 367 $[M+H]^+$.

3,3′-(Naphthalen-1-ylmethylene)bis(1*H*-Indole) (16)[13]

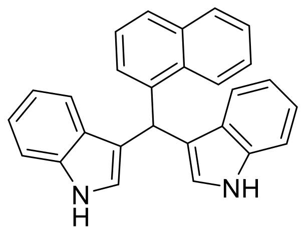

16

Reaction time: **3 h**; Yield: **83%**; Red solid; m.p.: 248-250 °C; Eluent: Petroleum ether / Ethyl acetate 9:1; NMR data in accordance with reported literature.[13]

^{1}H NMR (DMSO, 500 MHz) δ: 10.79 (2H, br s, 2 x NH), 8.26 (1H, d, J = 8.3 Hz, ArH), 7.92 (1H, d, J = 7.8 Hz, ArH), 7.77 (1H, d, J = 8.1 Hz, ArH), 7.49 – 7.41 (2H, m, ArH), 7.39 – 7.33 (3H, m, ArH), 7.27 (3H, d, J = 8.7 Hz, ArH), 7.04 (2H, t, J = 7.8 Hz, ArH), 6.85 (2H, t, J = 7.5 Hz, ArH), 6.76 – 6.72 (2H, m, ArH), 6.63 (1H, s, CH); **^{13}C NMR** (DMSO, 125 MHz) δ: 140.3, 136.7, 133.6, 131.3, 128.6, 126.6, 126.6, 125.8, 125.5, 125.3, 125.3, 124.3, 124.0, 120.9, 119.0, 118.3, 117.7, 111.5, 35.4; **MS (ESI)** m/z 395 $[M+Na]^{+}$.

3,3′-(Ethane-1,1-diyl)bis(1*H*-Indole) (17)[14]

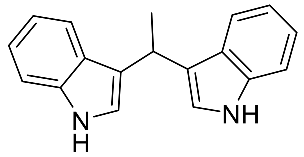

17

Reaction time: **3 h**; Yield**: 28%**; Yellow solid; m.p.: 148-150 °C; Eluent: Petroleum ether / Ethyl acetate 9:1; NMR data in accordance with reported literature.[14]

^{1}H NMR ($CDCl_3$, 300 MHz) δ: 7.89 (2H, br s, 2 x NH), 7.58 (2H, d, J = 8.0 Hz, ArH), 7.35 (2H, d, J = 8.0 Hz, ArH), 7.17 (2H, td, J = 7.6, 1.2 Hz, ArH), 7.04 (2H, td, J = 7.6, 1.1 Hz, ArH), 6.93 (2H, s, ArH), 4.69 (1H, q, J = 7.1 Hz, CH) 1.82 (3H, d, J = 7.1 Hz, CH_3); **^{13}C NMR** ($CDCl_3$, 75 MHz) δ: 136.8, 127.1, 121.9, 121.9, 121.3, 119.9, 119.2, 111.2, 28.3, 21.9; **MS (ESI)** m/z 283 $[M+Na]^{+}$.

3,3′-(2-Methylpropane-1,1-diyl)bis(1*H*-Indole) (18)[15]

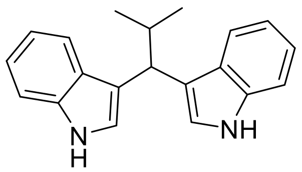

18

Reaction time: **3 h**; Yield: **24%**; White foam; Eluent: Petroleum ether / Ethyl acetate 5:1; NMR data in accordance with reported literature.[15]

^{1}H NMR ($CDCl_3$, 300 MHz) δ: 7.86 (2H, br s, 2 x NH), 7.65 (2H, d, J = 8.9 Hz, ArH), 7.30 (2H, d, J = 8.0 Hz, ArH), 7.18 – 7.11 (2H, m, ArH), 7.08 – 7.03 (4H, m, ArH), 4.27 (1H, d, J = 8.4 Hz, CH), 2.65 (1H, m, CH), 1.03 (6H, d, J = 6.1 Hz, 2 x CH_3); **^{13}C NMR** ($CDCl_3$, 75 MHz) δ: 136.4, 127.9, 121.8, 121.8, 119.9, 119.8, 119.1, 111.1, 41.2, 33.0, 22.0; **MS (ESI)** m/z 289 $[M+H]^+$.

3,3′-(Hexane-1,1-diyl)bis(1*H*-Indole) (19)[12]

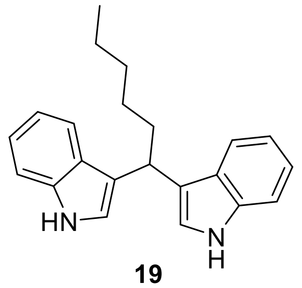

19

Reaction time: **3 h**; Yield**: 85%**; Brown solid; m.p.: 60-62 °C; Eluent: Petroleum ether / Ethyl acetate 9:1; NMR data in accordance with reported literature.[12]

^{1}H NMR ($CDCl_3$, 300 MHz) δ: 7.78 (2H, br s, 2 x NH), 7.65 (2H, d, J = 8.9 Hz, ArH), 7.32 (2H, d, J = 8.1 Hz, ArH), 7.19 (2H, t, J = 7.5 Hz, ArH), 7.09 (2H, t, J = 7.5 Hz, ArH), 6.95 (2H, s, ArH), 4.51 (1H, t, J = 7.4 Hz, CH), 2.33–2.17 (2H, m, CH_2), 1.55 – 1.23 (6H, m, 3 x CH_2), 1.01–0.81 (3H, m, CH_3); **^{13}C NMR** ($CDCl_3$, 75 MHz) δ: 136.6, 127.2, 121.7, 121.5, 120.6, 119.7, 119.0, 111.1, 35.9, 34.1, 32.1, 28.1, 22.7, 14.2; **MS (ESI)** m/z 317 $[M+H]^+$.

3,3´-(Octane-1,1-diyl)bis(1*H*-Indole) (20)[6]

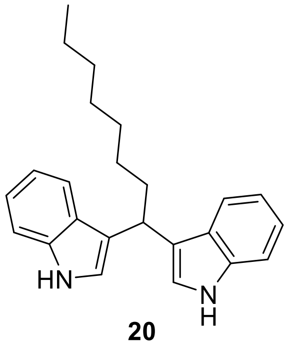

20

Reaction time: **3 h**; Yield: **80%**; Brown solid; m.p.: 117-119 °C; Eluent: Petroleum ether / Ethyl acetate 9:1; NMR data in accordance with reported literature.[6]

^{1}H NMR ($CDCl_3$, 300 MHz) δ: 7.88 (2H, br s, 2 x NH), 7.62 (2H, d, J = 7.9 Hz, ArH), 7.33 (2H, d, J = 8.2 Hz, ArH), 7.16 (2H, t, J = 7.6 Hz, ArH), 7.05 (2H, t, J = 7.6 Hz, ArH), 6.98 (2H, s, ArH), 4.49 (1H, t, J = 7.4 Hz, CH), 2.37-2.19 (6H, m, 2H, 3 x CH_2), 1.28−1.26 (6H, m, 3 x CH_2), 0.89 (3H, t, J = 7.0 Hz, CH_3); **^{13}C NMR** ($CDCl_3$, 75 MHz) δ: 136.7, 127.3, 121.8, 121.5, 120.7, 119.8, 119.1, 111.2, 36.0, 34.1, 32.1, 29.9, 29.4, 28.5, 22.8, 14.2; **MS (ESI)** m/z 345 $[M+H]^+$.

3,3´-(Cyclobutylmethylene)bis(1*H*-Indole) (21)

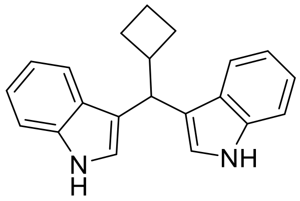

21

Reaction time: **3 h**; Yield: **75%**; Brown oil; Eluent: Petroleum ether / Ethyl acetate 9:1.

^{1}H NMR ($CDCl_3$, 300 MHz) δ: δ 7.83 (2H, br s, 2 x NH), 7.59 (2H, d, J = 7.9 Hz, ArH), 7.26 (2H, d, J = 7.9 Hz, ArH), 7.14 (2H, t, J = 7.6 Hz, ArH), 7.03 (2H, t, J = 7.6 Hz, 1H), 6.96 (2H, s, 2H), 4.46 (1H, d, J = 10.1 Hz, CH), 3.27–3.19 (1H, m, CH), 2.12–2.08 (2H, m, CH_2), 1.97–1.86 (4H, m, 2 x CH_2).

^{13}C NMR ($CDCl_3$, 75 MHz) δ: 136.5, 127.6, 121.6, 121.5, 119.7, 119.0, 118.9, 111.1, 41.2, 40.5, 28.2, 18.0; **MS (ESI)** m/z 301 $[M+H]^+$.

3,3´-(3-Phenylpropane-1,1-diyl)bis(1*H*-Indole) (22)[3]

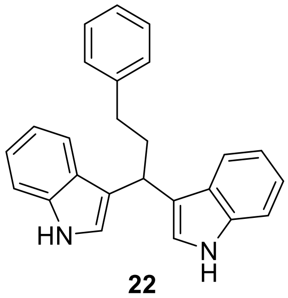

22

Reaction time: **3 h**; Yield: **89%**; Brown solid; m.p.: 155-157 °C; Eluent: Petroleum ether / Ethyl acetate 9:1; NMR data in accordance with reported literature.[3]

^{1}H NMR ($CDCl_3$, 300 MHz) δ: 7.85 (2H, br s, 2 x NH), 7.56 (2H, d, J = 8.1 Hz, ArH), 7.34–7.26 (4H, m, ArH), 7.19–7.14 (5H, m, ArH), 7.06 –7.04 (2H, m, ArH), 7.00 (2H, s, ArH), 4.52 (1H, t, J = 7.4 Hz, CH), 2.74 – 2.71 (2H, m, CH_2), 2.57 – 2.55 (2H, m, CH_2); **^{13}C NMR** ($CDCl_3$, 75 MHz) δ: 142.8, 136.8, 128.7, 128.4, 127.2, 125.8, 121.9, 121.6, 120.2, 119.8, 119.2, 111.2, 37.5, 34.6, 33.6; **MS (ESI)** m/z 373 $[M+H]^+$.

3,3´-(Cyclohexane-1,1-diyl)bis(1*H*-Indole) (23)[3]

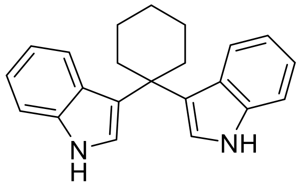

23

Reaction time: **3 h**; Yield: **78%**; Brown foam; Eluent: Petroleum ether / Ethyl acetate 9:1; NMR data in accordance with reported literature.[3]

^{1}H NMR ($CDCl_3$, 500 MHz) δ: 7.78 (2H, br s, 2 x NH), 7.63 (2H, d, J = 7.6 Hz, ArH), 7.29 (2H, d, J = 7.6 Hz, ArH), 7.12 (2H, t, J = 7.6 Hz, ArH), 7.03 (2H, s, ArH), 6.97 (2H, t, J = 7.6 Hz, ArH), 2.61-2.58 (4H, m, 4 x CH*H*), 1.73-1.62 (6H, m, 6 x CH*H*); **^{13}C NMR** ($CDCl_3$, 125 MHz) δ: 137.0, 126.3, 123.6, 122.1, 121.4, 121.2, 118.5, 111.1, 39.5, 36.8, 26.8, 23.0; **MS (ESI)** m/z 337 $[M+Na]^+$.

3,3′-(3-Methylcyclohexane-1,1-diyl)bis(1*H*-Indole) (24)

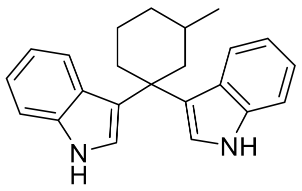

24

Reaction time: **3 h**; Yield: **67%**; mixture of conformers; Pink oil; Eluent: Petroleum ether / Ethyl acetate 10:1.

^{1}H NMR ($CDCl_3$, 300 MHz) δ: 7.96 (2H, br s, 2 x NH), 7.65 (2H, d, J = 7.6 Hz, ArH), 7.53 (2H, d, J = 8.0 Hz, ArH), 7.34-7.24 (3H, m, ArH), 7.14-7.06 (2H, m, ArH), 6.99-6.90 (2H, m, ArH), 6.81 (1H, s, ArH), 2.86-2.85 (1H, m, 2 x CH*H*), 2.25-2.14 (1H, m, CH), 1.87-1.73 (4H, m, 4 x CH*H*), 1.17-1.12 (2H, m, 2 x CH*H*), 0.97 (3H, d, J = 6.1 Hz, CH_3); **^{13}C NMR** ($CDCl_3$, 75 MHz) δ: 137.2, 137.0, 126.8, 126.8, 126.0, 123.3, 121.8, 121.3, 121.3, 121.2, 121.0, 120.8, 118.7, 118.6, 111.3, 111.1, 45.8, 40.2, 36.5, 35.7, 28.7, 23.2, 23.1; **MS (ESI)** m/z 329 $[M+H]^+$.

3,3′-(Propane-2,2-diyl)bis(1*H*-Indole) (25)[13]

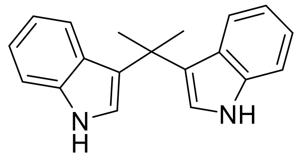

25

Reaction time: **3 h**; Yield: **28%**; White solid; m.p.: 165-167 °C; Eluent: Petroleum ether / Ethyl acetate 7:1; NMR data in accordance with reported literature.[13]

^{1}H NMR ($CDCl_3$, 300 MHz) δ: 7.91 (2H, br s, 2 x NH), 7.42 (2H, d, J = 8.0 Hz, ArH), 7.32 (2H, d, J = 8.0 Hz, ArH), 7.10–7.05 (4H, m, ArH), 6.88 (2H, t, J = 8.0 Hz, ArH), 1.92 (6H, s, 2 x CH_3); **^{13}C NMR** ($CDCl_3$, 75 MHz) δ: 137.3, 126.5, 125.7, 121.5, 121.4, 120.6, 118.8, 111.1, 35.1, 30.1; **MS (ESI)** m/z 297 $[M+Na]^+$.

3,3´-(Phenylmethylene)bis(1-methyl-1*H*-Indole) (26)[14]

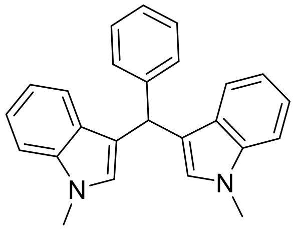

26

Reaction time: **3 h**; Yield: **91%**; Red solid; m.p.: 160-161 °C; Eluent: Petroleum ether / Ethyl acetate 10:1; NMR data in accordance with reported literature.[14]

^{1}H NMR ($CDCl_3$, 500 MHz) *δ;* 7.38 (2H, d, *J* = 8.0 Hz, ArH), 7.34 (2H, d, *J* = 8.0 Hz, ArH), 7.31 – 7.24 (5H, m, ArH), 7.24 – 7.16 (2H, m, ArH), 6.99 (2H, t, *J* = 6.0 Hz, ArH), 6.53 (2H, s, ArH), 5.88 (1H, s, CH), 3.68 (6H, s, 2 x CH_3); **^{13}C NMR** ($CDCl_3$, 125 MHz) *δ*: 144.6, 137.6, 128.8, 128.4, 128.3, 127.6, 126.1, 121.6, 120.2, 118.8, 118.4, 109.2, 40.2, 32.8; **MS (ESI)** m/z 351 $[M+H]^+$.

3,3´-(Phenylmethylene)bis(1-benzyl-1*H*-Indole) (27)[16]

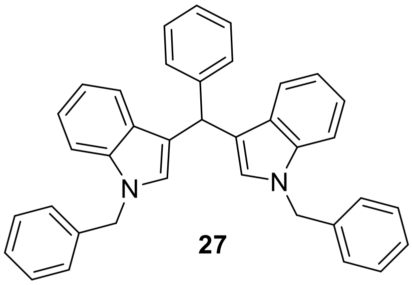

27

Reaction time: **3 h**; Yield: **78%**; White solid; m.p.: 133-135 °C; Eluent: Petroleum ether / Ethyl acetate 10:1; NMR data in accordance with reported literature.[16]

^{1}H NMR ($CDCl_3$, 500 MHz) *δ*: δ 7.45 (2H, d, *J* = 7.9 Hz, ArH), 7.41 (2H, d, *J* = 7.9 Hz, ArH) 7.34 – 7.22 (11H, m, ArH), 7.15 (2H, t, *J* = 7.4 Hz, ArH), 7.06 (4H, d, *J* = 6.0 Hz, ArH), 7.01 (2H, t, *J* = 7.4 Hz, ArH), 6.69 (2H, s, ArH), 5.97 (1H, s, CH), 5.24 (4H, s, 2 x CH_2); **^{13}C NMR** ($CDCl_3$, 75 MHz) *δ*: 144.2, 138.0, 137.2, 128.9, 128.8, 128.8, 128.4, 128.0, 127.9, 127.5, 126.6, 121.8, 120.3, 119.1, 118.9, 109.8, 50.1, 40.4; **MS (ESI)** m/z 503 $[M+H]^+$.

3,3′-(Phenylmethylene)bis(1-(prop-2-yn-1-yl)-1*H*-Indole) (28)[17]

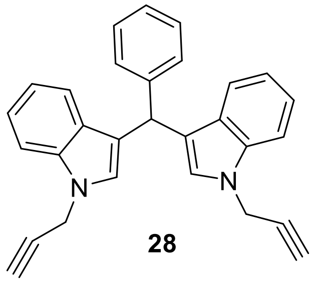

28

Reaction time: **3 h**; Yield: **87%**; Colourless solid; m.p.: 149-151 °C; Eluent: Petroleum ether / Ethyl acetate 10:1; NMR data in accordance with reported literature.[17]

^{1}H NMR ($CDCl_3$, 300 MHz) *δ*: 7.43 – 7.35 (6H, m, ArH), 7.31 – 7.21 (5H, m, ArH), 7.03 (2H, t, *J* = 7.6 Hz, ArH), 6.66 (2H, s, ArH), 5.88 (1H, s, CH), 4.76 (4H, d, *J* = 2.5 Hz, CH_2), 2.33 (2H, t, *J* = 2.5 Hz, 2 x CH); **^{13}C NMR** ($CDCl_3$, 75 MHz) *δ*: 144.0, 136.7, 128.8, 128.4, 128.1, 127.0, 126.3, 122.0, 120.4, 119.5, 119.2, 109.5, 78.2, 73.3, 40.3, 35.9; **MS (ESI)** m/z 399 $[M+H]^+$.

3,3′-(Phenylmethylene)bis(2-methyl-1*H*-Indole) (29)[18]

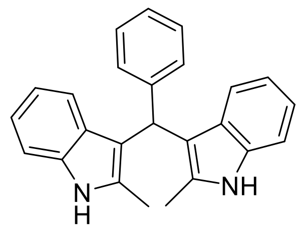

29

Reaction time: **3 h**; Yield: **67%**; Red solid; m.p.: 230-231 °C; Eluent: Petroleum ether / Ethyl acetate 5:1; NMR data in accordance with reported literature.[18]

^{1}H NMR ($CDCl_3$, 300 MHz) *δ*: ^{1}H NMR (300 MHz, $CDCl_3$) δ 7.73 (2H, br s, 2 x NH), 7.26 – 7.20 (7H, m, ArH), 7.06 – 6.96 (4H, m, ArH), 6.85 (2H, t, *J* = 7.5 Hz, ArH), 6.01 (1H, s, CH) 2.06 (6H, s, 2 x CH_3); **^{13}C NMR** ($CDCl_3$, 75 MHz) *δ*: 143.9, 135.2, 131.9, 129.2, 129.1, 128.2, 126.1, 120.7, 119.5, 119.2, 113.5, 110.1, 39.4, 12.6; **MS (ESI)** m/z 351 $[M+H]^+$.

3,3′-(Phenylmethylene)bis(5-methoxy-1*H*-Indole) (30)[12]

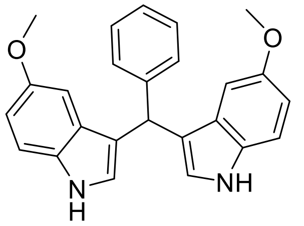

30

Reaction time: **3 h**; Yield: **83%**; Orange oil; Eluent: Petroleum ether / Ethyl acetate 10:1; NMR data in accordance with reported literature.[12]

^{1}H NMR ($CDCl_3$, 300 MHz) *δ:* 7.85 (2H, br s, 2 x NH), 7.39 – 7.32 (2H, m, ArH), 7.31–7.22 (6H, m, ArH), 6.87–6.82 (3H, m, ArH), 6.68 (2H, s, ArH), 5.79 (1H, s, CH), 3.71 (6H, s, 2 x OCH_3); **^{13}C NMR** ($CDCl_3$, 75 MHz) *δ*: 153.7, 143.9, 131.9, 128.7, 128.2, 127.5, 126.1, 124.4, 119.3, 111.9, 111.7, 102.0, 55.9, 40.3; **MS (ESI)** m/z 383 $[M+H]^+$.

3,3′-(Phenylmethylene)bis(5-bromo-1*H*-Indole) (31)[7]

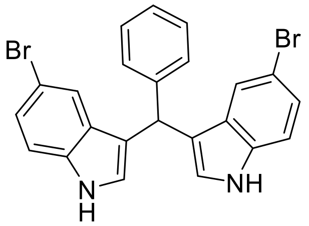

31

Reaction time: **3 h**; Yield: **82%**; Red solid; m.p.: 240-242 °C; Eluent: Petroleum ether / Ethyl acetate 10:1; NMR data in accordance with reported literature.[7]

^{1}H NMR ($CDCl_3$, 300 MHz) *δ*: 7.95 (2H, br s, 2 x NH), 7.47 (2H, s, ArH), 7.30 – 7.29 (4H, m, ArH), 7.25 – 7.21 (5H, m, ArH), 6.64 (2H, s, ArH), 5.75 (1H, s, CH); **^{13}C NMR** ($CDCl_3$, 75 MHz) *δ*: 143.2, 135.5, 128.8, 128.7, 128.6, 126.7, 125.1, 124.9, 122.4, 119.2, 112.8, 112.7, 40.0; **MS (ESI)** m/z 478 $[M+H]^+$.

Mechanistic Investigations with NMR

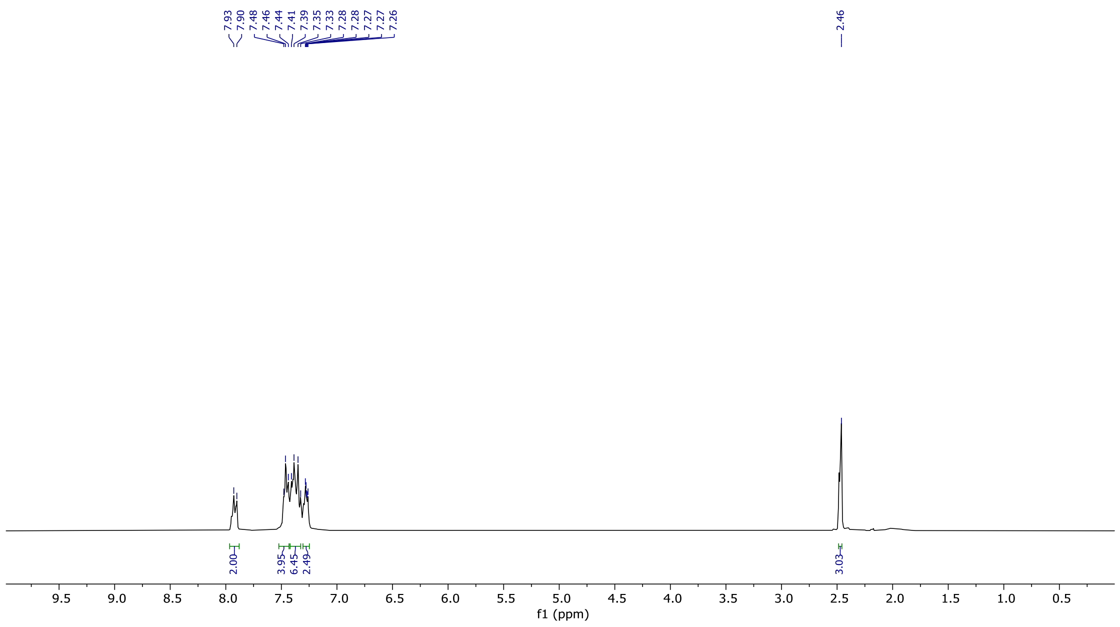

^{1}H NMR (300 MHz, $CDCl_3$) of diphenylmethanone *O*-tosyl oxime (**3**) (10 mg, 0.028 mmol) before irradiation.

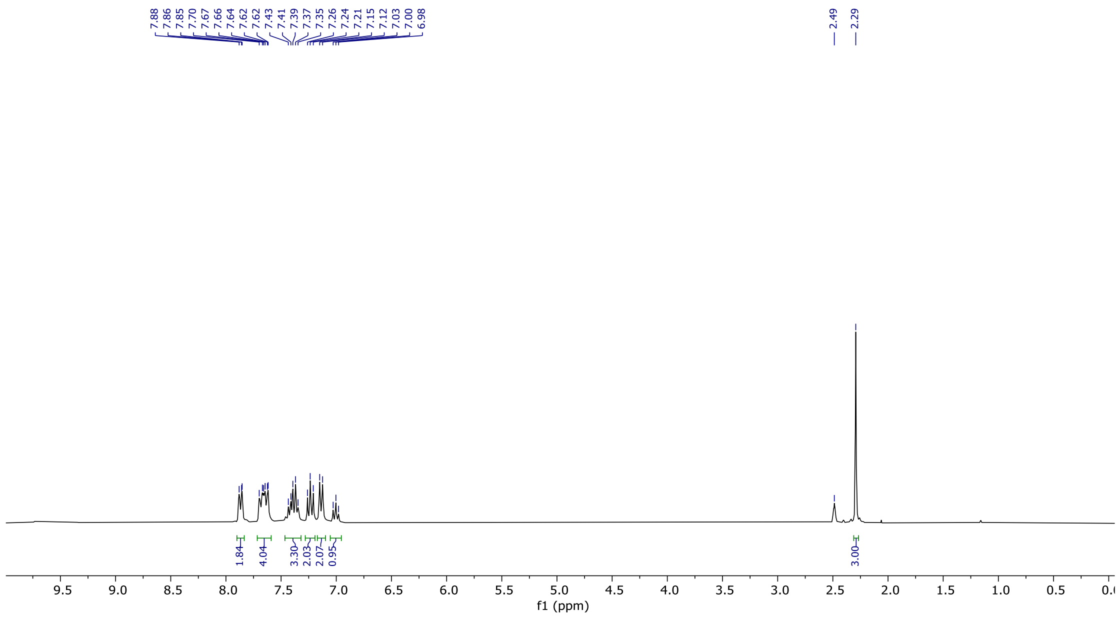

^{1}H NMR (300 MHz, $CDCl_3$ plus few drops of DMSO-d6 due to poor solubility of the generated p-TSA in $CDCl_3$) of diphenylmethanone *O*-tosyl oxime (**3**) (10 mg, 0.028 mmol) after irradiation at 390 nm for 1 h.

Mechanistic Investigation with UV-Vis

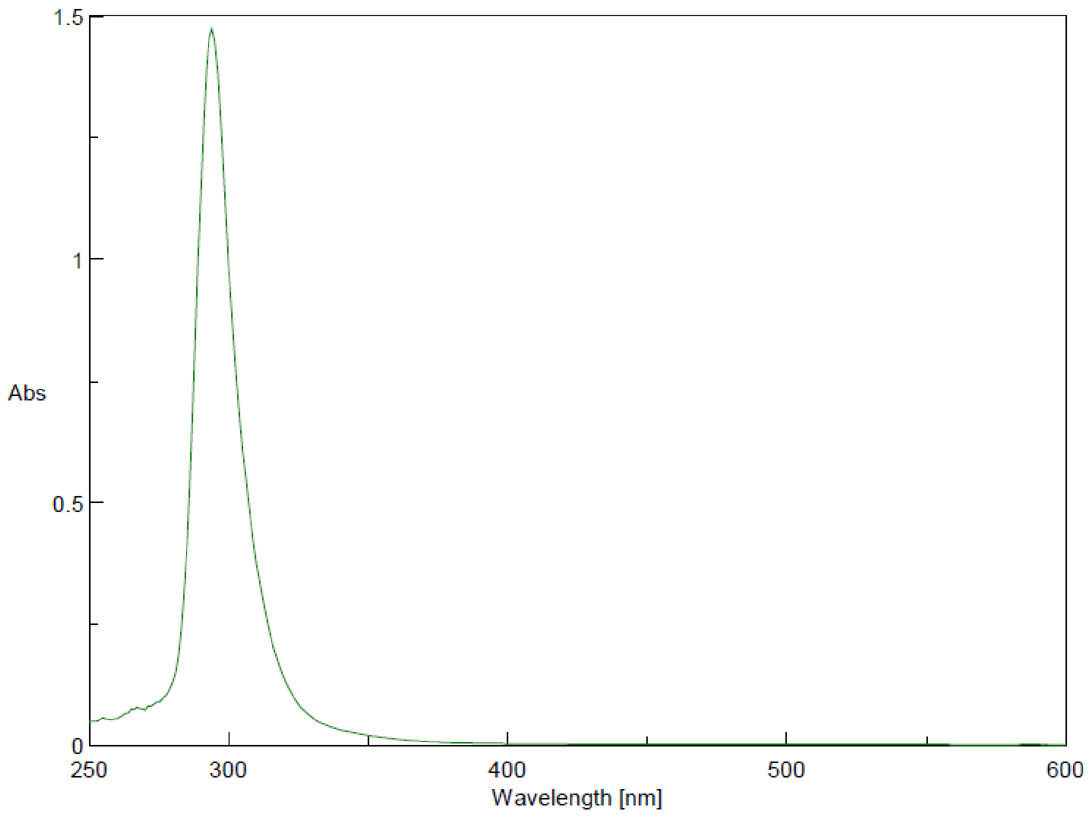

UV-Vis absorbance spectrum of diphenylmethanone *O*-tosyl oxime (**3**) (10^{-3} M) in MeCN.

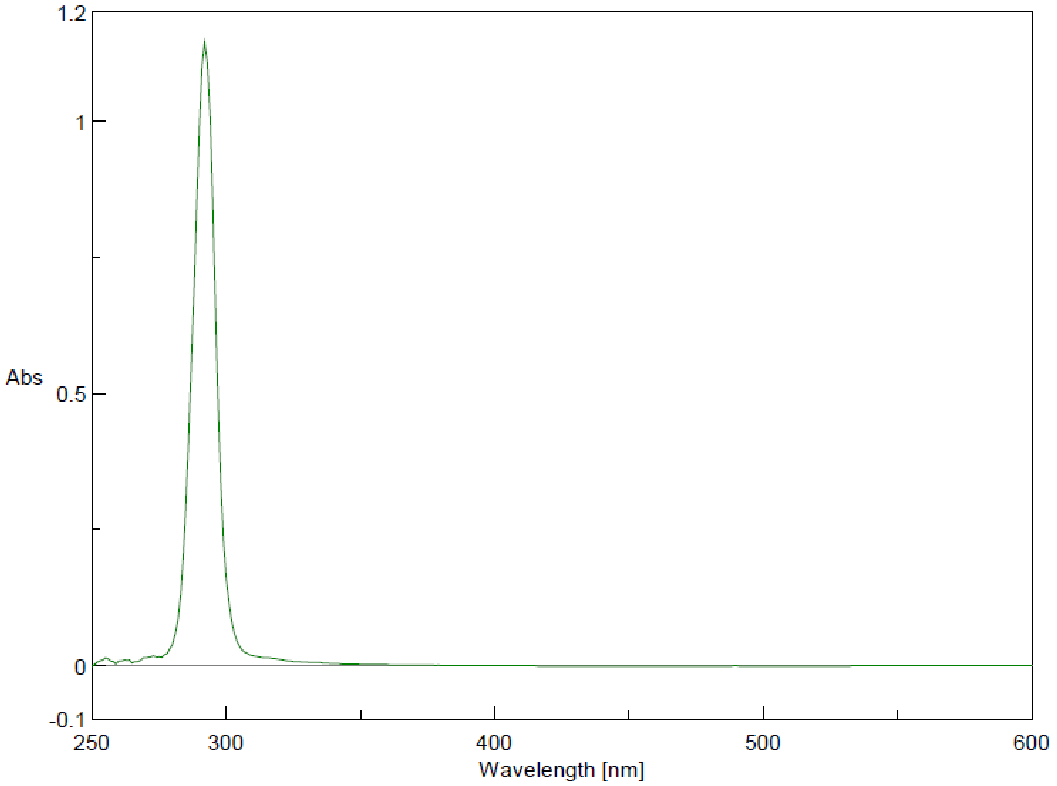

UV-Vis absorbance spectrum of indole (**2a**) (10^{-3} M) in MeCN.

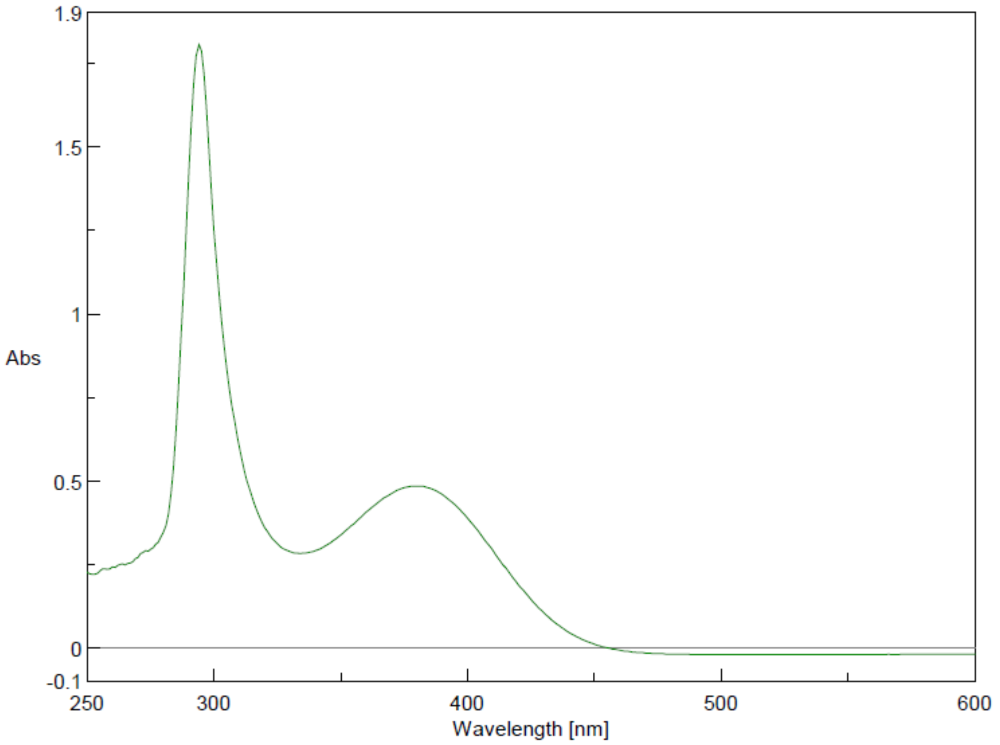

UV-Vis absorbance spectrum of the mixture diphenylmethanone *O*-tosyl oxime (**3**) (10^{-3} M) and indole (**2a**) (10^{-3} M) in MeCN.

Mechanistic Investigation with DI-HRMS

Instrumentation

High Resolution Mass Spectra were recorded with a Q-TOF (Time of Flight Mass Spectrometer) Bruker Maxis Impact with electrospray ionization (ESI) source. N_2 was used as collision gas and positive ionization mode was used for all MS experiments. The data acquisition was carried out with Data Analysis from Bruker Daltonics (version 4.1). Acetonitrile LC-MS gradient was obtained from Carlo Erba Reagents (Chaussée du Vexin, France). Source conditions: End plate offset 500V, Capillary 4500V, Nebulizer 0.4 bar, dry gas 4.0 L/min, dry temperature 180 ºC and Quadrupole conditions: Ion energy 5 eV, Collision energy 10 eV, Transfer time 143 μs, Collision ion RF 3500 vpp, Pre pulse storage 1μs.

HRMS studies were performed with an ESI source under positive-ionization mode. The annotation of the intermediates was based on the exact mass high accuracy (mass error lower than 5 ppm).

Products of the reaction between aldehyde (1a) with indole (2a)

The mechanism of the reaction between aldehyde (**1a**) with indole (**2a**) was studied by HRMS. Benzaldehyde (**1a**) (21 mg, 0.20 mmol, 1.00 equiv.), indole (**2a**) (51 mg, 0.44 mmol, 2.20 equiv.) and diphenylmethanone *O*-tosyl oxime (**3**) (2 mg, 2.5 mol%) were diluted in EtOAc (0.5 mL) and TEMPO (31 mg, 0.20 mmol, 1.00 equiv.) was added. Then, the test tube was closed with a septum and sealed with parafilm, and the reaction mixture was degassed under an argon atmosphere. Then, the test tube was left stirring under Kessil lamp irradiation (390 nm) for 3 h. In the reaction mixture, peaks that correspond to intermediates **I** and **II** were identified.

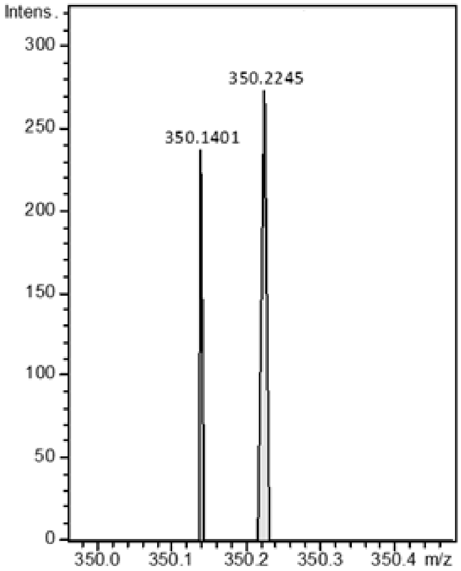

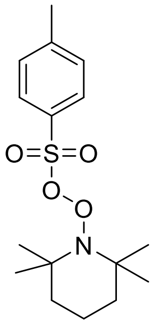

I

Exact Mass $[M+Na]^+$	350.1397
Found $[M+Na]^+$	350.1401

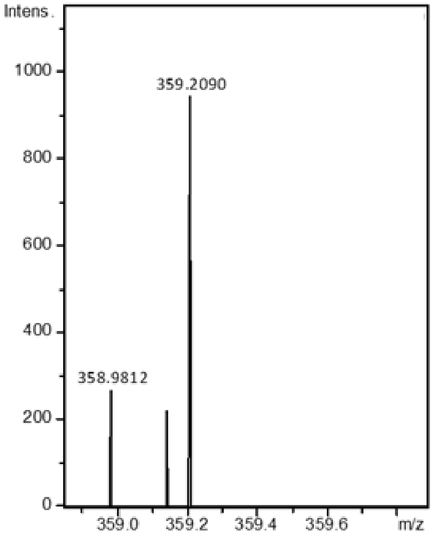

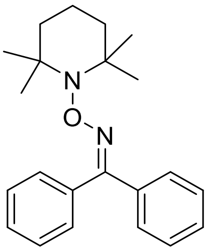

II

Exact Mass $[M+Na]^+$	359.2094
Found $[M+Na]^+$	359.2090

References

1. C. Ramalingan, Y. T. Park, *J. Org. Chem.* **2007**, *72*, 4536–4538.

2. G. V. Shustov, N. B. Tavakalyan, A. P. Pleshkova, R. G. Kostyanovski, *Chemistry of Heterocyclic Compounds* **1981**, *17*, 600-605.

3. E. M. Galathri, T. J. Kuczmera, B. J. Nachtsheim, C. G. Kokotos, *Green Chem.* **2024**, *26*, 825–831.

4. J. Li, X. Sun, E. Dmitrieva, N. Israel, F. Wu, L. Yang, R. Liu, X. Feng, B. Plietker *Org. Lett.* **2024**, *26*, 1046–1050

5. T. M. Masson, S. D. A. Zondag, J. H. A. Schuurmans, T. Noel, *React. Chem. Eng.* **2024**, *9*, 2218-2225.

6. A. Mondal, R. Sharma, B. Dutta, D. Pal, D. Srimani, *J. Org. Chem.* **2022**, *87*, 3989–4000.

7. S. Zhong, X. Fang, Y. Wang, G. Zhang, Y. Li, Y. Li, *Org. Lett.* **2022**, *24*, 1022–1026.

8. P. R. Simha, M. S. Mangali, D. Kuppireddy Gari, P. Venkatapuram, P. Adivireddy, *J. Heterocycl. Chem.* **2017**, *54*, 2717-2724.

9. R. Athavale, S. Gardi, F. Choudhary, D. Patil, N. Chandan, P. *More, Appl. Catal. A, Gen.* **2024**, *669*, 119505-119519.

10. V. D. Kadu, D. N. Nadimetla, M. G. Hublikar, D. G. Raut, R. B. Bhosale, *Lett. Org. Chem*. **2020**, *17*, 61–67.

11. Y. Zhang, X. Chen, J. Liang, Z. C. Shang, *Synth. Commun*. **2011**, *41*, 2446–2454.

12. A. K. Guin, S. Pal, S. Chakraborty, S. Chakraborty, N. D. Paul, *J. Org. Chem.* **2023**, *88*, 16755–16772.

13. J. Yang, J. Zhang, T. Li, Y. Liu, H. Jin, D. H. Ryu, L. Zhang, *Adv. Synth. Catal.*

2025, *367*, e202401542.

14. S. Badigenchala, D. Ganapathy, A. Das, R. Singh, G. Sekar, *Synthesis* **2014**, *46*, 101-109.

15. X. Liu, S. Ma, P. H. Toy, *Org. Lett.* **2019**, *21*, 9212–9216.

16. L. Zheng, F. Gao, C. Yang, G. L. Gao, Y. Zhao, Y. Gao, W. Xia, *Org. Lett.* **2017**, *19*, 5086–5089.

17. M. Damodiran, D. Muralidharan, P. T. Perumal, *Bioorg. Med. Chem. Lett.* **2009**, *19*, 3611–3614.

18. H. Meng, J. Feng, M. Yu, Z. F. Xu, C. Y. Li, *Eur. J. Org. Chem.* **2025**, *28*, e202500515.

NMR Spectra

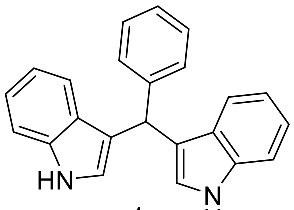

4

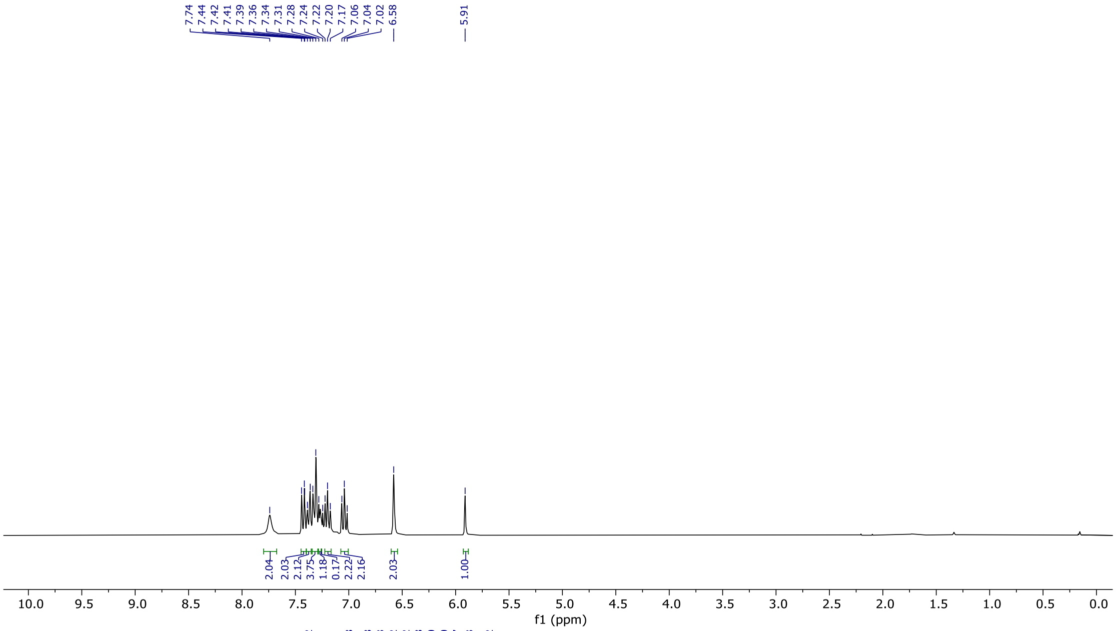

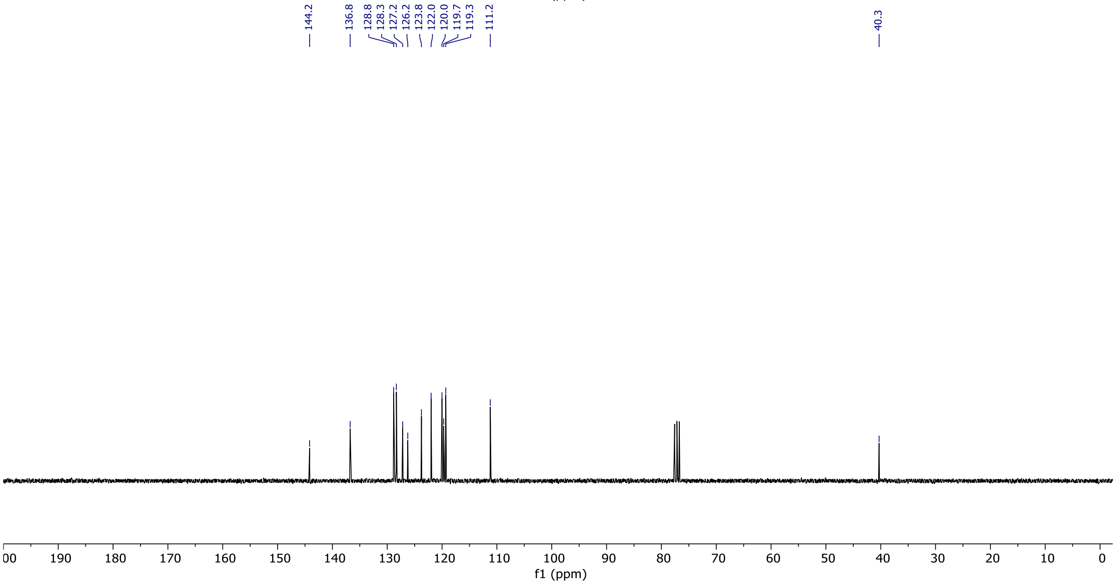

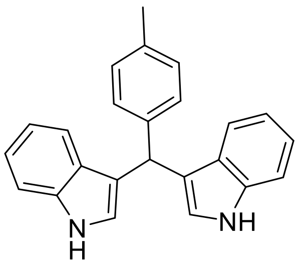
N
H
NH

5

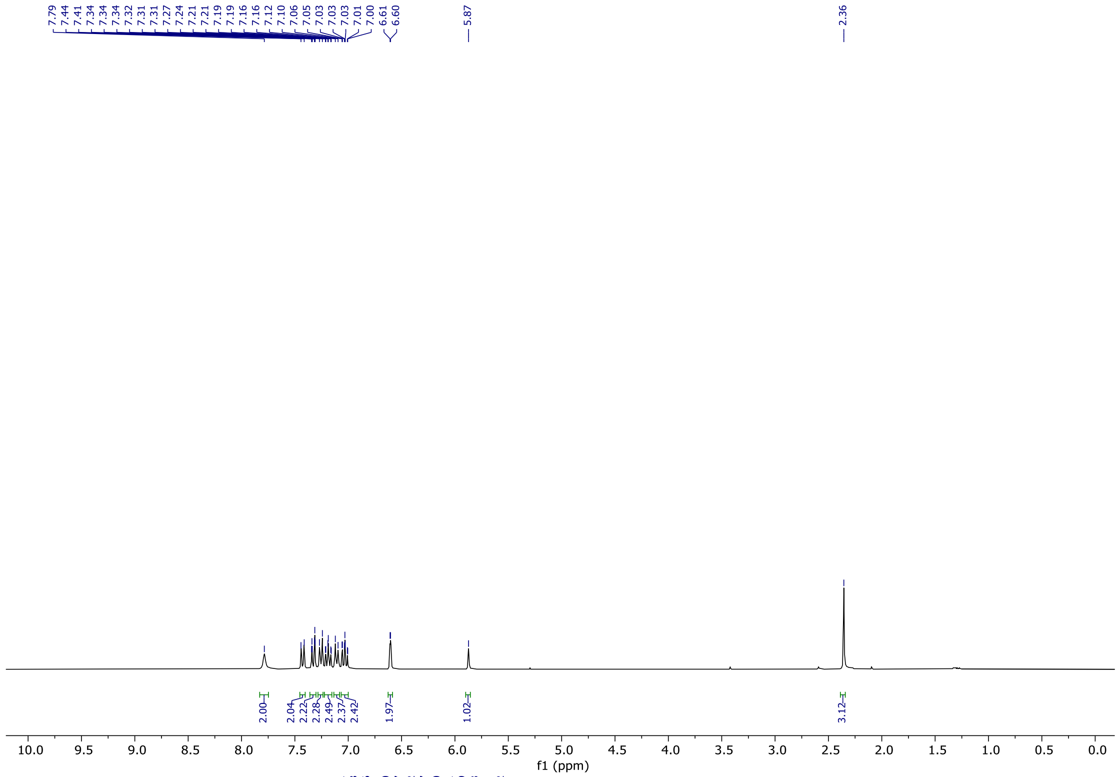
7.79
7.44
7.41
7.34
7.34
7.34
7.32
7.31
7.31
7.27
7.24
7.21
7.21
7.19
7.19
7.16
7.16
7.12
7.10
7.06
7.05
7.03
7.03
7.03
7.01
7.00
6.61
6.60
5.87
2.36
2.00
2.04
2.22
2.28
2.49
2.37
2.42
1.97
1.02
3.12
f1 (ppm)

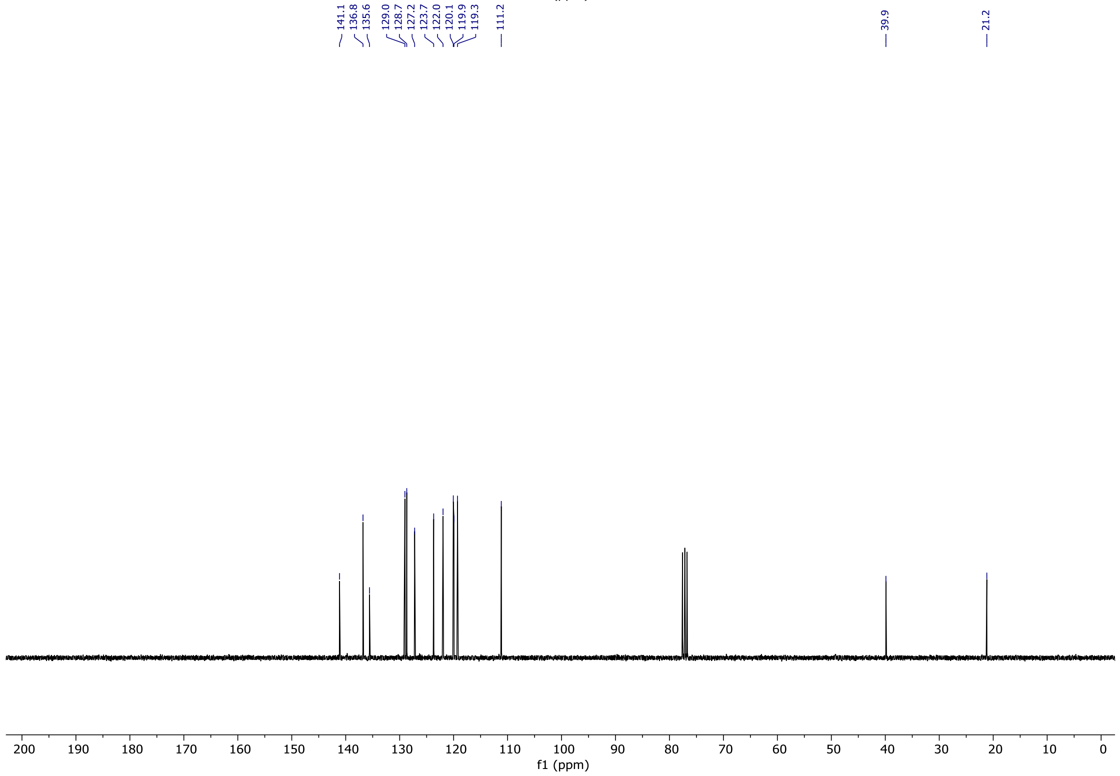
141.1
136.8
135.6
129.0
128.7
127.2
123.7
122.0
120.1
119.9
119.3
111.2
39.9
21.2
f1 (ppm)

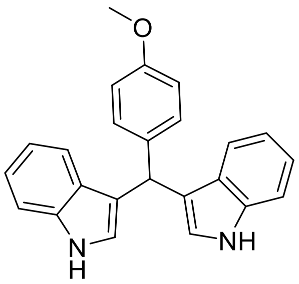
O
N
H
NH

6

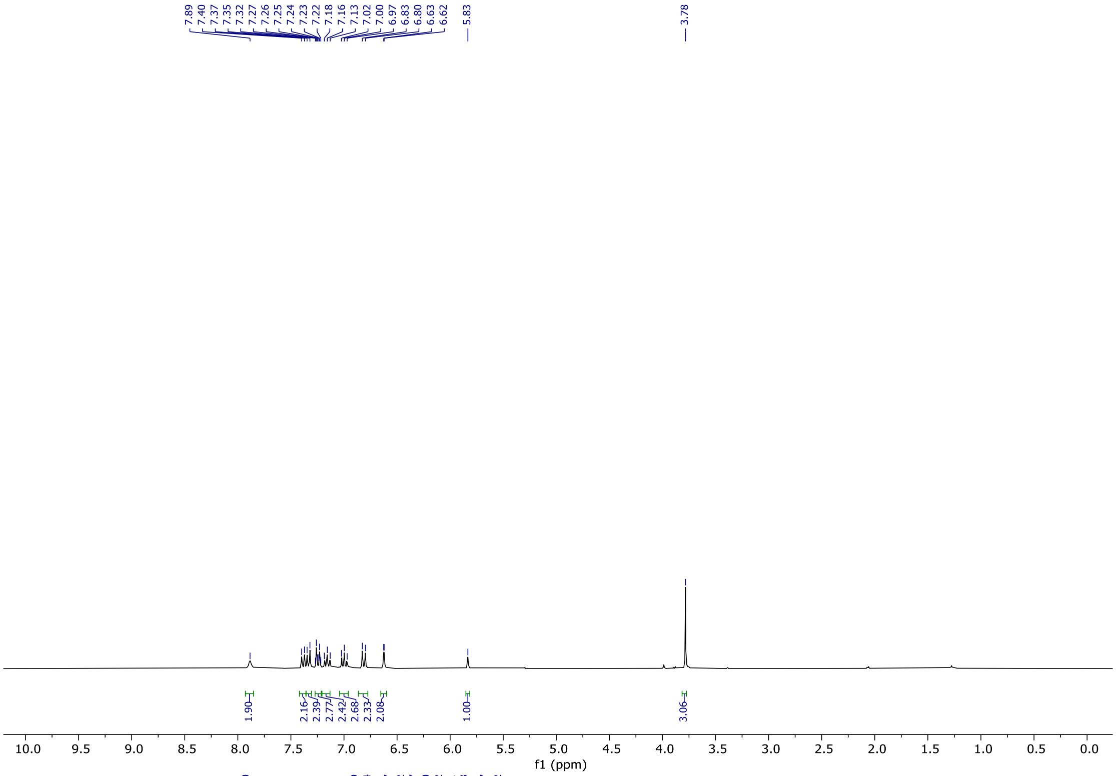
7.89
7.40
7.37
7.35
7.32
7.27
7.26
7.25
7.24
7.23
7.22
7.18
7.16
7.13
7.02
7.00
6.97
6.83
6.80
6.63
6.62
5.83
3.78
1.90
2.16
2.39
2.77
2.42
2.68
2.33
2.08
1.00
3.06
f1 (ppm)

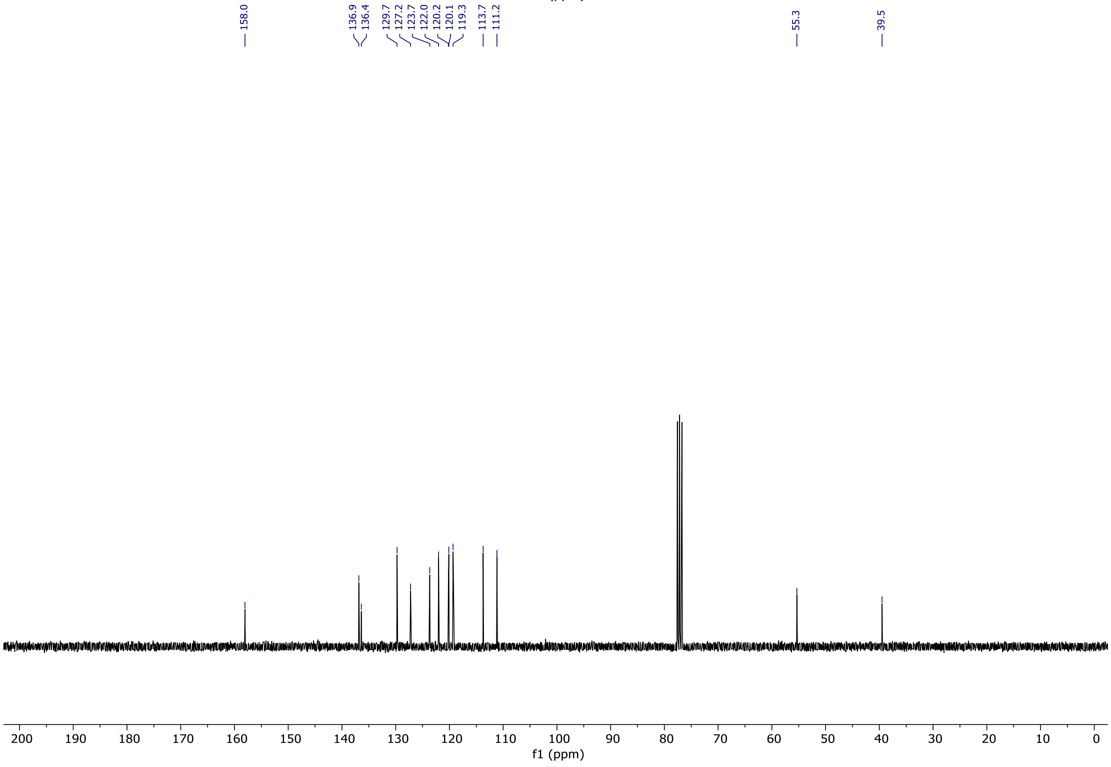
158.0
136.9
136.4
129.7
127.2
123.7
122.0
120.2
120.1
119.3
113.7
111.2
55.3
39.5
f1 (ppm)

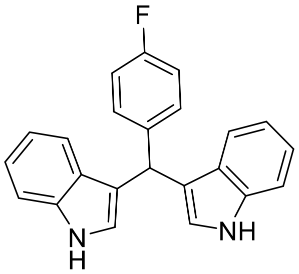
F
N
H
NH

7

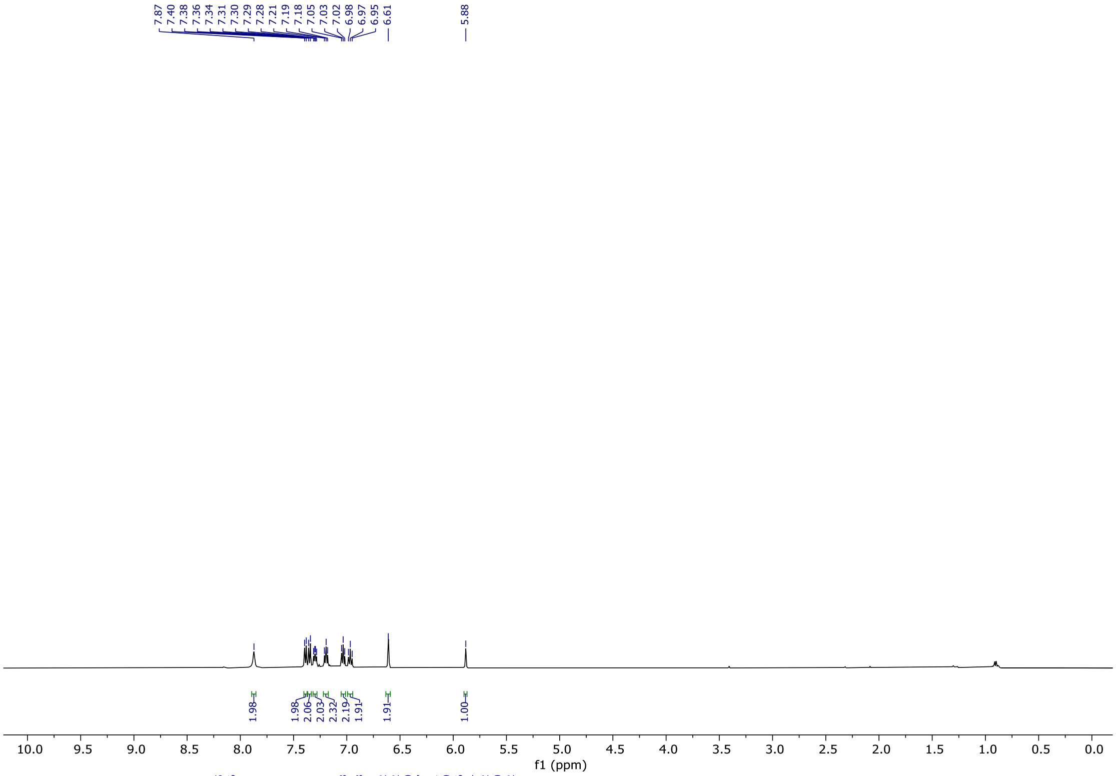
7.87
7.40
7.38
7.36
7.34
7.31
7.30
7.29
7.28
7.21
7.19
7.18
7.05
7.03
7.02
6.98
6.97
6.95
6.61
5.88
1.98
1.98
2.06
2.03
2.32
2.19
1.91
1.91
1.00
10.0 9.5 9.0 8.5 8.0 7.5 7.0 6.5 6.0 5.5 5.0 4.5 4.0 3.5 3.0 2.5 2.0 1.5 1.0 0.5 0.0
f1 (ppm)

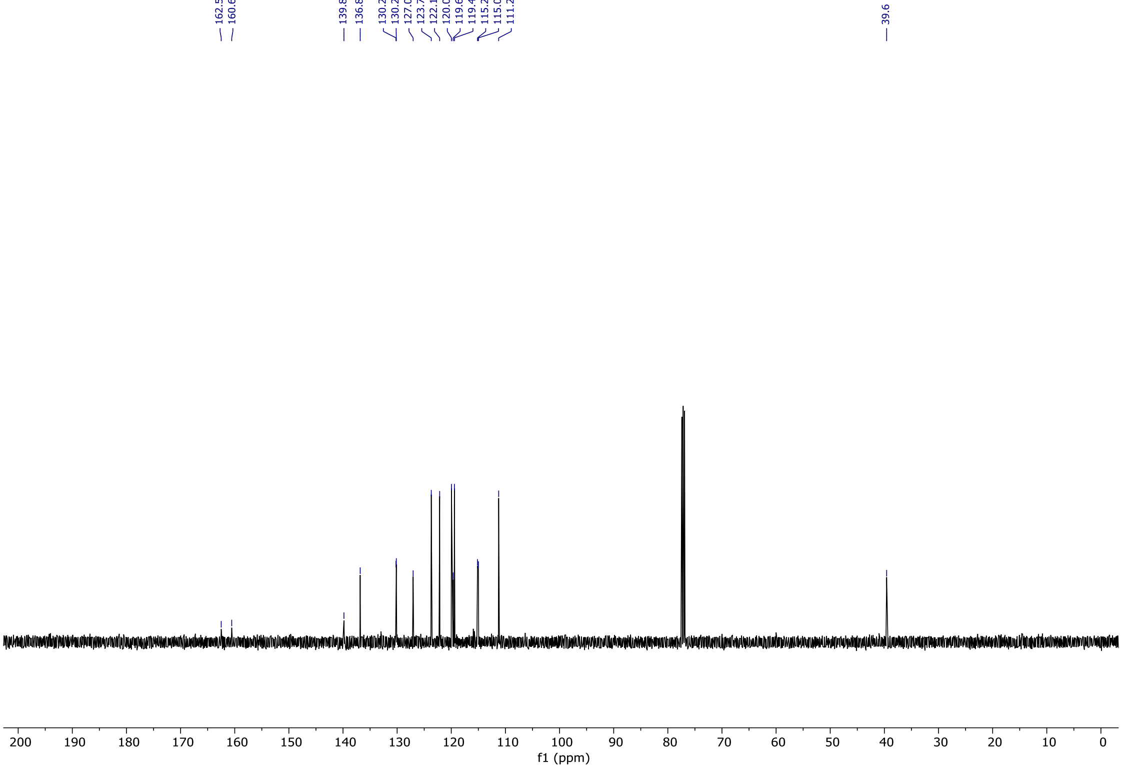
162.5
160.6
139.8
136.8
130.2
130.2
127.0
123.7
122.1
120.0
119.6
119.4
115.2
115.0
111.2
39.6
200 190 180 170 160 150 140 130 120 110 100 90 80 70 60 50 40 30 20 10 0
f1 (ppm)

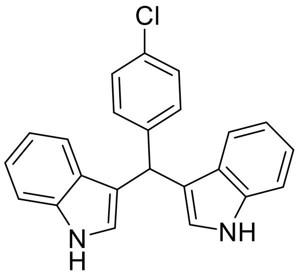
Cl
NH
N
H

8

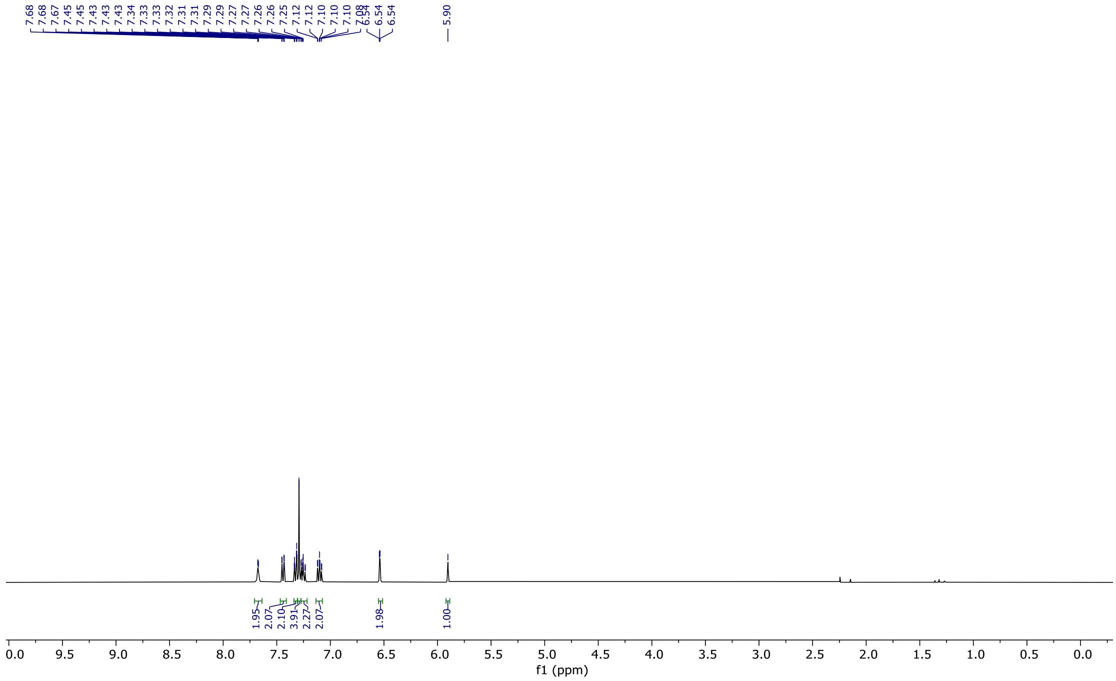
7.68
7.68
7.67
7.45
7.45
7.43
7.43
7.43
7.34
7.33
7.33
7.32
7.31
7.31
7.29
7.29
7.27
7.27
7.26
7.26
7.25
7.12
7.12
7.10
7.10
7.10
7.08
6.54
6.54
6.54
5.90
1.95
2.07
2.10
3.91
2.27
2.07
1.98
1.00
f1 (ppm)

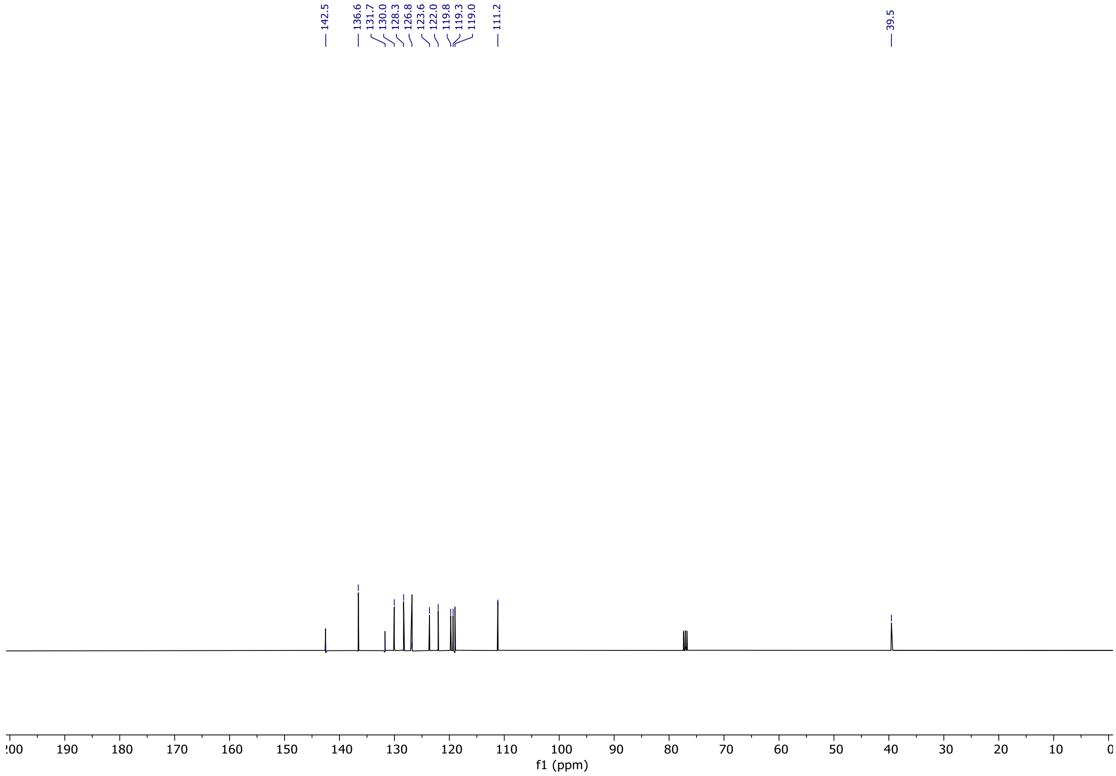
142.5
136.6
131.7
130.0
128.3
126.8
123.6
122.0
119.8
119.3
119.0
111.2
39.5
f1 (ppm)

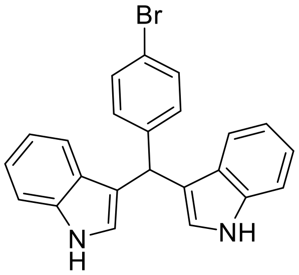
Br
N
H
NH

9

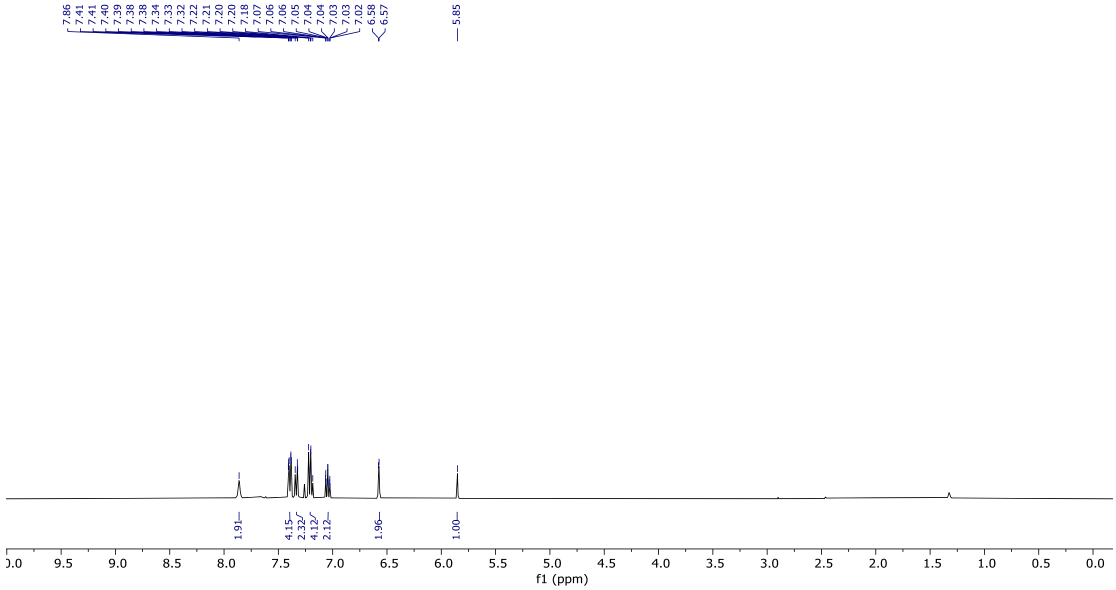
7.86
7.41
7.41
7.40
7.39
7.38
7.38
7.34
7.33
7.32
7.22
7.21
7.20
7.20
7.18
7.07
7.06
7.06
7.05
7.04
7.04
7.03
7.03
7.02
6.58
6.57
5.85
1.91
4.15
2.32
4.12
2.12
1.96
1.00
f1 (ppm)

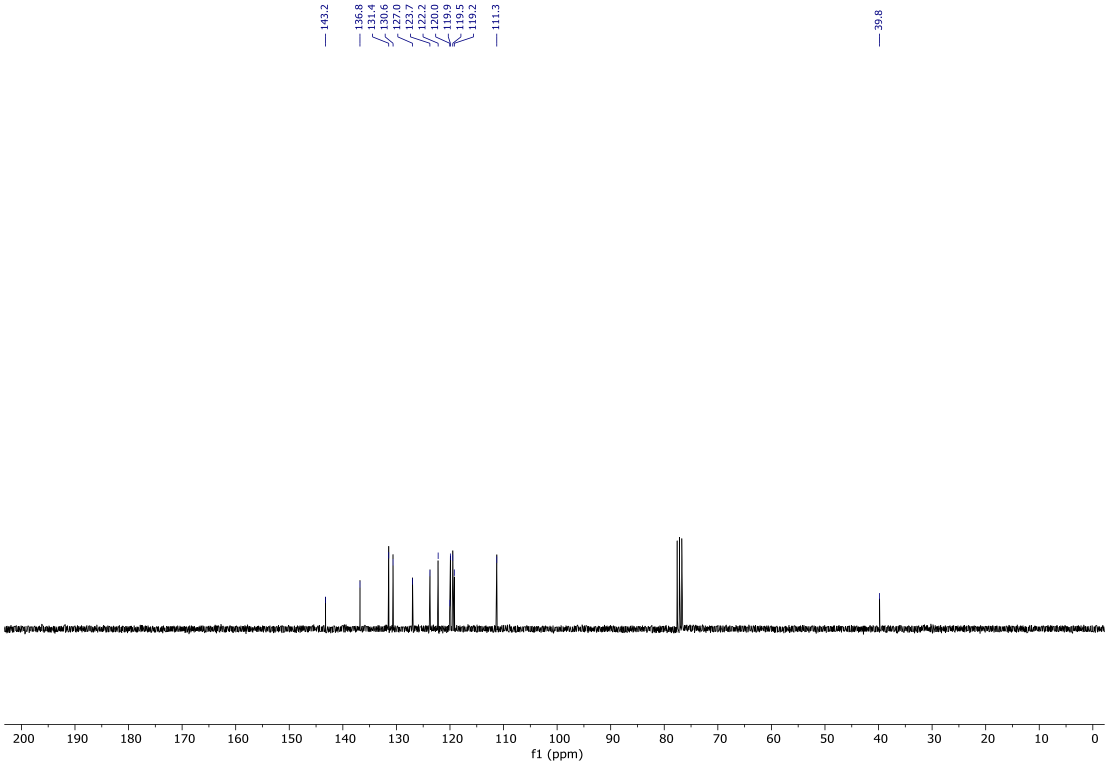
143.2
136.8
131.4
130.6
127.0
123.7
122.2
120.0
119.9
119.5
119.2
111.3
39.8
f1 (ppm)

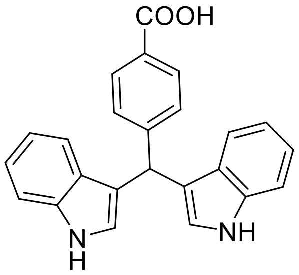
COOH
NH
N
H

10

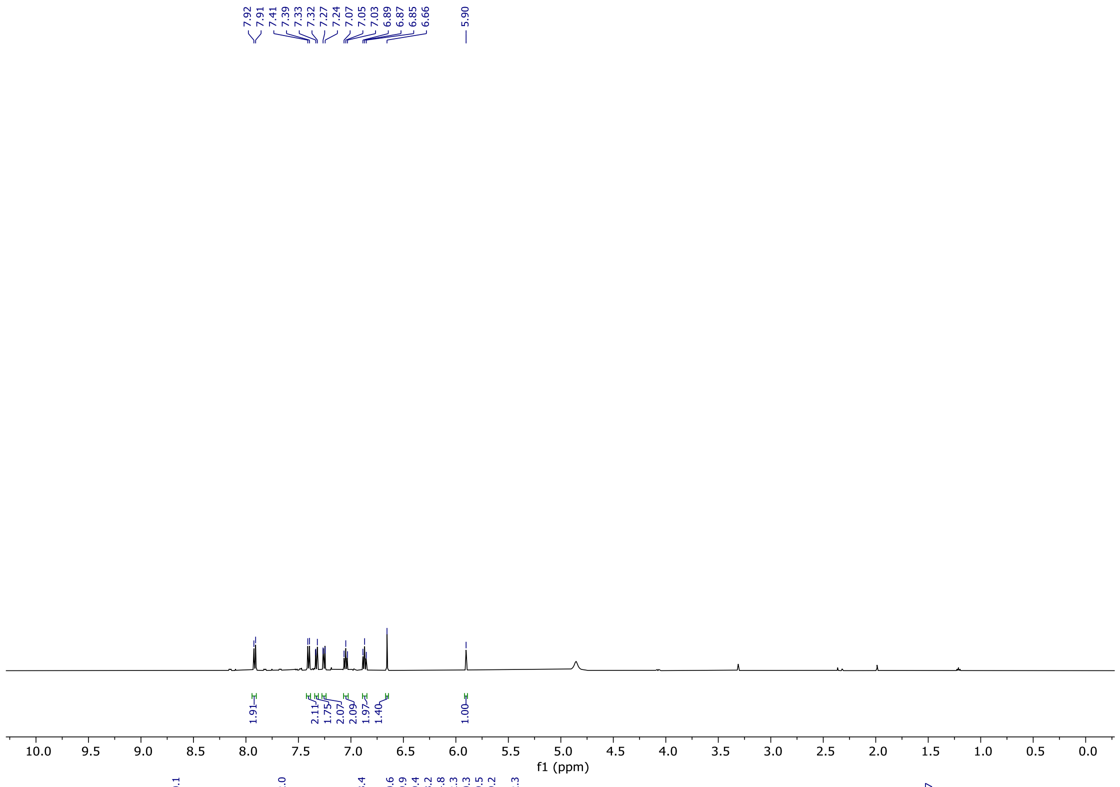
7.92
7.91
7.41
7.39
7.33
7.32
7.27
7.24
7.07
7.05
7.03
6.89
6.87
6.85
6.66
5.90
1.91
2.11
1.75
2.07
2.09
1.97
1.40
1.00
f1 (ppm)

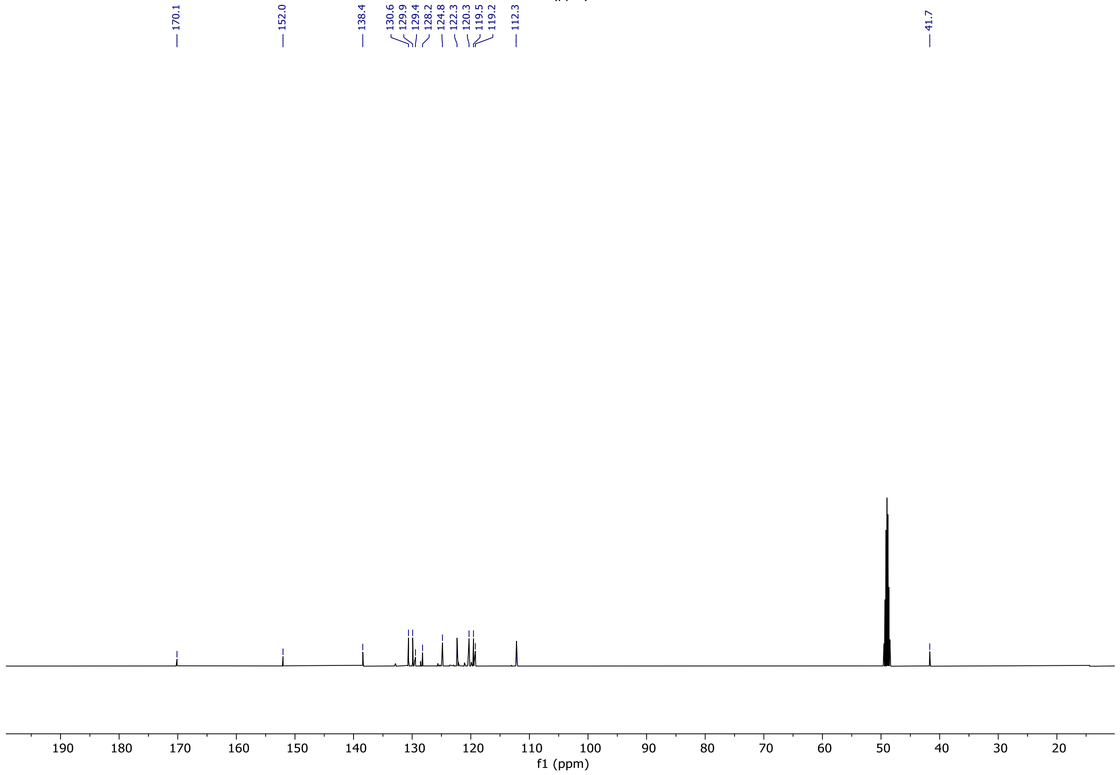
170.1
152.0
138.4
130.6
129.9
129.4
128.2
124.8
122.3
120.3
119.5
119.2
112.3
41.7
f1 (ppm)

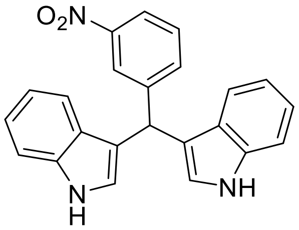
O2N
N
H
NH

11

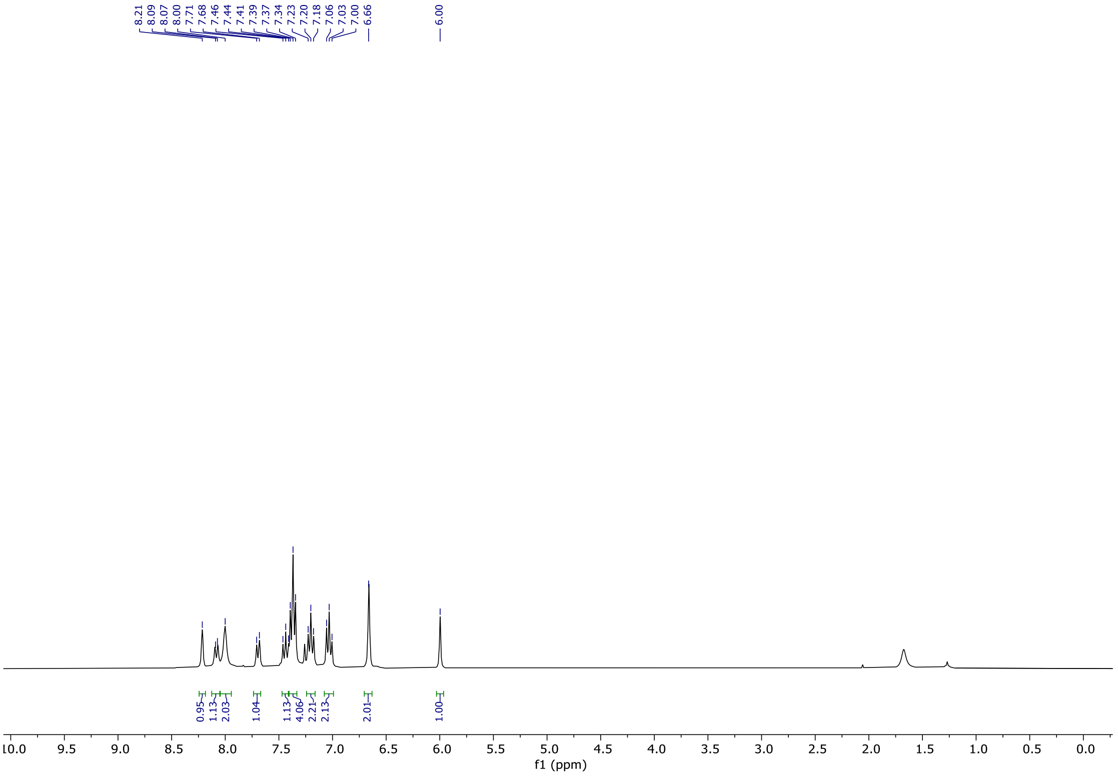
8.21
8.09
8.07
8.00
7.71
7.68
7.46
7.44
7.41
7.39
7.37
7.34
7.23
7.20
7.18
7.06
7.03
7.00
6.66
6.00
0.95
1.13
2.03
1.04
1.13
4.06
2.21
2.13
2.01
1.00
f1 (ppm)

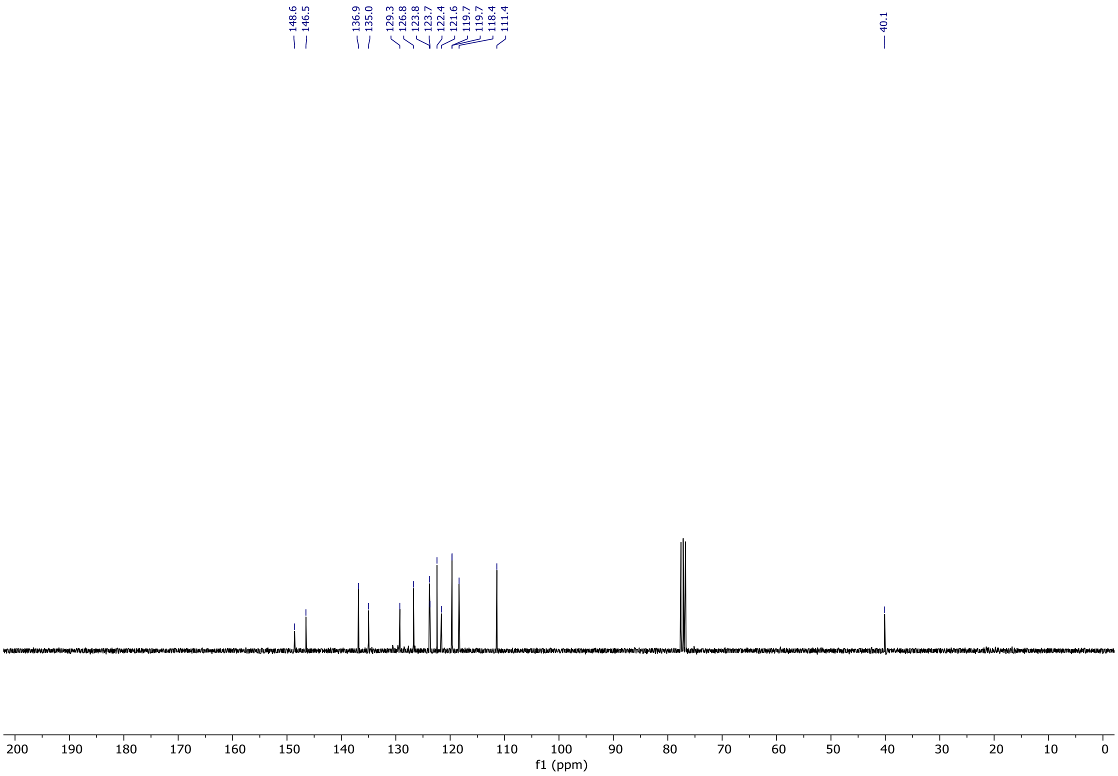
148.6
146.5
136.9
135.0
129.3
126.8
123.8
123.7
122.4
121.6
119.7
119.7
118.4
111.4
40.1
f1 (ppm)

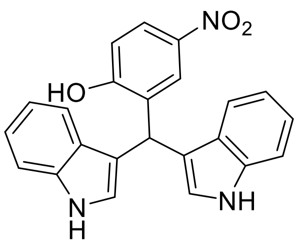

NO2
HO
N
H
NH

12

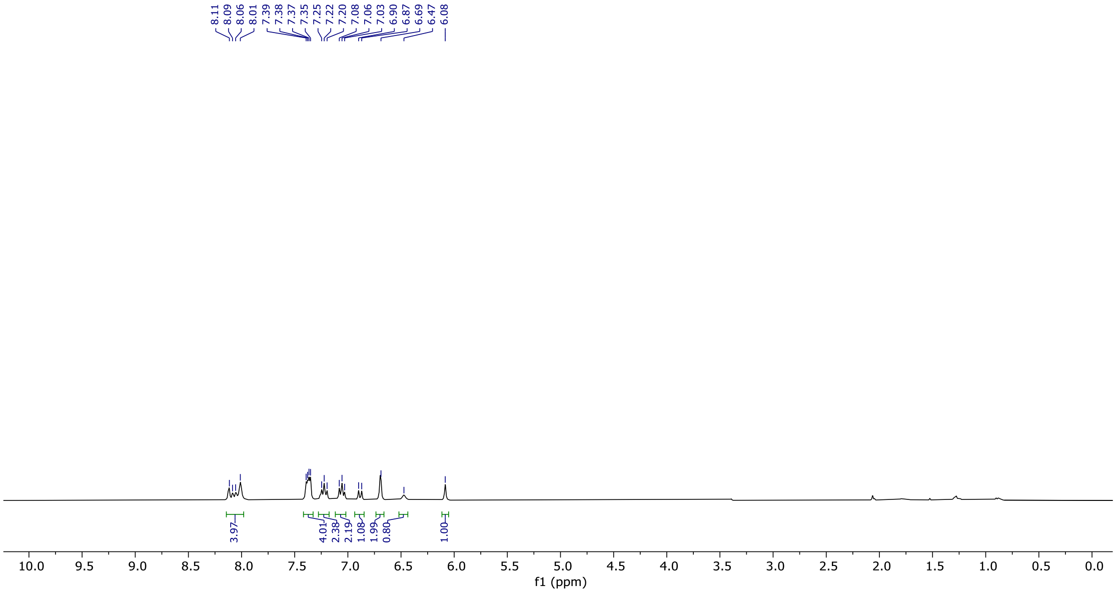

8.11 8.09 8.06 8.01 7.39 7.38 7.37 7.35 7.25 7.22 7.20 7.08 7.06 7.03 6.90 6.87 6.69 6.47 6.08
3.97 4.01 2.38 2.19 1.08 1.99 0.80 1.00
f1 (ppm)

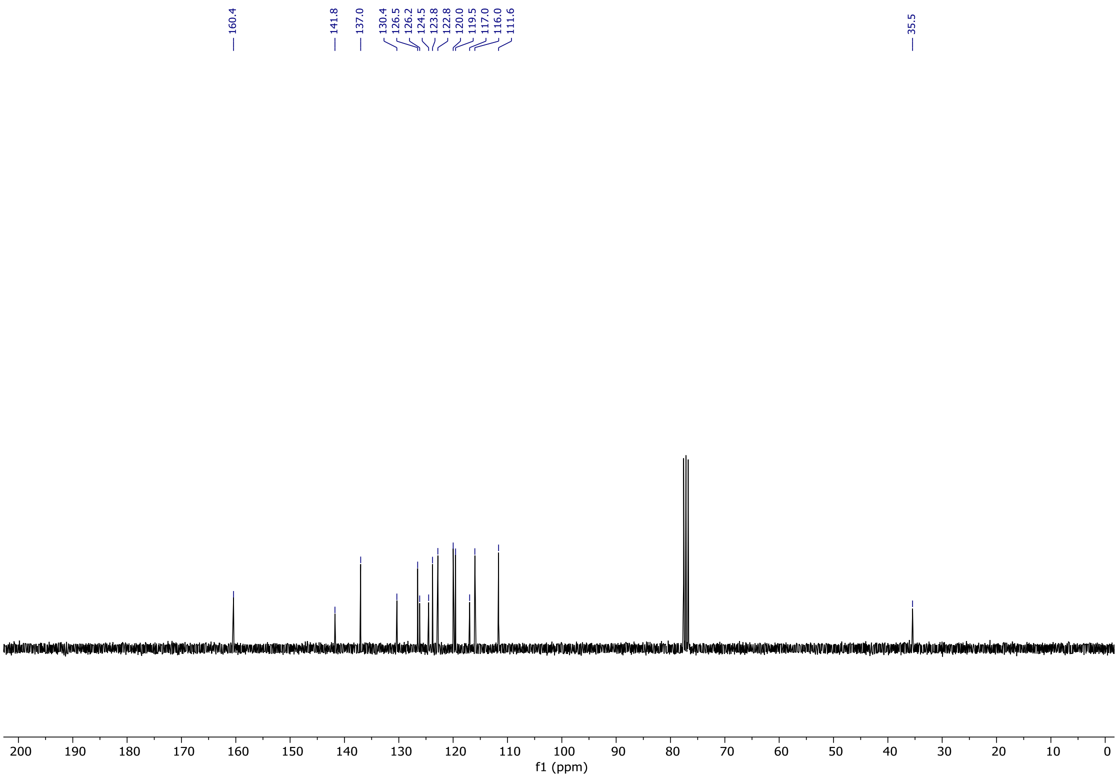

160.4 141.8 137.0 130.4 126.5 126.2 124.5 123.8 122.8 120.0 119.5 117.0 116.0 111.6 35.5
f1 (ppm)

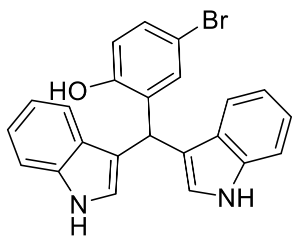
Br
HO
NH
N
H

13

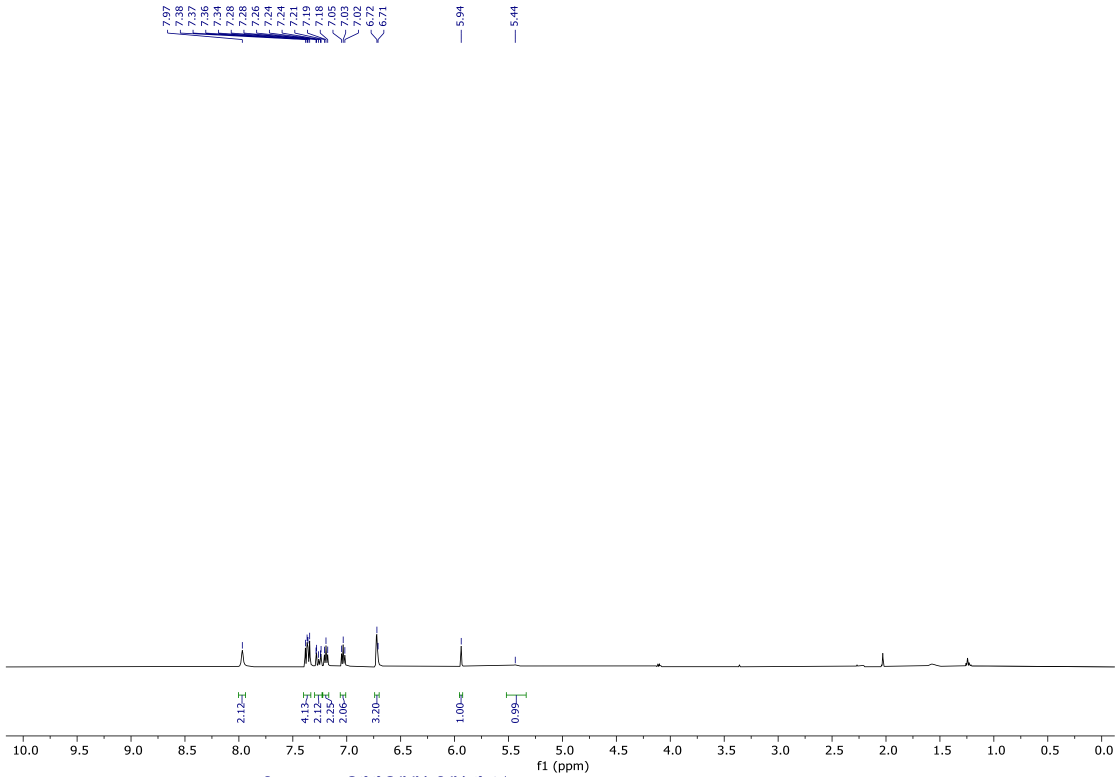
7.97
7.38
7.37
7.36
7.34
7.28
7.28
7.26
7.24
7.24
7.21
7.19
7.18
7.05
7.03
7.02
6.72
6.71
5.94
5.44
2.12
4.13
2.12
2.25
2.06
3.20
1.00
0.99
f1 (ppm)

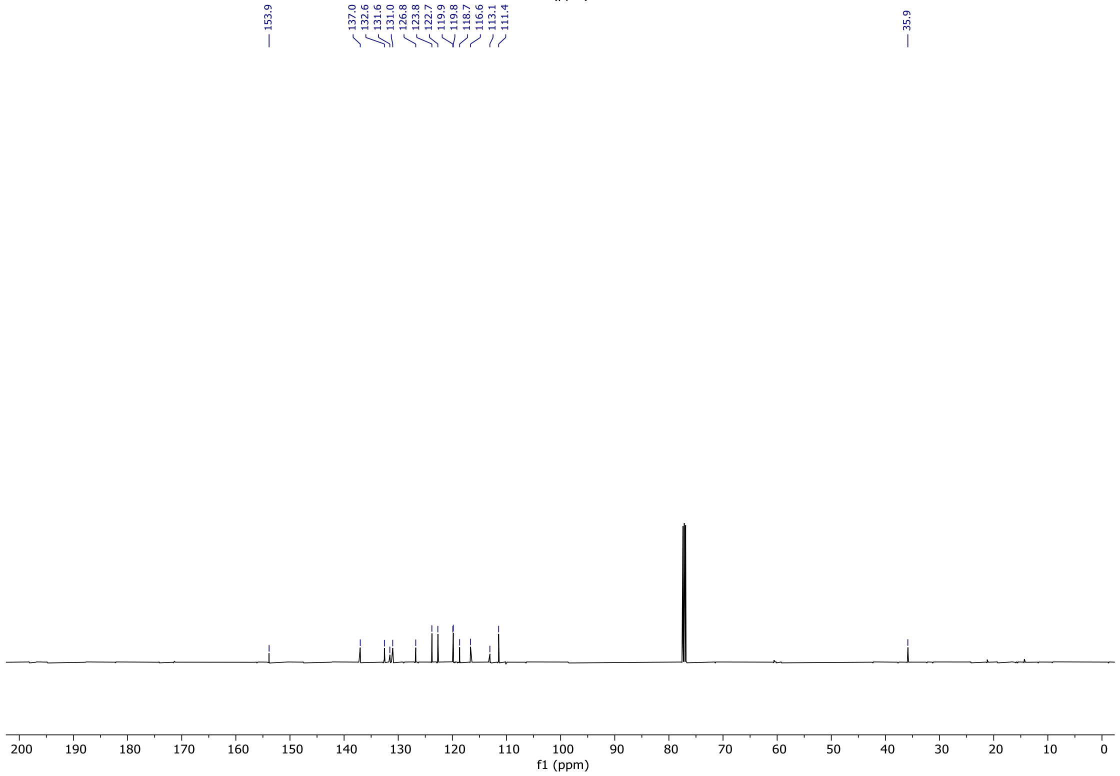
153.9
137.0
132.6
131.6
131.0
126.8
123.8
122.7
119.9
119.8
118.7
116.6
113.1
111.4
35.9
f1 (ppm)

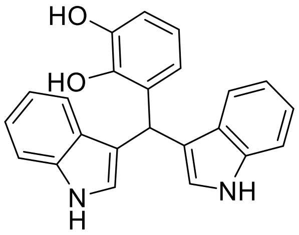

14

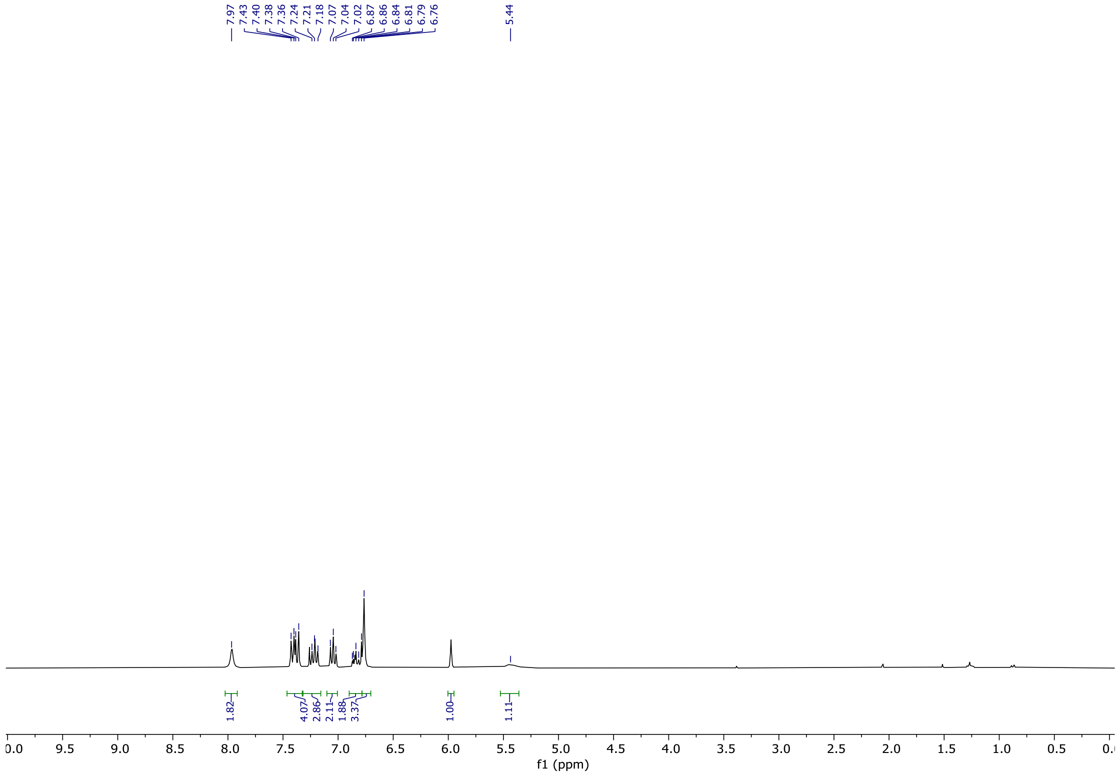
7.97
7.43
7.40
7.38
7.36
7.24
7.21
7.18
7.07
7.04
7.02
6.87
6.86
6.84
6.81
6.79
6.76
5.44
1.82
4.07
2.86
2.11
1.88
3.37
1.00
1.11
f1 (ppm)

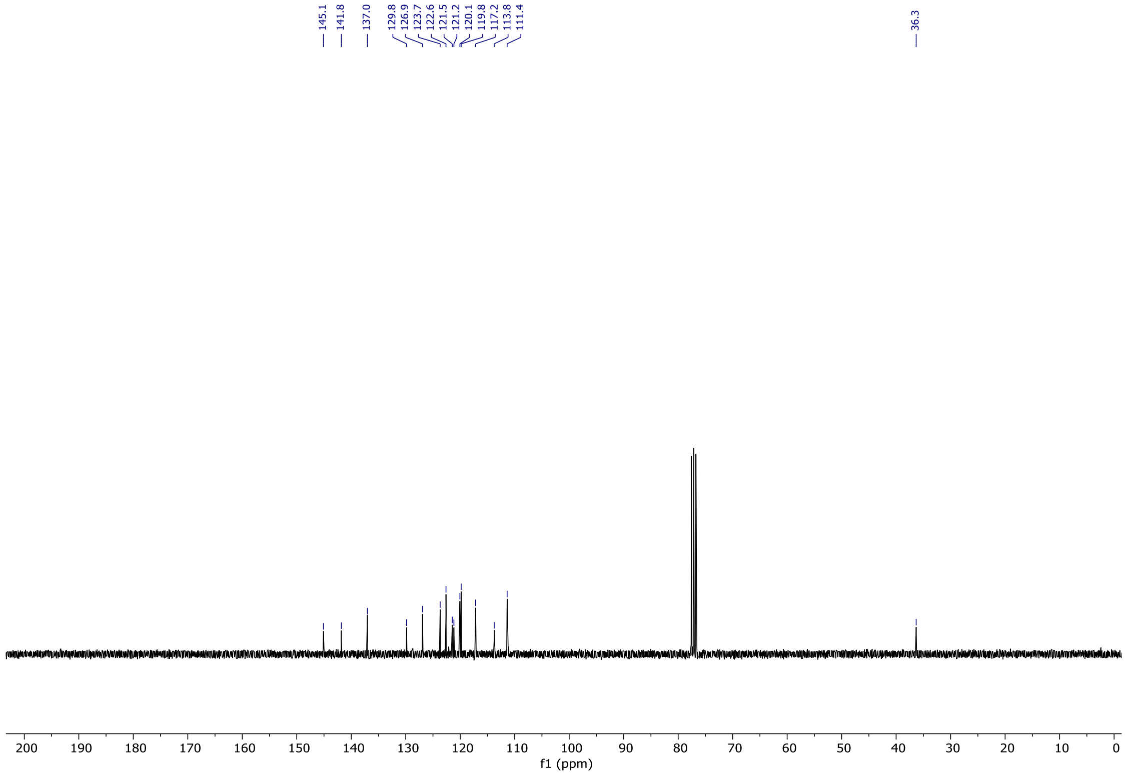
145.1
141.8
137.0
129.8
126.9
123.7
122.6
121.5
121.2
120.1
119.8
117.2
113.8
111.4
36.3
f1 (ppm)

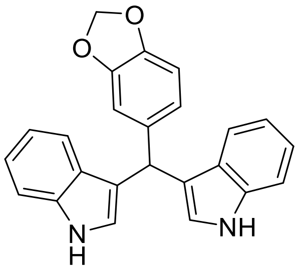
O
O
N
H
NH

15

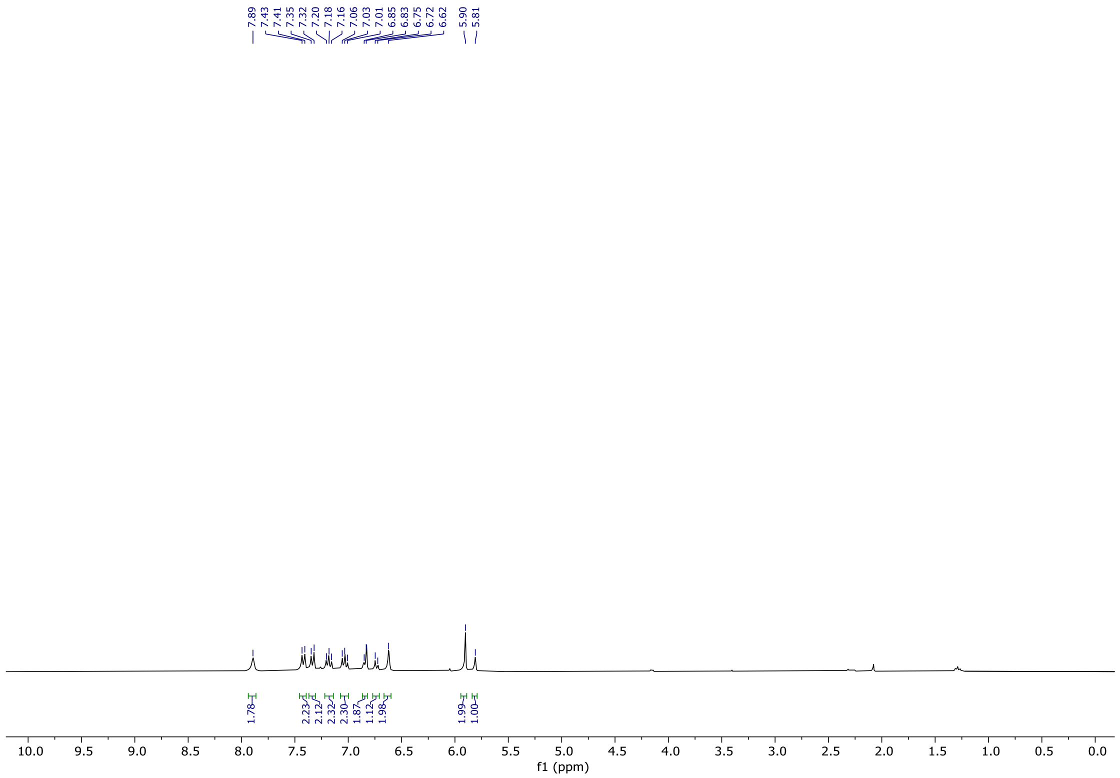
7.89
7.43
7.41
7.35
7.32
7.20
7.18
7.16
7.06
7.03
7.01
6.85
6.83
6.75
6.72
6.62
5.90
5.81
1.78
2.23
2.12
2.32
2.30
1.87
1.12
1.98
1.99
1.00
f1 (ppm)

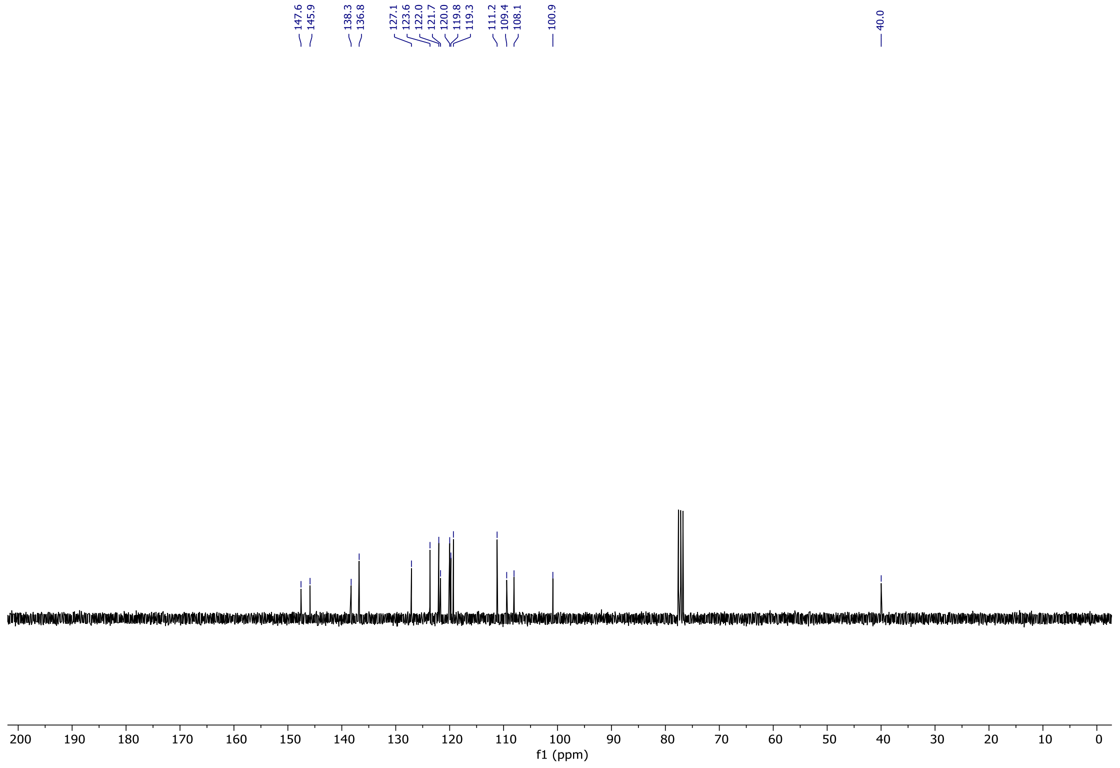
147.6
145.9
138.3
136.8
127.1
123.6
122.0
121.7
120.0
119.8
119.3
111.2
109.4
108.1
100.9
40.0
f1 (ppm)

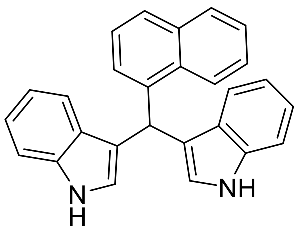
N
H
NH

16

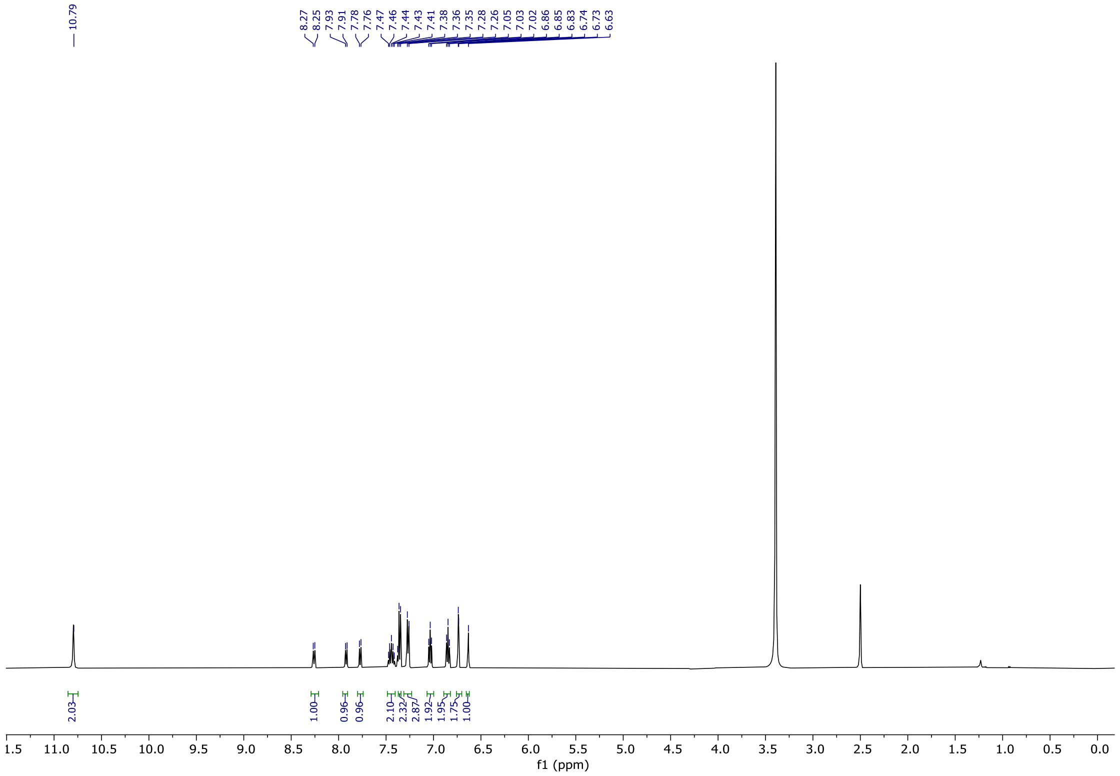
10.79
8.27
8.25
7.93
7.91
7.78
7.76
7.47
7.46
7.44
7.43
7.41
7.38
7.36
7.35
7.28
7.26
7.05
7.03
7.02
6.86
6.85
6.83
6.74
6.73
6.63
2.03
1.00
0.96
0.96
2.10
2.32
2.87
1.92
1.95
1.75
1.00
f1 (ppm)

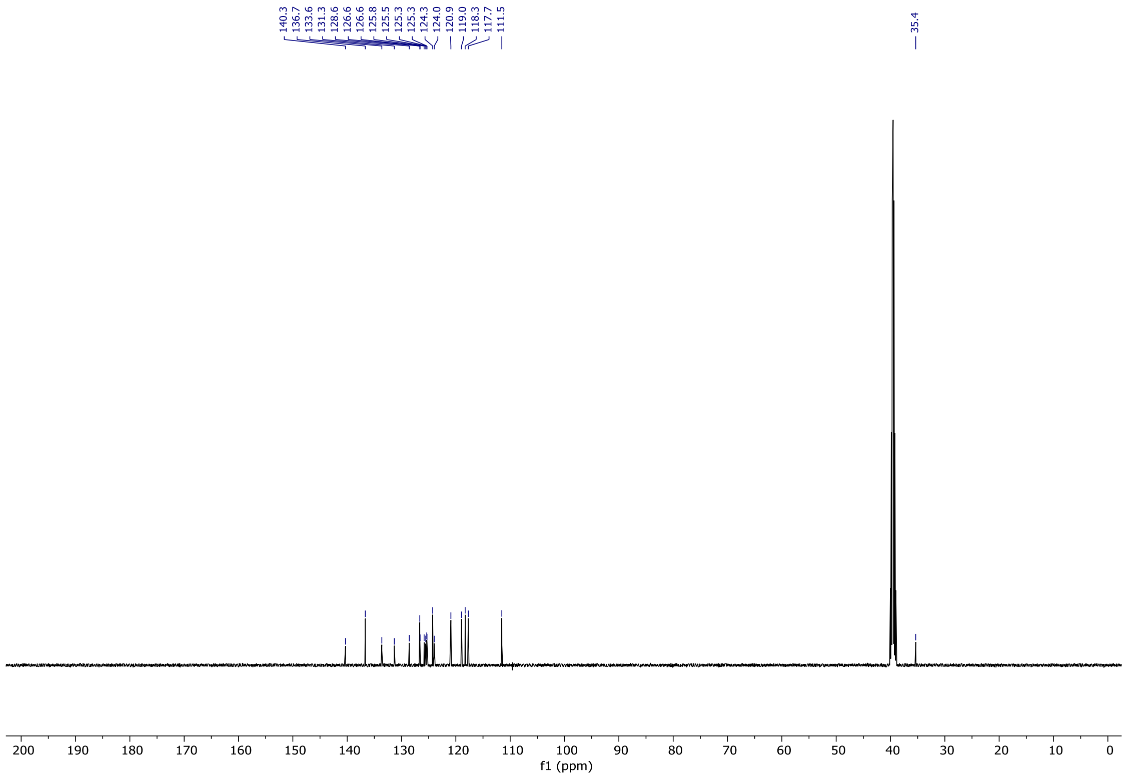
140.3
136.7
133.6
131.3
128.6
126.6
126.6
125.8
125.5
125.3
125.3
124.3
124.0
120.9
119.0
118.3
117.7
111.5
35.4
f1 (ppm)

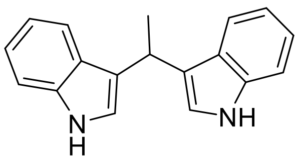

17

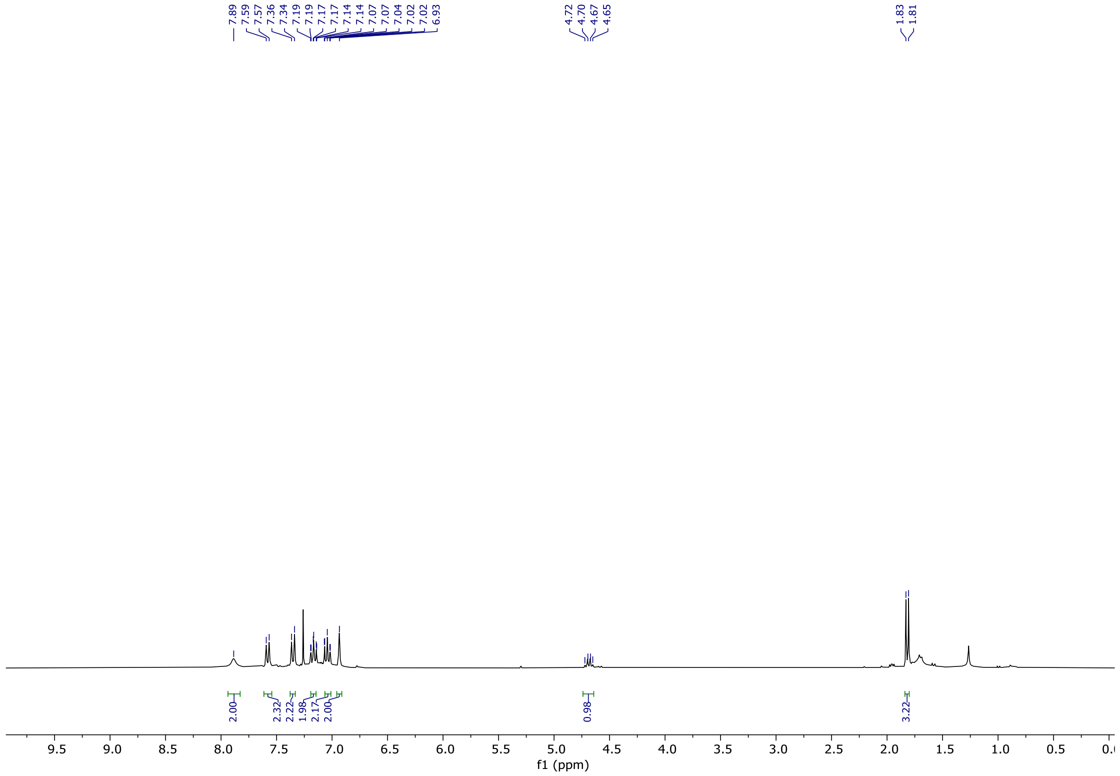
7.89
7.59
7.57
7.36
7.34
7.19
7.19
7.17
7.17
7.14
7.14
7.07
7.07
7.04
7.02
7.02
6.93
4.72
4.70
4.67
4.65
1.83
1.81
2.00
2.32
2.22
1.98
2.17
2.00
0.98
3.22
f1 (ppm)

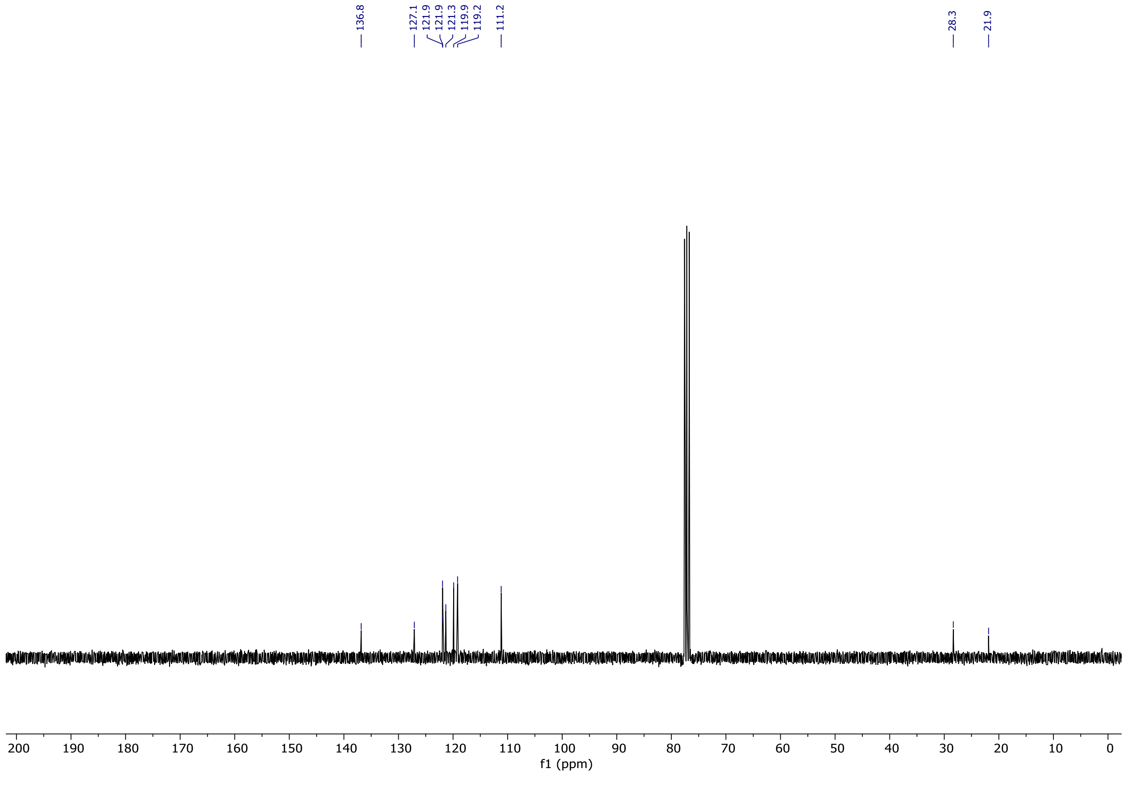
136.8
127.1
121.9
121.9
121.3
119.9
119.2
111.2
28.3
21.9
f1 (ppm)

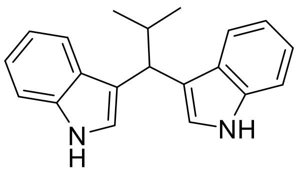

18

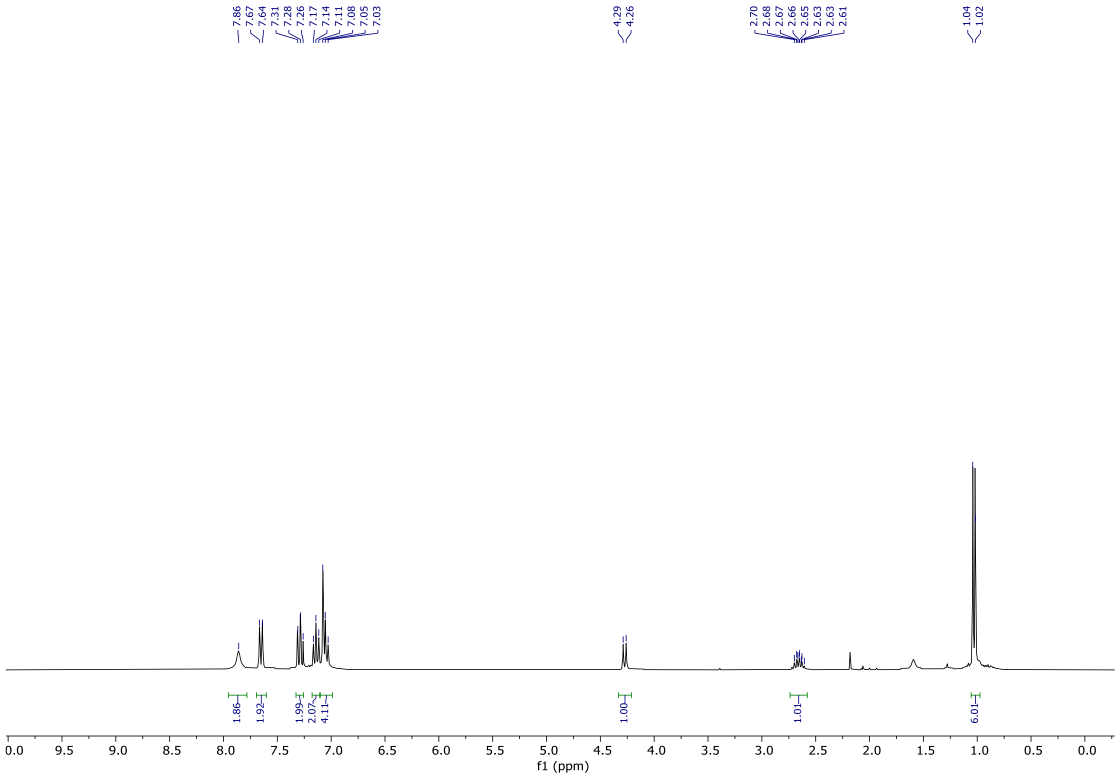
7.86
7.67
7.64
7.31
7.28
7.26
7.17
7.14
7.11
7.08
7.05
7.03
4.29
4.26
2.70
2.68
2.67
2.66
2.65
2.63
2.63
2.61
1.04
1.02
1.86
1.92
1.99
2.07
4.11
1.00
1.01
6.01
f1 (ppm)

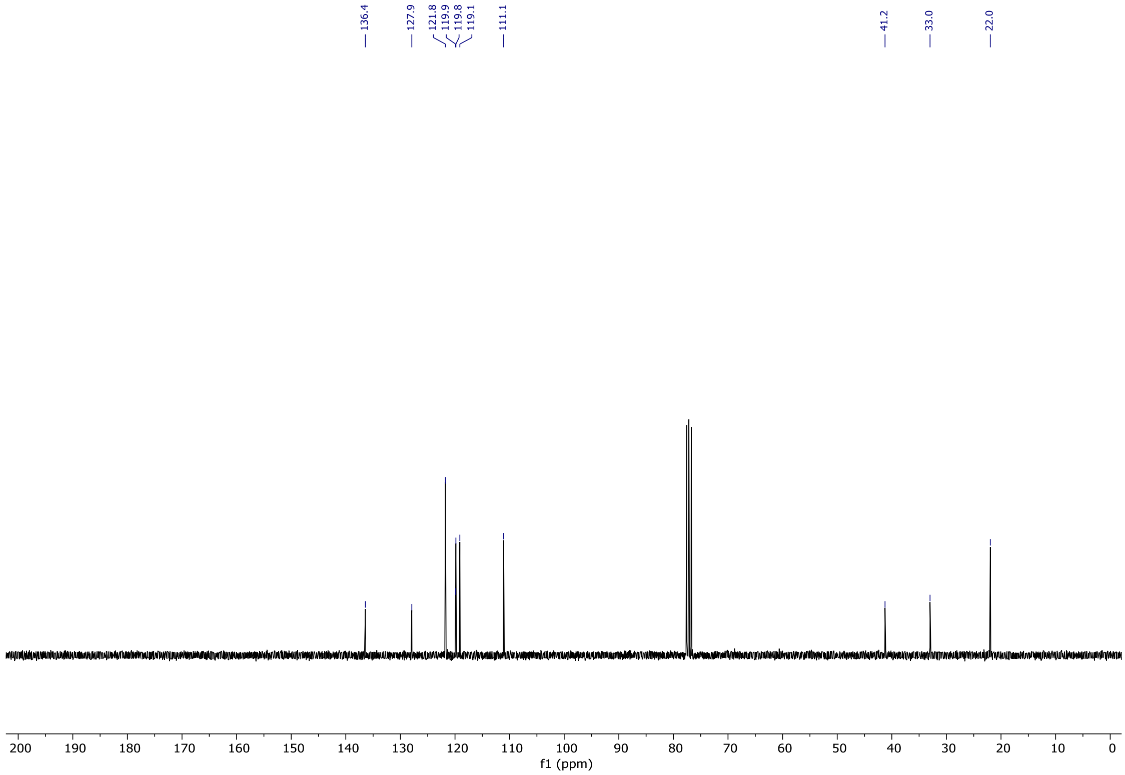
136.4
127.9
121.8
119.9
119.8
119.1
111.1
41.2
33.0
22.0
f1 (ppm)

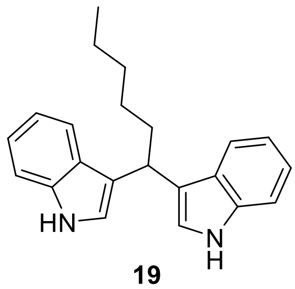
HN
N
H

19

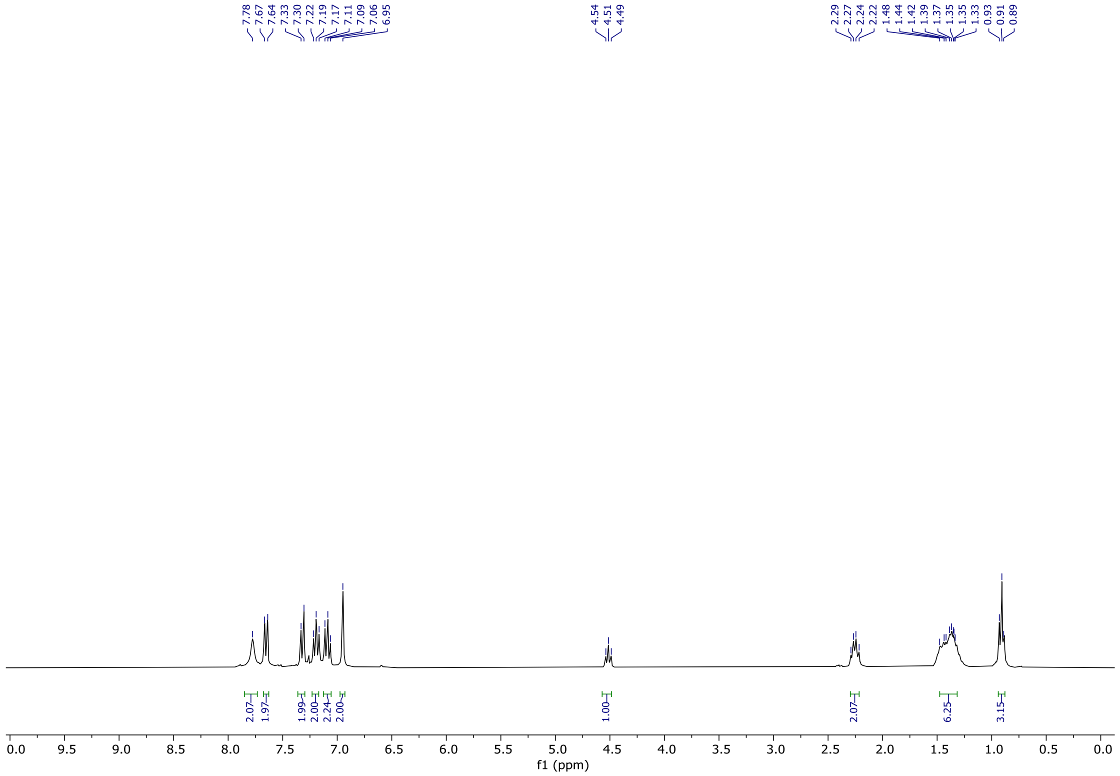
7.78
7.67
7.64
7.33
7.30
7.22
7.19
7.17
7.11
7.09
7.06
6.95
4.54
4.51
4.49
2.29
2.27
2.24
2.22
1.48
1.44
1.42
1.39
1.37
1.35
1.35
1.33
0.93
0.91
0.89
2.07
1.97
1.99
2.00
2.24
2.00
1.00
2.07
6.25
3.15
f1 (ppm)

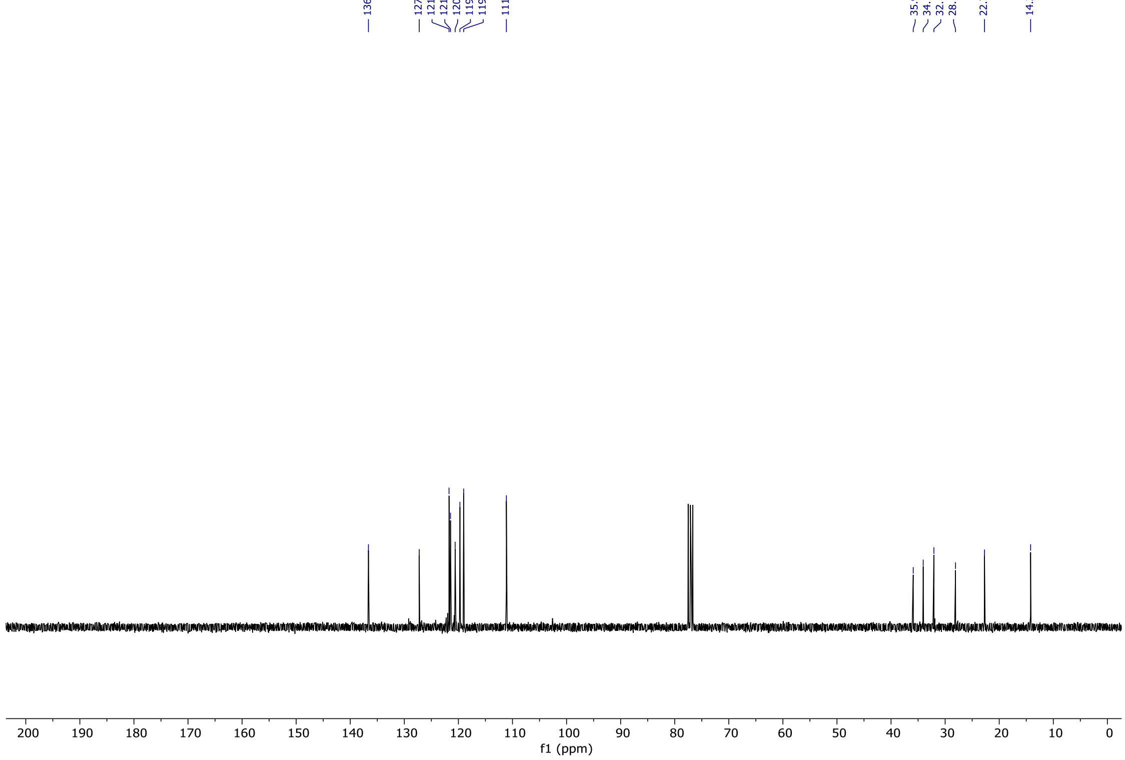
136.6
127.2
121.7
121.5
120.6
119.7
119.0
111.1
35.9
34.1
32.1
28.1
22.7
14.2
f1 (ppm)

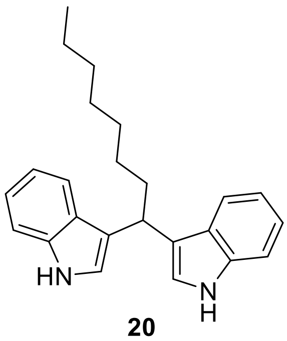
HN
N
H

20

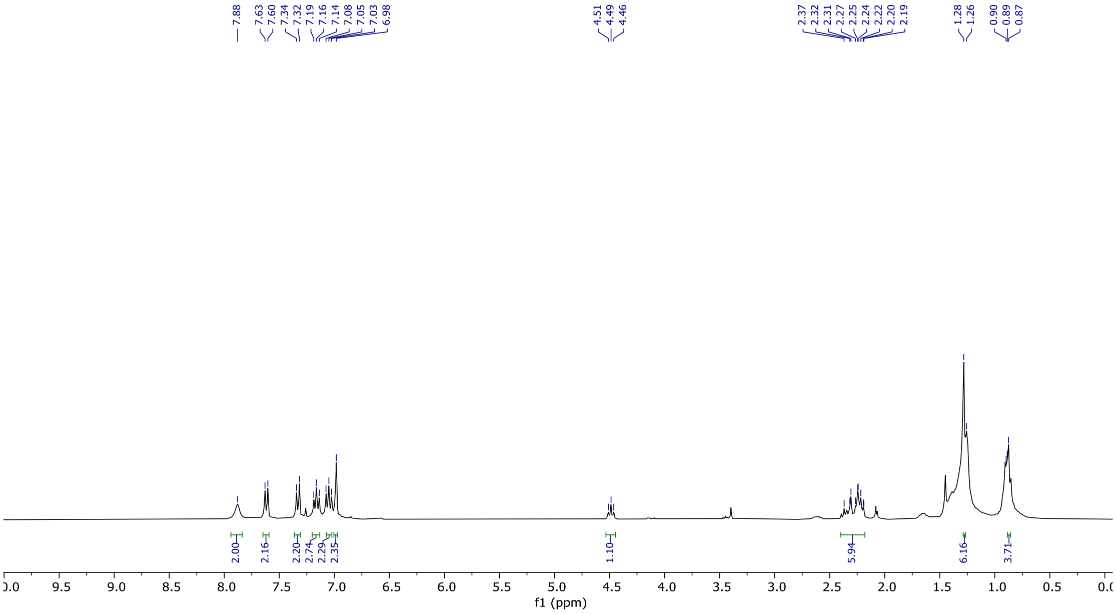
7.88
7.63
7.60
7.34
7.32
7.19
7.16
7.14
7.08
7.05
7.03
6.98
4.51
4.49
4.46
2.37
2.32
2.31
2.27
2.25
2.24
2.22
2.20
2.19
1.28
1.26
0.90
0.89
0.87
2.00
2.16
2.20
2.74
2.29
2.35
1.10
5.94
6.16
3.71
f1 (ppm)

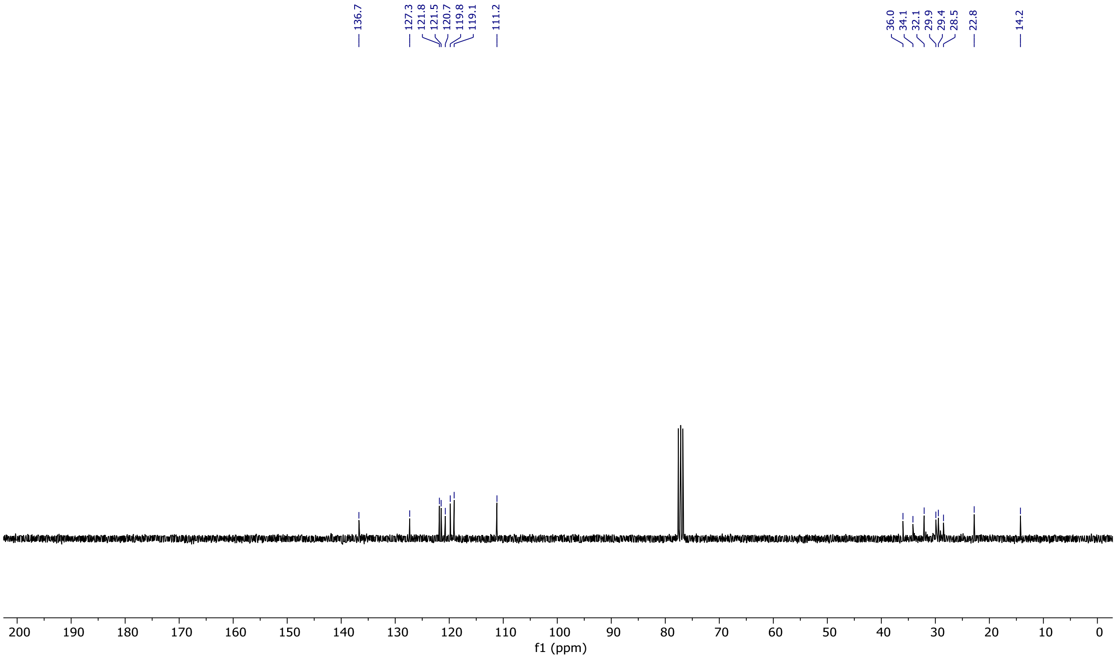
136.7
127.3
121.8
121.5
120.7
119.8
119.1
111.2
36.0
34.1
32.1
29.9
29.4
28.5
22.8
14.2
f1 (ppm)

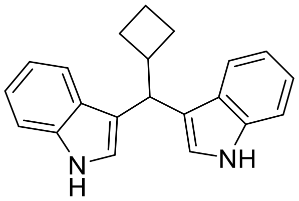
N
H
NH

21

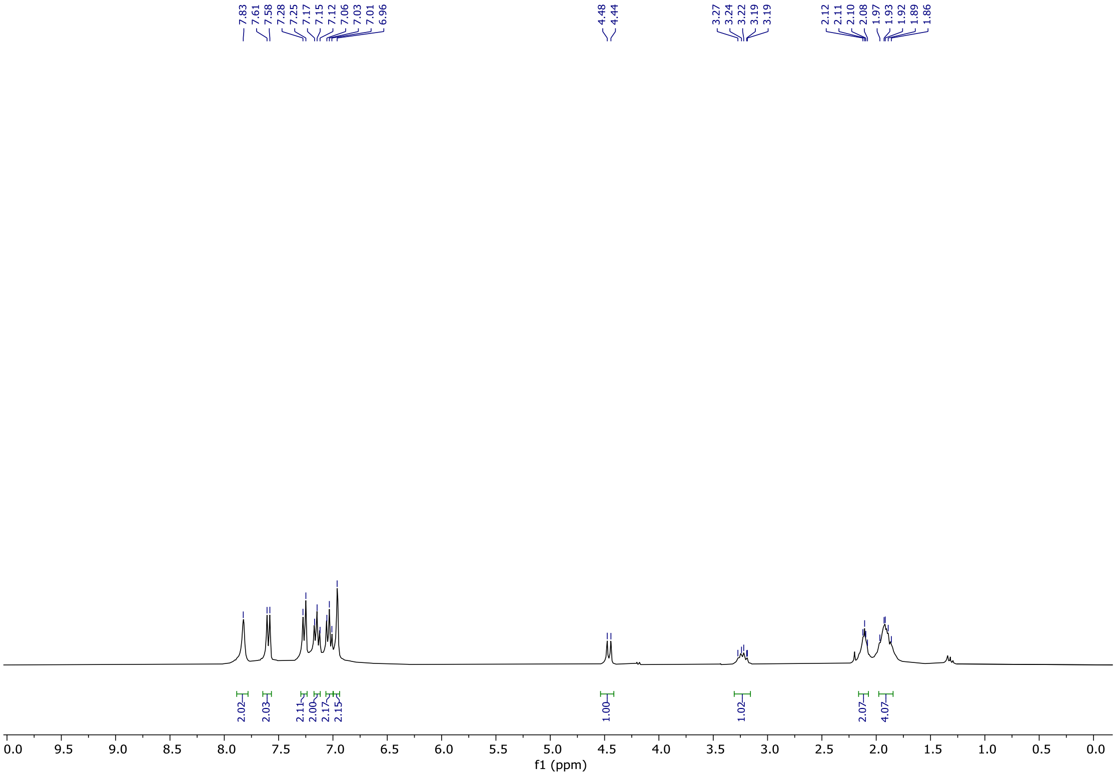
7.83
7.61
7.58
7.28
7.25
7.17
7.15
7.12
7.06
7.03
7.01
6.96
4.48
4.44
3.27
3.24
3.22
3.19
3.19
2.12
2.11
2.10
2.08
1.97
1.93
1.92
1.89
1.86
2.02
2.03
2.11
2.00
2.17
2.15
1.00
1.02
2.07
4.07
f1 (ppm)

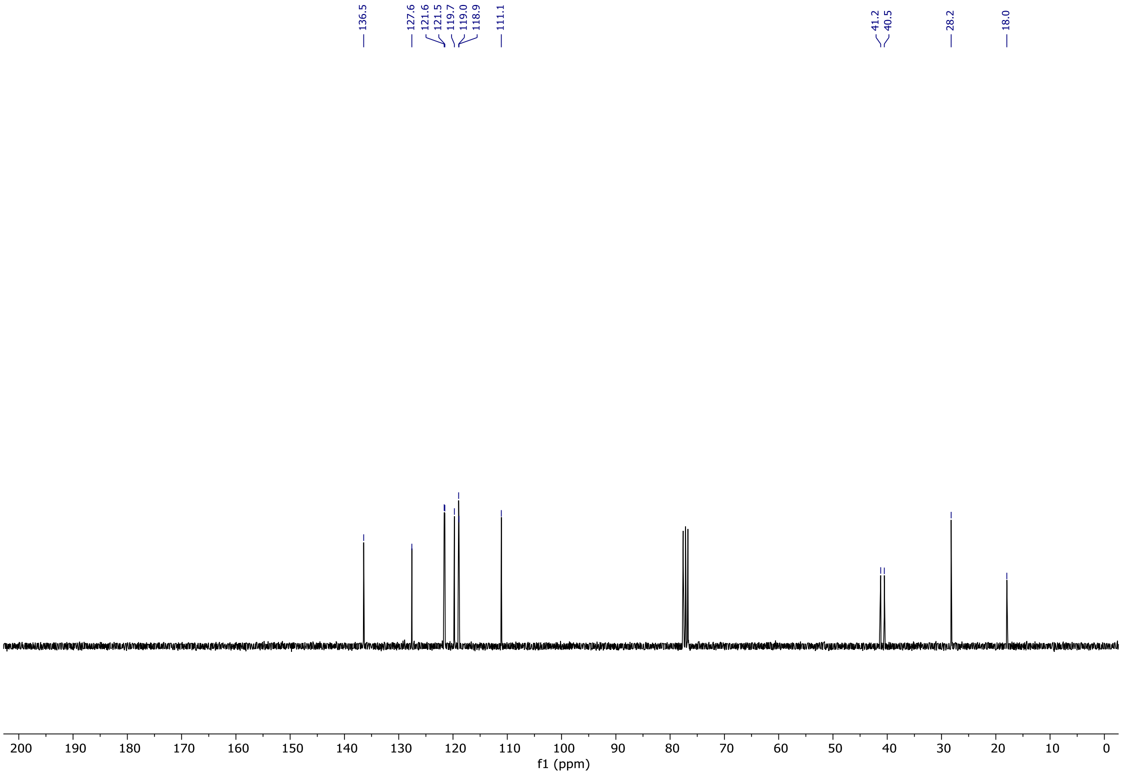
136.5
127.6
121.6
121.5
119.7
119.0
118.9
111.1
41.2
40.5
28.2
18.0
f1 (ppm)

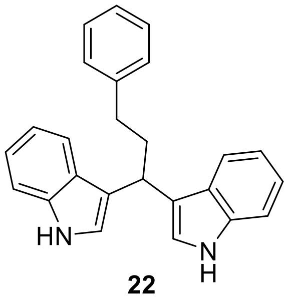
HN
N
H

22

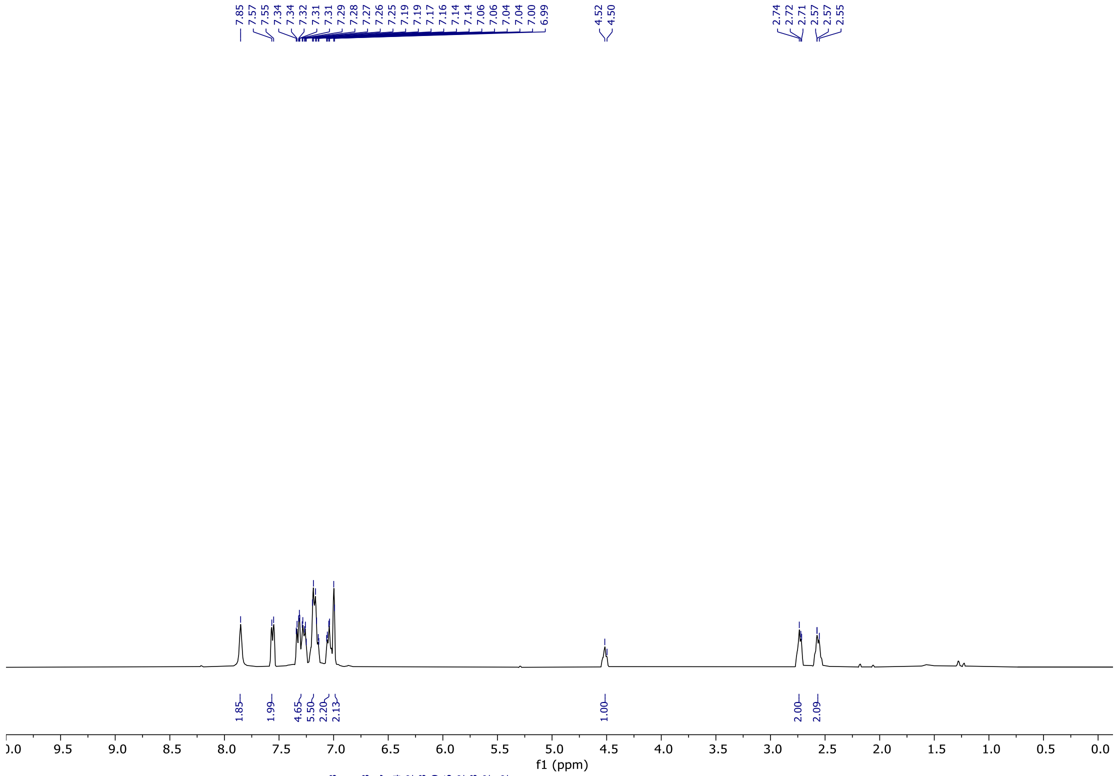
7.85
7.57
7.55
7.34
7.32
7.31
7.31
7.29
7.28
7.27
7.26
7.25
7.19
7.19
7.17
7.16
7.14
7.14
7.06
7.06
7.04
7.04
7.00
6.99
4.52
4.50
2.74
2.72
2.71
2.57
2.57
2.55
1.85
1.99
4.65
5.50
2.20
2.13
1.00
2.00
2.09
f1 (ppm)

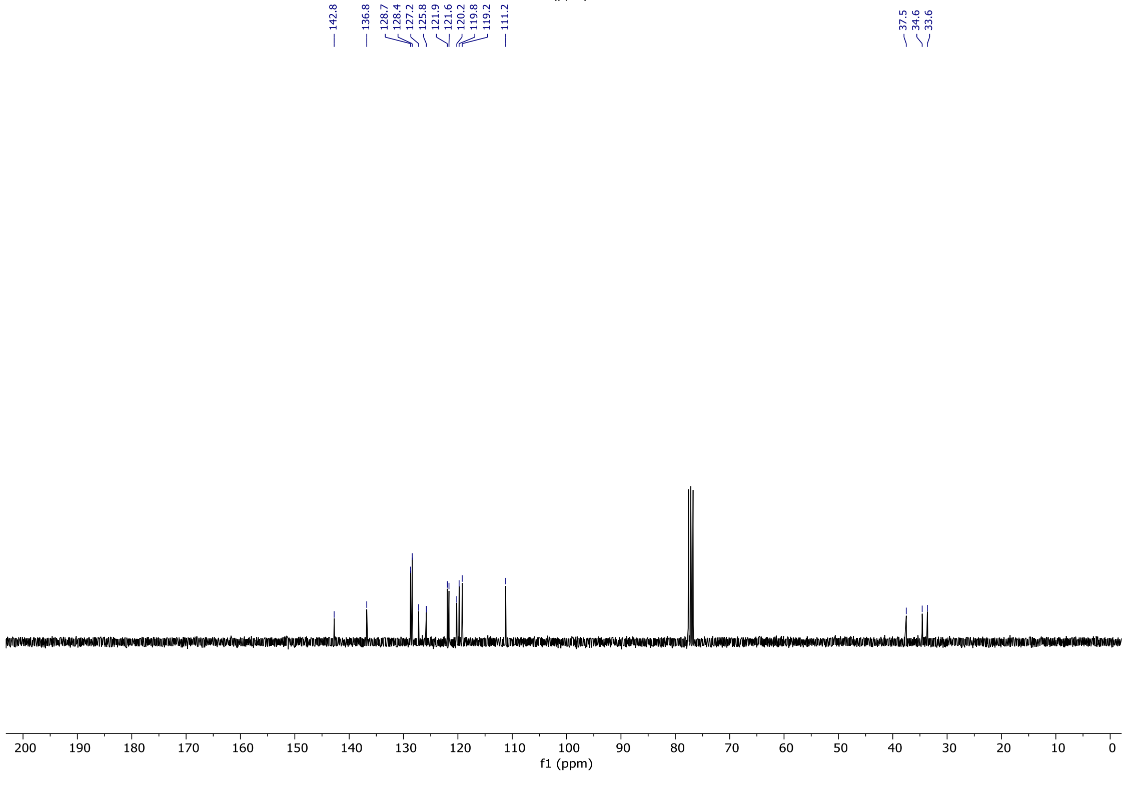
142.8
136.8
128.7
128.4
127.2
125.8
121.9
121.6
120.2
119.8
119.2
111.2
37.5
34.6
33.6
f1 (ppm)

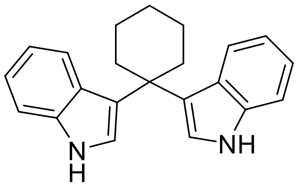
NH
H
NH

23

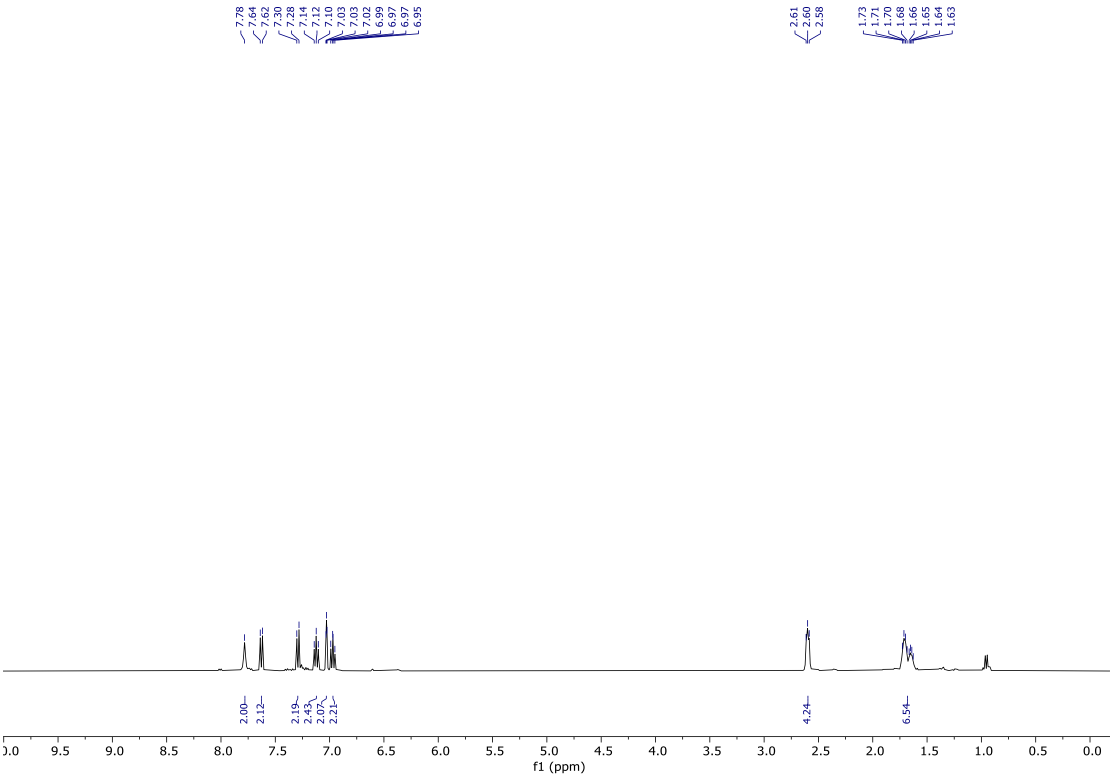
7.78
7.64
7.62
7.30
7.28
7.14
7.12
7.10
7.03
7.03
7.02
6.99
6.97
6.97
6.95
2.61
2.60
2.58
1.73
1.71
1.70
1.68
1.66
1.65
1.64
1.63
2.00
2.12
2.19
2.43
2.07
2.21
4.24
6.54
f1 (ppm)

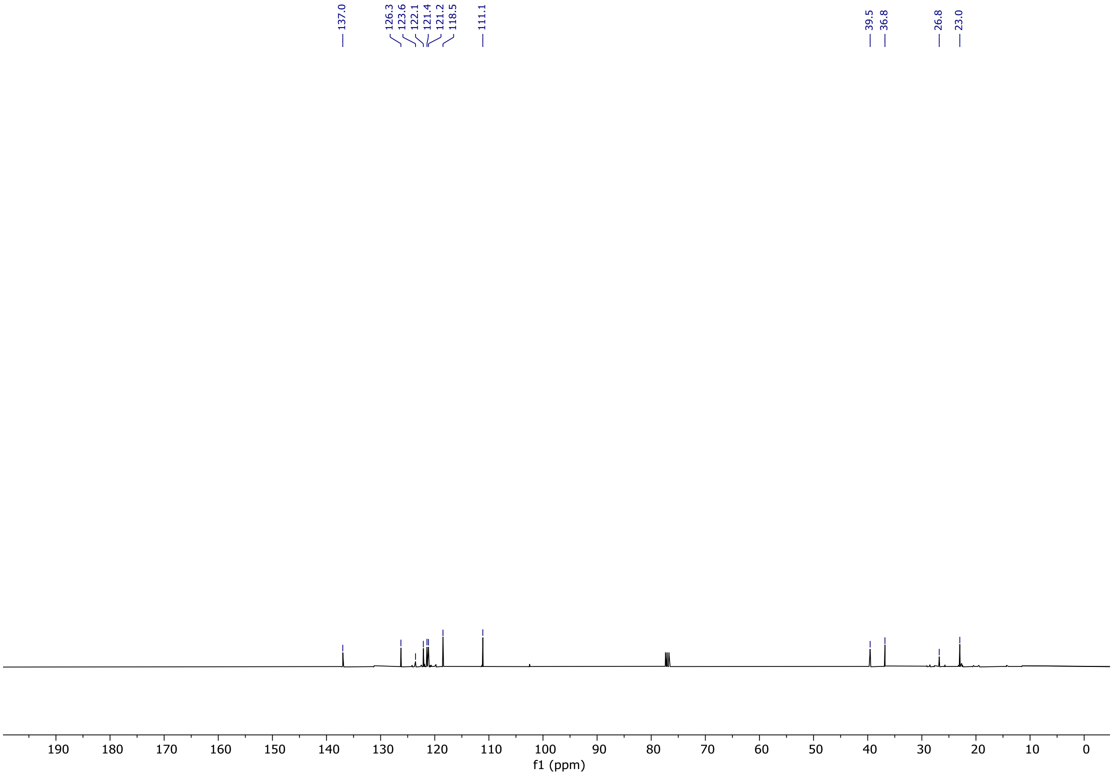
137.0
126.3
123.6
122.1
121.4
121.2
118.5
111.1
39.5
36.8
26.8
23.0
f1 (ppm)

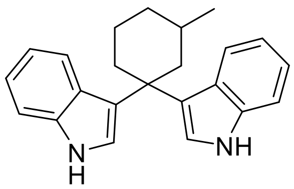
NH
N
H

24

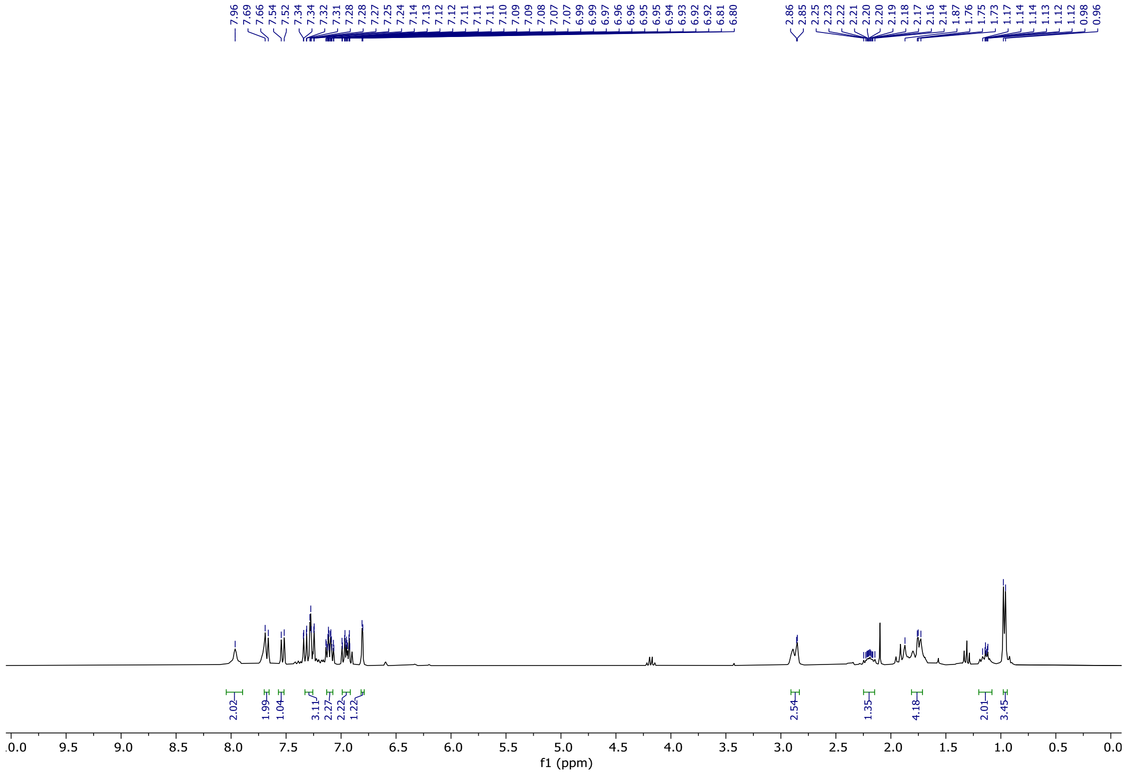
7.96 7.69 7.66 7.54 7.52 7.34 7.34 7.32 7.31 7.28 7.28 7.27 7.25 7.24 7.14 7.13 7.12 7.12 7.11 7.11 7.11 7.10 7.09 7.09 7.08 7.07 7.07 6.99 6.99 6.97 6.96 6.96 6.95 6.95 6.94 6.93 6.92 6.92 6.81 6.80
2.86 2.85 2.25 2.23 2.22 2.21 2.20 2.20 2.19 2.18 2.17 2.16 2.14 1.87 1.76 1.75 1.73 1.17 1.14 1.14 1.13 1.12 0.98 0.96
2.02 1.99 1.04 3.11 2.27 2.22 1.22 2.54 1.35 4.18 2.01 3.45
f1 (ppm)

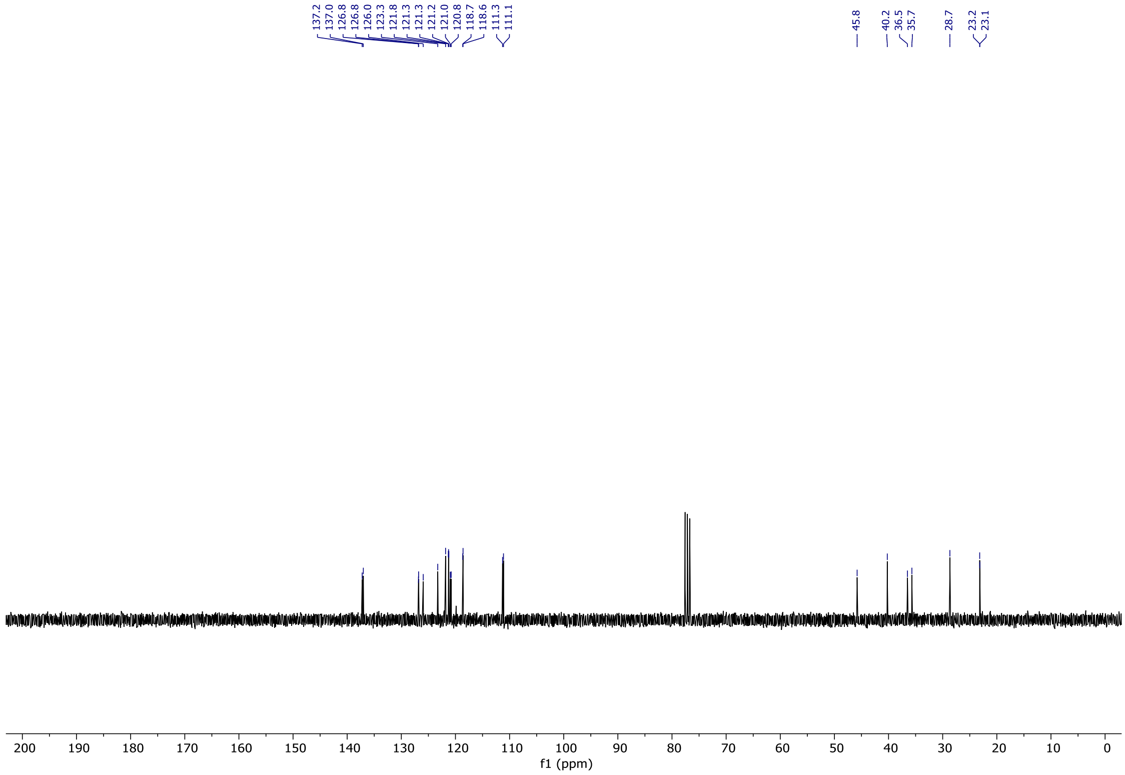
137.2 137.0 126.8 126.8 126.0 123.3 121.8 121.3 121.2 121.0 120.8 118.7 118.6 111.3 111.1
45.8 40.2 36.5 35.7 28.7 23.2 23.1
f1 (ppm)

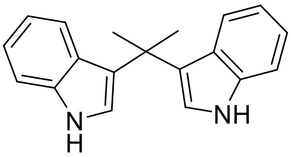
NH
N
H

25

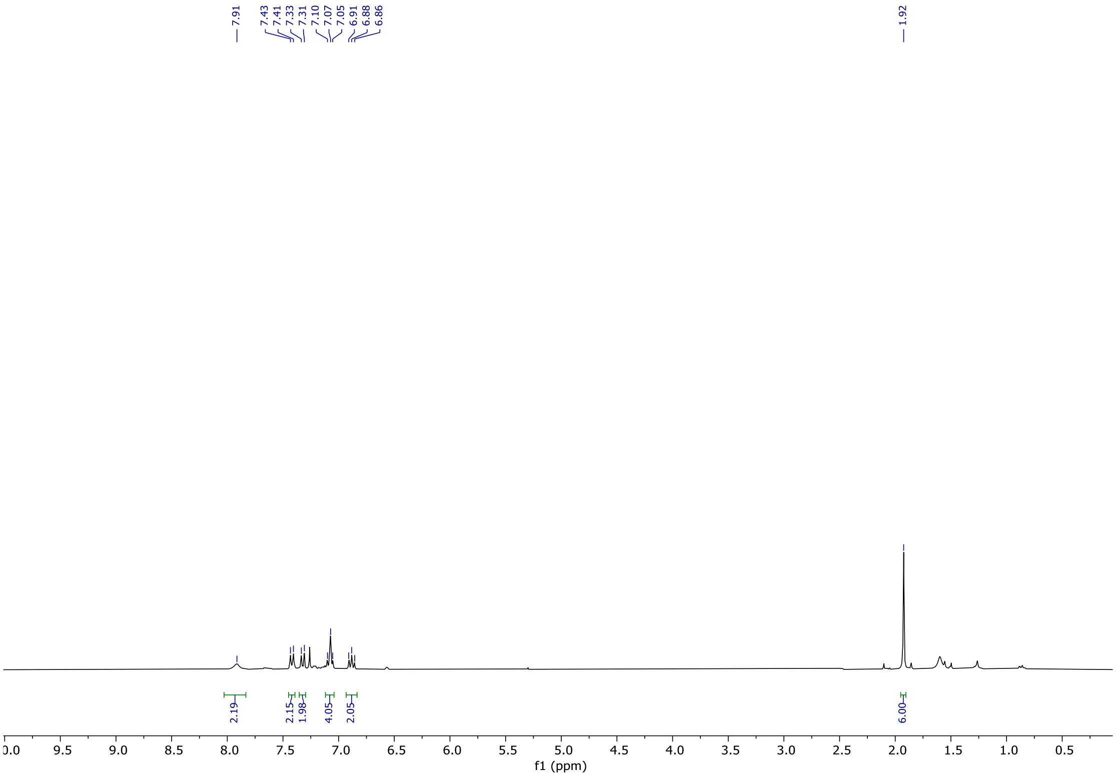
7.91
7.43
7.41
7.33
7.31
7.10
7.07
7.05
6.91
6.88
6.86
1.92
2.19
2.15
1.98
4.05
2.05
6.00
f1 (ppm)

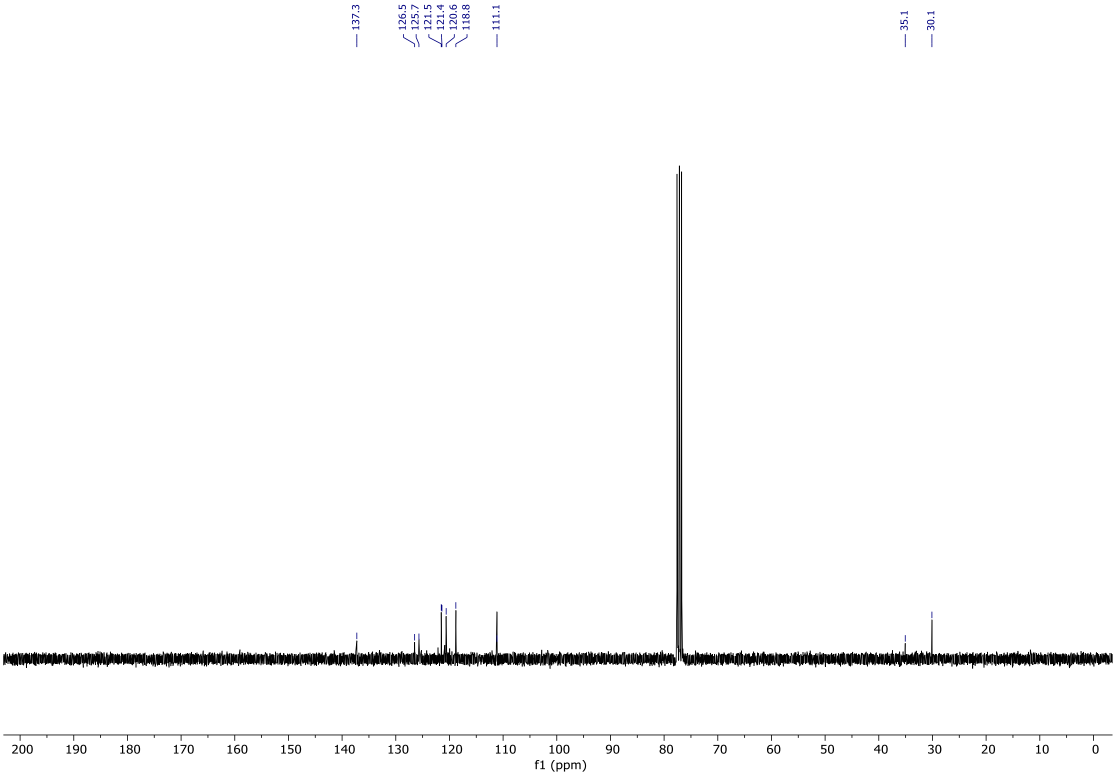
137.3
126.5
125.7
121.5
121.4
120.6
118.8
111.1
35.1
30.1
f1 (ppm)

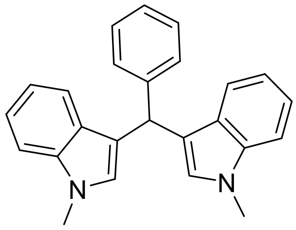
N
N

26

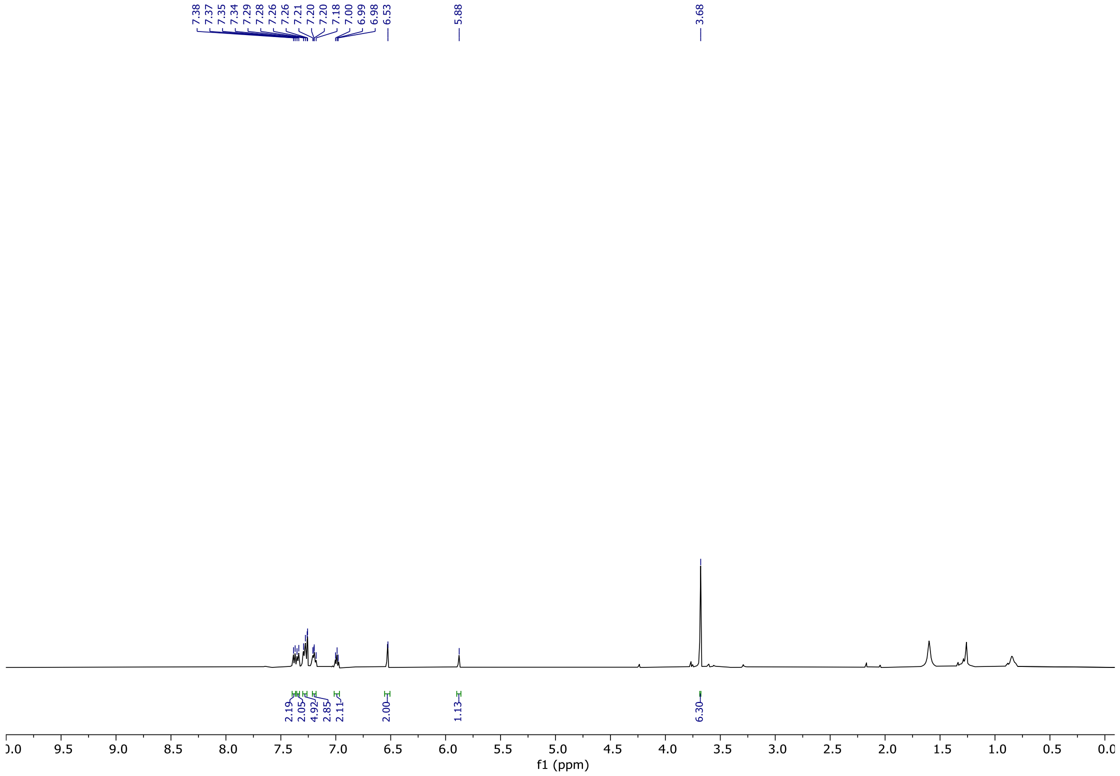
7.38
7.37
7.35
7.34
7.29
7.28
7.26
7.26
7.21
7.20
7.20
7.18
7.00
6.99
6.98
6.53
5.88
3.68
2.19
2.05
4.92
2.85
2.11
2.00
1.13
6.30
f1 (ppm)

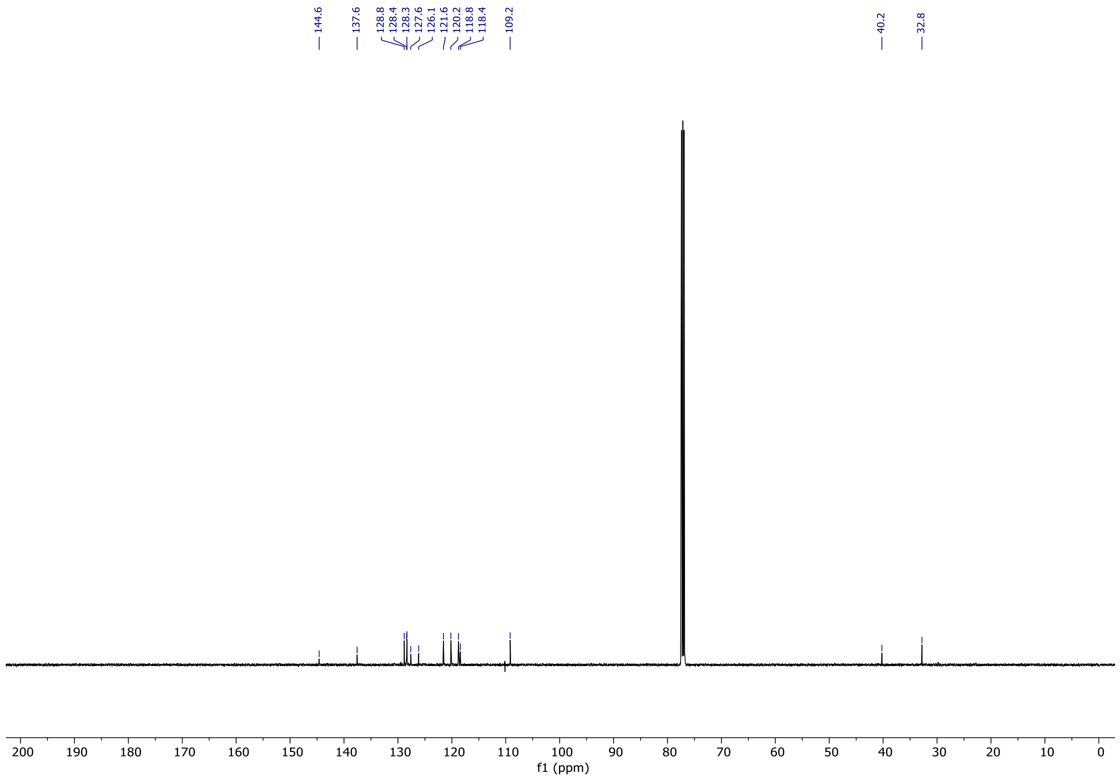
144.6
137.6
128.8
128.4
128.3
127.6
126.1
121.6
120.2
118.8
118.4
109.2
40.2
32.8
f1 (ppm)

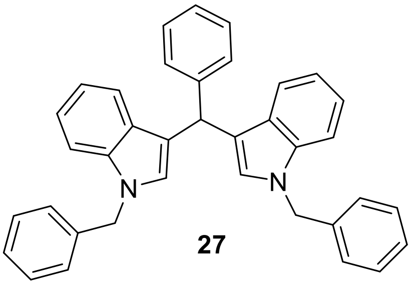

27

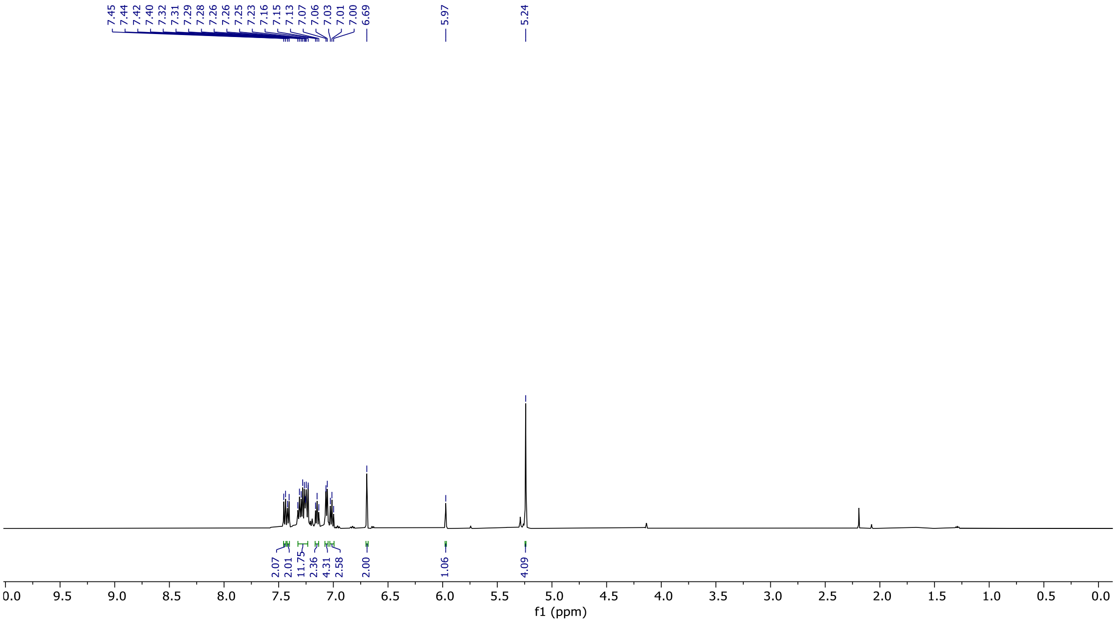
7.45
7.44
7.42
7.40
7.32
7.31
7.29
7.28
7.26
7.26
7.25
7.23
7.16
7.15
7.13
7.07
7.06
7.03
7.01
7.00
6.69
5.97
5.24
2.07
2.01
11.75
2.36
4.31
2.58
2.00
1.06
4.09
f1 (ppm)

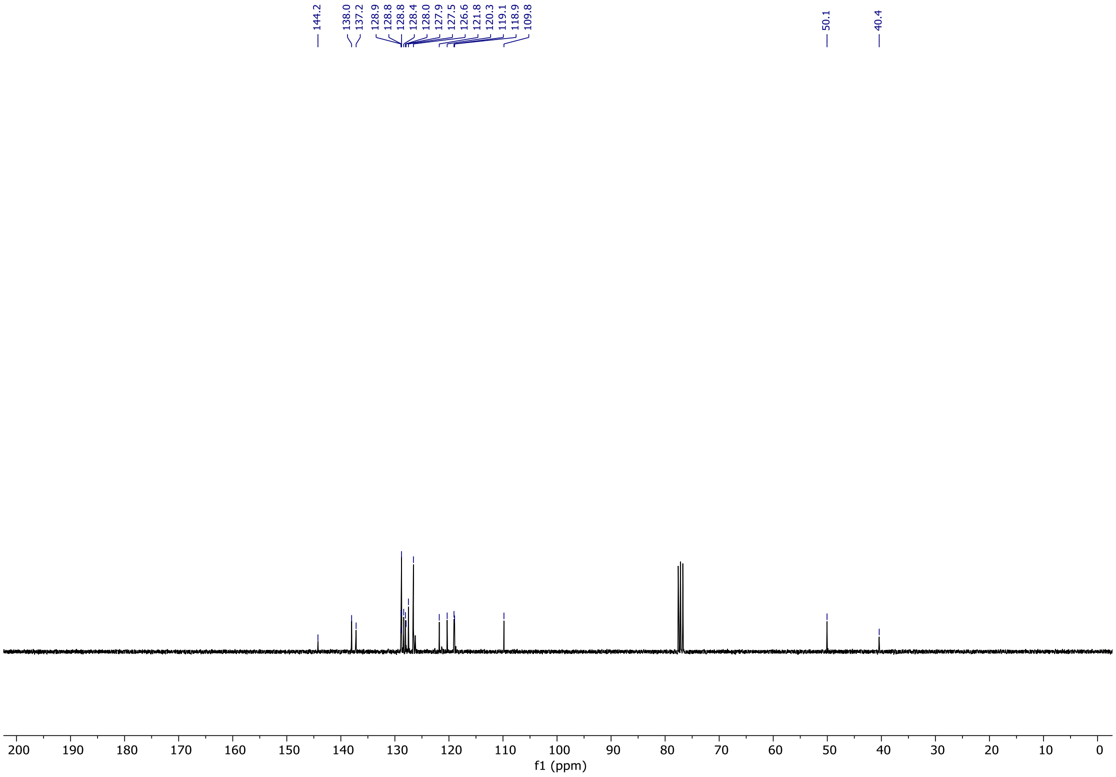
144.2
138.0
137.2
128.9
128.8
128.8
128.4
128.0
127.9
127.5
126.6
121.8
120.3
119.1
118.9
109.8
50.1
40.4
f1 (ppm)

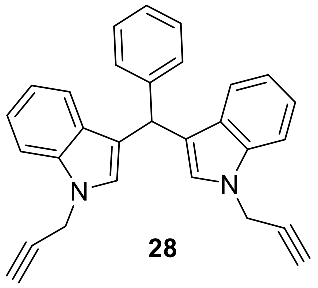

28

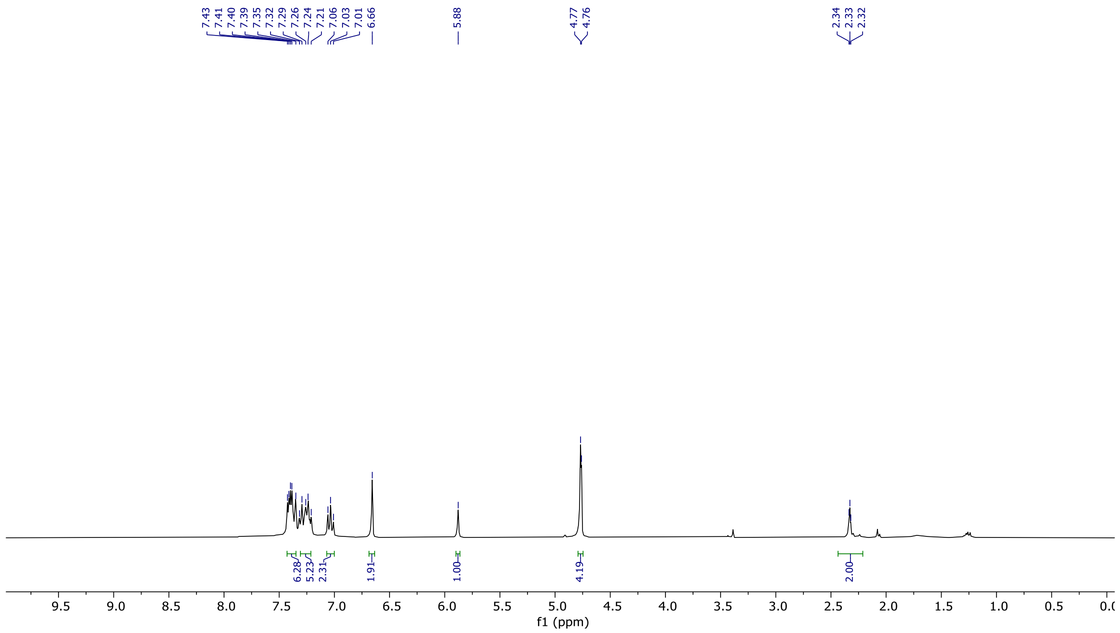
7.43
7.41
7.40
7.39
7.35
7.32
7.29
7.26
7.24
7.21
7.06
7.03
7.01
6.66
5.88
4.77
4.76
2.34
2.33
2.32
6.28
5.23
2.31
1.91
1.00
4.19
2.00
f1 (ppm)

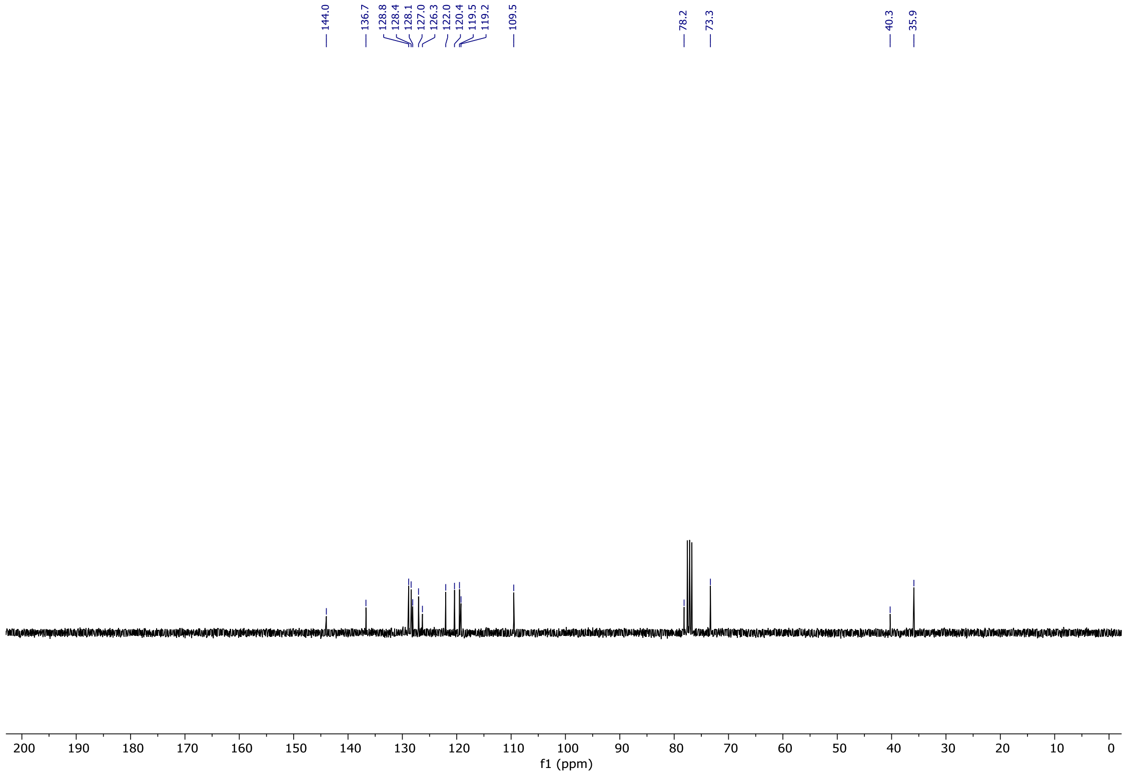
144.0
136.7
128.8
128.4
128.1
127.0
126.3
122.0
120.4
119.5
119.2
109.5
78.2
73.3
40.3
35.9
f1 (ppm)

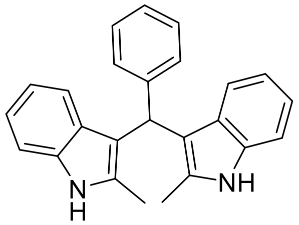
N
H
NH

29

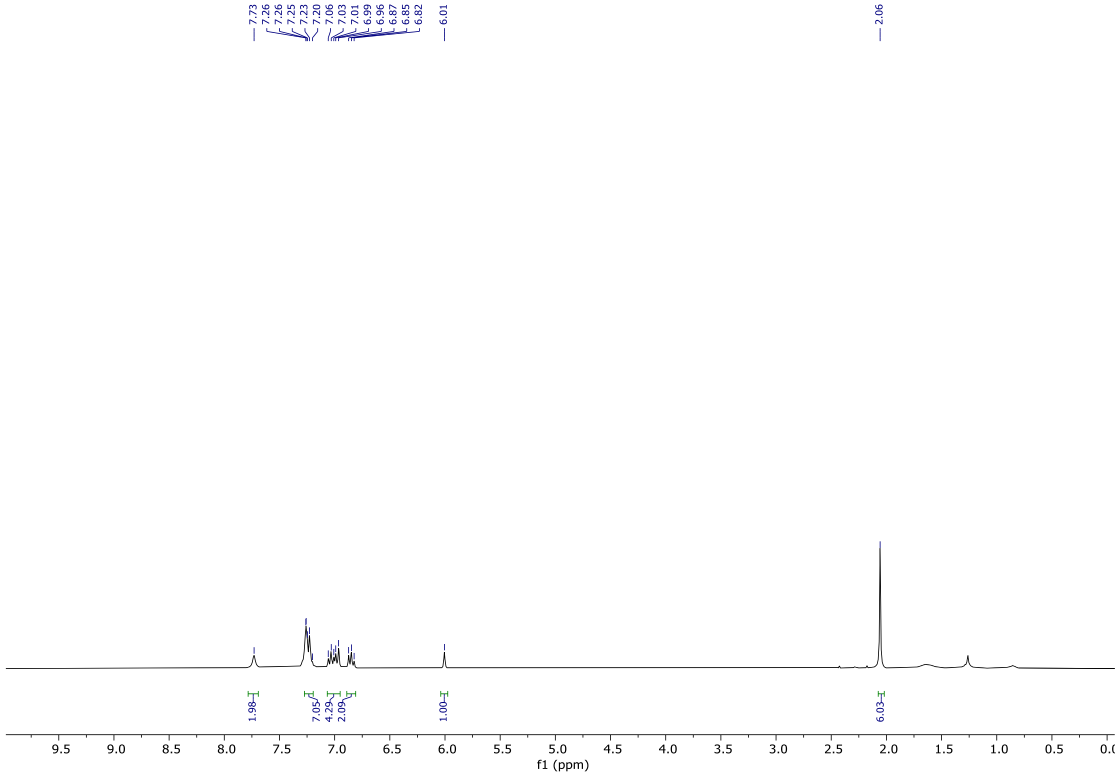
7.73
7.26
7.26
7.25
7.23
7.20
7.06
7.03
7.01
6.99
6.96
6.87
6.85
6.82
6.01
2.06
1.98
7.05
4.29
2.09
1.00
6.03
9.5 9.0 8.5 8.0 7.5 7.0 6.5 6.0 5.5 5.0 4.5 4.0 3.5 3.0 2.5 2.0 1.5 1.0 0.5
f1 (ppm)

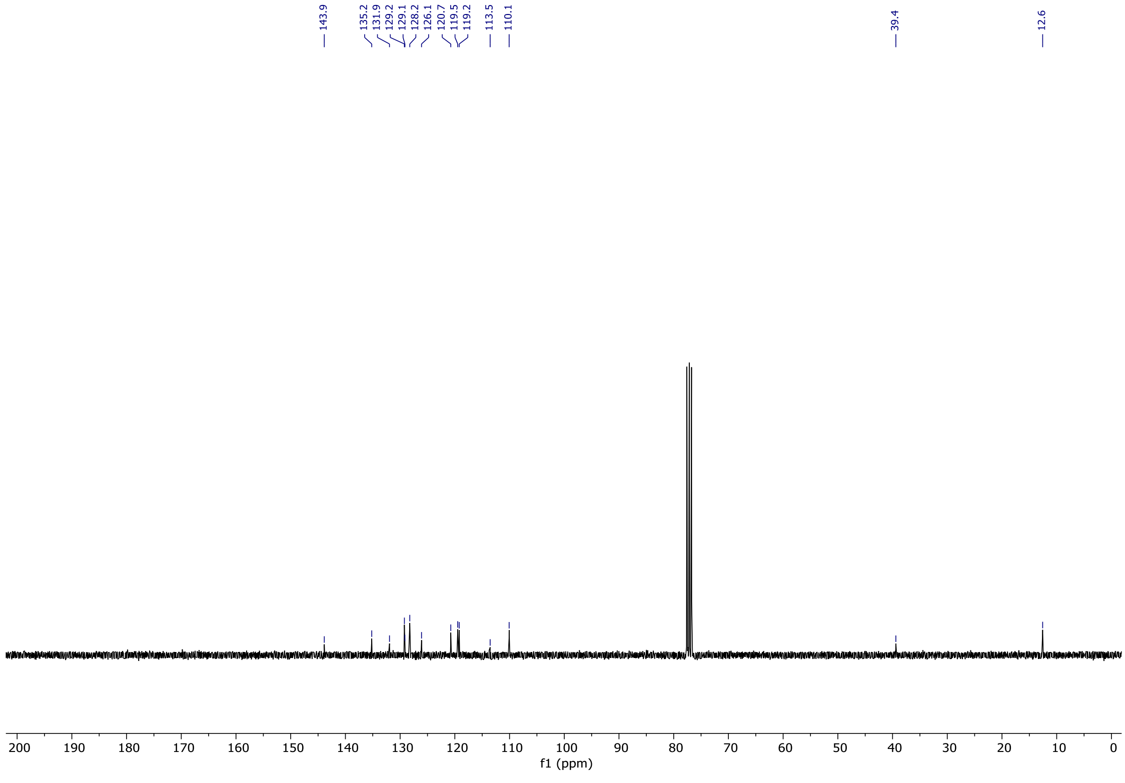
143.9
135.2
131.9
129.2
129.1
128.2
126.1
120.7
119.5
119.2
113.5
110.1
39.4
12.6
200 190 180 170 160 150 140 130 120 110 100 90 80 70 60 50 40 30 20 10 0
f1 (ppm)

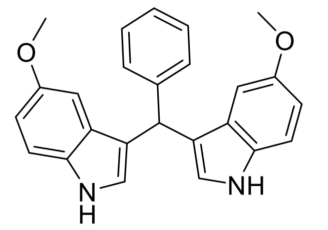
O
O
N
H
NH

30

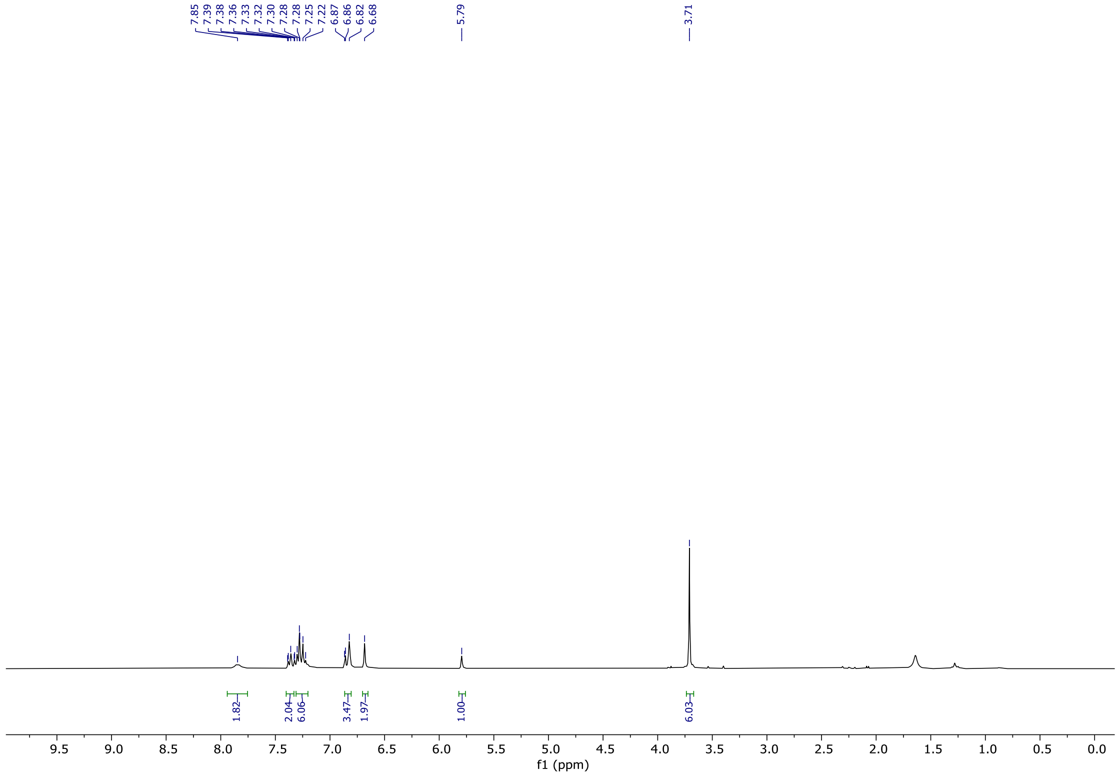
7.85
7.39
7.38
7.36
7.33
7.32
7.30
7.28
7.28
7.25
7.22
6.87
6.86
6.82
6.68
5.79
3.71
1.82
2.04
6.06
3.47
1.97
1.00
6.03
f1 (ppm)

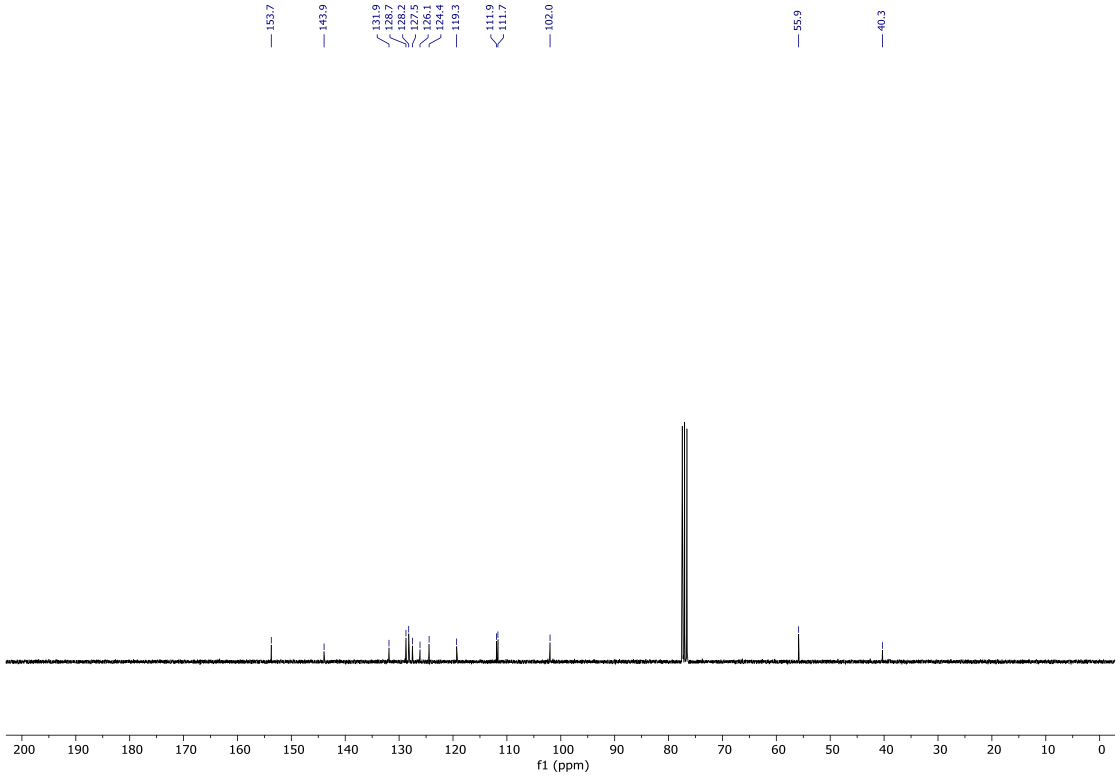
153.7
143.9
131.9
128.7
128.2
127.5
126.1
124.4
119.3
111.9
111.7
102.0
55.9
40.3
f1 (ppm)

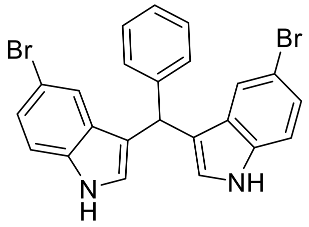
Br
Br
N
H
NH

31

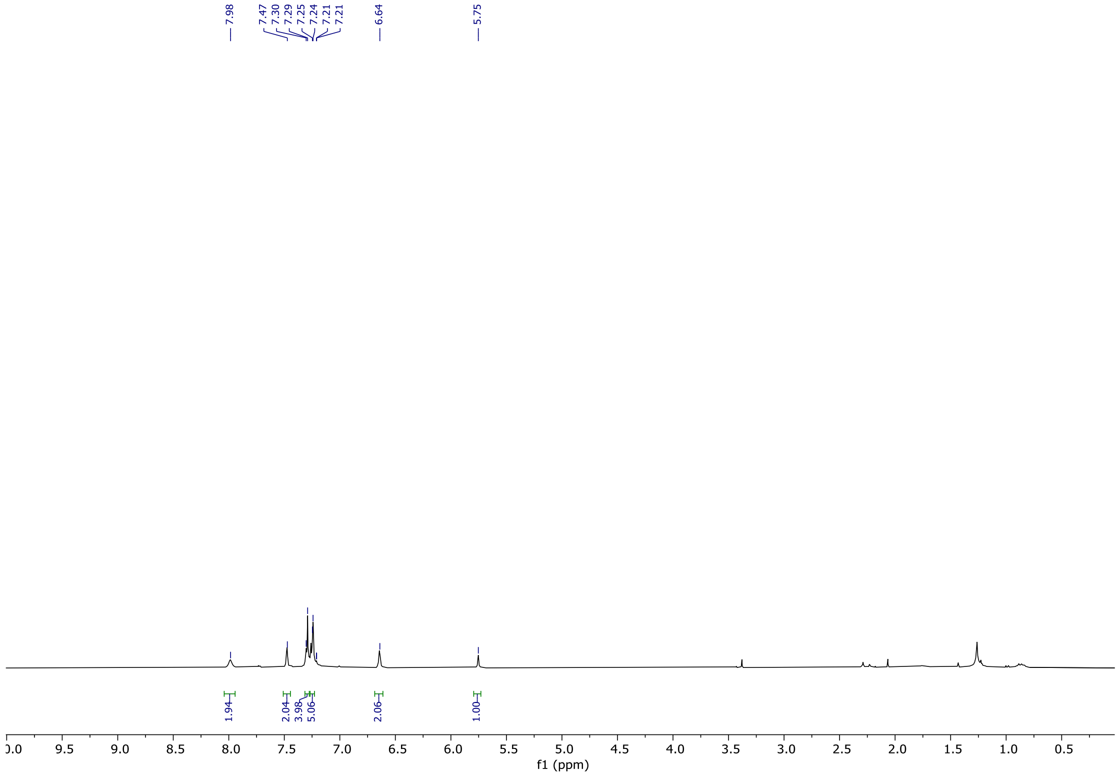
7.98
7.47
7.30
7.29
7.25
7.24
7.21
7.21
6.64
5.75
1.94
2.04
3.98
5.06
2.06
1.00
f1 (ppm)

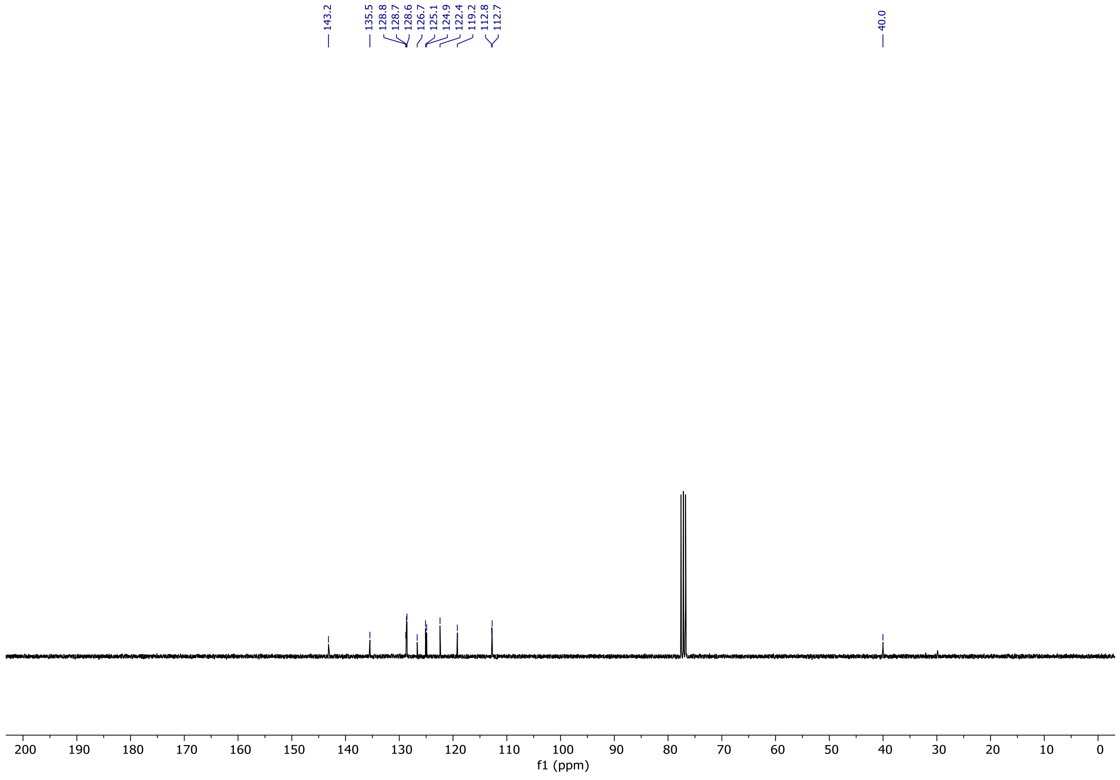
143.2
135.5
128.8
128.7
128.6
126.7
125.1
124.9
122.4
119.2
112.8
112.7
40.0
f1 (ppm)
